# Supramolecular trapping of a cationic all-metal σ-aromatic {Bi_4_} ring

**DOI:** 10.1038/s41557-024-01530-z

**Published:** 2024-05-17

**Authors:** Ravi Yadav, Avijit Maiti, Marcel Schorpp, Jürgen Graf, Florian Weigend, Lutz Greb

**Affiliations:** 1 Anorganisch-Chemisches Institut, Ruprecht-Karls-Universität Heidelberg, Heidelberg, Germany; 2 School of Chemistry, Indian Institute of Science Education and Research Thiruvananthapuram, Thiruvananthapuram, India; 3 Universität Regensburg, Regensburg, Germany; 4 Organisch-Chemisches Institut, Ruprecht-Karls-Universität Heidelberg, Heidelberg, Germany; 5 Fachbereich Chemie, Philipps-Universität Marburg, Marburg, Germany

**Keywords:** Organometallic chemistry, Molecular capsules, Chemical bonding

## Abstract

Aromaticity in organic molecules is well defined, but its role in metal-only rings remains controversial. Here we introduce a supramolecular stabilization approach of a cationic {Bi_4_} rhomboid within the symmetric charge sphere of two bowl-shaped dianionic calix[4]pyrrolato indinates. Crystallographic and spectroscopic characterization, quantum chemical analysis and magnetically induced ring currents indicate σ-aromaticity in the formally tetracationic 16-valence electron [Bi_4_]^4+^ ring. Computational screening for other *p*-block elements identifies the planar rhomboid as the globally preferred structure for 16-valence electron four-atomic clusters. The aromatic [Bi_4_]^4+^ is isoelectronic to the [Al_4_]^4−^, a motif previously observed as antiaromatic in Li_3_[Al_4_]^−^ in the gas phase. Thus, subtle factors such as charge isotropy seem to decide over aromaticity or antiaromaticity, advising for caution in debates based on the Hückel model—a concept valid for second-row elements but less deterministic for the heavier congeners.

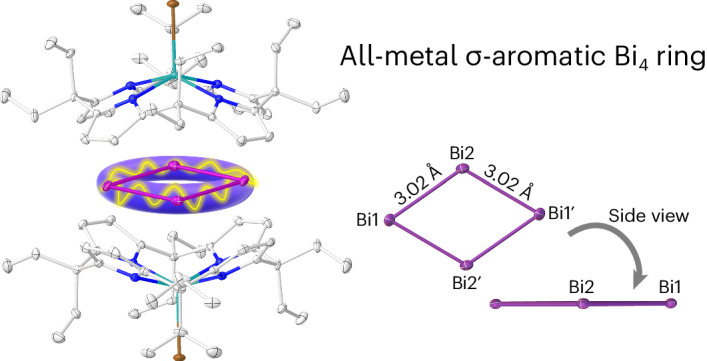

## Abstract (web summary)

The synthesis of cationic all-metal aromatic systems without covalent functionalization remains an underexplored area in chemistry. Now a tetracationic [Bi_4_]^4+^ featuring all-metal σ-aromaticity has been stabilized through a supramolecular approach relying on dianionic calix[4]pyrrolato indiumbromide shells. This planar rhomboid represents the global minimum for 16 valence electron systems.

## Main

**Fig. 1:**
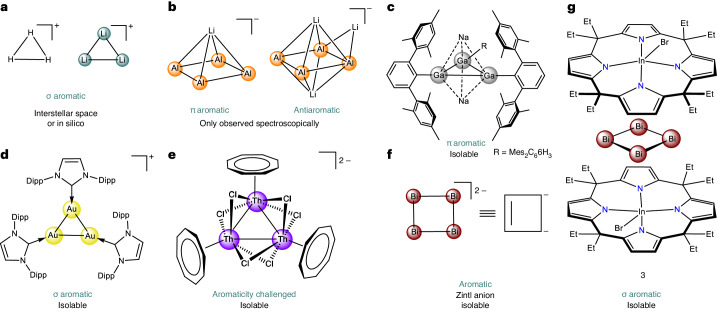
Examples of aromatic rings. **a**, Prototypical σ-aromatic cations. **b**, Gas phase observed aromatic and antiaromatic anions. **c**, Ligand-stabilized aromatic gallium ring. **d**,**e** Ligand-stabilized aromatic gold (**d**) and thorium rings (**e**). **f**, Aromatic Zintl anion. **g**, All-metal σ-aromatic formal [Bi_4_]^4+^ ring stabilized by supramolecular encapsulation.

Seminal investigations by Hückel on organic π-conjugated molecules established the particular stability of planar rings with (4*n* + 2)π electrons^1^. Since then, aromaticity developed as a cornerstone in chemistry^2,3^. Over the past decades, the concept extended from π-electrons to delocalized σ-, δ- and φ-electrons in organic and inorganic compounds along different dimensions^4–6^. Aromaticity in pure metal compounds (all-metal aromaticity) sparked particular interest^7,8^, and numerous all-metal aromatics were predicted from quantum theory^9^. One of the simplest in silico examples is the σ-aromatic Li_3_^+^, the all-metal analogue of H_3_^+^ featuring two delocalized σ-electrons (Fig. 1a)^10,11^. Spectroscopic detections of the aromatic Al_4_^2−^ and antiaromatic Al_4_^4−^ in molecular beams represented seminal experimental work that indicated the generality of Hückel’s rule in all-metal ring structures (Fig. 1b)^12,13^. Cases such as the aromatic Au_5_Zn^+^ detected by mass spectrometry or the planar Ti_3_(CO)_3_ cluster observed in a solid argon matrix consolidated this perspective^14,15^. Aromatic metal clusters were also suggested in Zintl phases but appear restricted to anions^16^. For instance, the square [Bi_4_]^2−^ has been regarded as an inorganic analogue of the prototypical π-aromatic [C_4_H_4_]^2−^ (Fig. 1f)^17^. More recent density functional theory (DFT) computations challenged the aromaticity in [Bi_4_]^2−^, but revealed a small degree of antiaromatic character that contrasts the lighter [Pn_4_]^2−^ congeners^18^. A case of π-aromaticity was determined in the heterometallic anion [Th@Bi_12_]^4−^, being only one example of the notable progress on bismuth clusters in recent years^18,19^.

Substantial insight on all-metal aromaticity was also gained with gallium-based three-, four- or five-membered ring systems with two π-electrons but that required stabilization by bulky terphenyl ligands (Fig. 1c)^20–22^. A recently reported thorium cluster extended σ-aromaticity towards actinides, while the electronic delocalization within the ring remained disputed (Fig. 1e)^23–25^. Aromatic systems in a cationic charge state are much less explored. Only mono- and di-cationic aromatic systems are known but have been restricted to compounds stabilized by large, covalent substituents^26–30^. For instance, a transition metal system with an aromatic Au_3_^+^ core coordinated by N-heterocyclic carbenes was presented (Fig. 1d)^31^. Unfortunately, any direct substitution perturbs the aromatic core’s electronic structure and impedes comparisons between experiment and the rich theoretical work on unperturbed ring systems^9^. Examples of cationic all-metal aromatic ring systems without covalent functionalization have not been reported. Notably, the highly electron-deficient nature of unsaturated cationic aromatics raised concerns about the chance of ever being isolated in the condensed phase^32^. Consequently, also the theoretical interest oriented more towards anions, while polycationic all-metal aromatics were rarely studied computationally^8,9^.

In this Article, we present a planar {Bi_4_} rhomboid trapped within two calix[4]pyrrolato indiumbromide shells (Fig. 1g). While supramolecular stabilization approaches proved effective for organic intermediates^33–35^, this strategy is far less developed for *p*-block elements^36,37^. The formally tetracationic [Bi_4_]^4+^ ring represents an isolable cationic all-metal aromatic ring without directly attached ligands. Interestingly, the [Bi_4_]^4+^ unit is isoelectronic to the gas-phase-observed Hückel-antiaromatic [Al_4_]_4_^−^.

## Results and discussions

### Synthesis and characterization

**Fig. 2:**
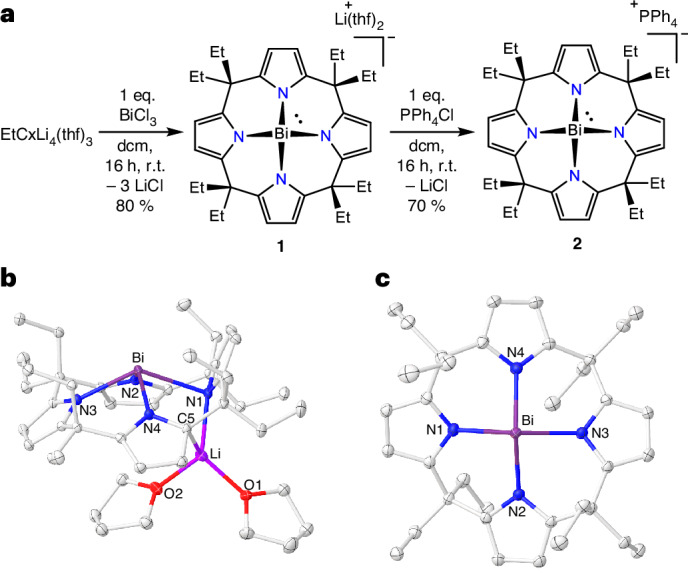
Synthesis and molecular structures of 1 and 2. **a**–**c**, Synthesis of **1** and **2** (**a**) and molecular structure of **1** (**b**) and **2** (**c**) in solid state. dcm, dichloromethane; r.t., room temperature. Hydrogen atoms and PPh_4_^+^ (for **2**) are omitted for clarity.

The reaction of the lithium salt of octaethylporphyrinogen [Li_4_·(thf)_3_EtCx] with BiCl_3_ in tetrahydrofurane (THF) resulted in the formation of [Li(thf)_2_EtCxBi] (**1**) in 80% yield (Fig. 2a). Single-crystal X-ray diffraction (scXRD) analysis of **1** revealed a Bi coordinated by four nitrogen atoms (Fig. 2b). The Li^+^ countercation is tetrahedrally coordinated by two thf, a nitrogen of one pyrrole ring and the α-C of the adjacent pyrrole ring. Despite the asymmetry in the solid state, the ^1^H-NMR spectrum of **1** in dichloromethane-d_2_ shows a singlet at *δ* = 6.28 ppm for the pyrrolyl β-hydrogen atoms, indicating the dynamic coordination of Li^+^ to the pyrrole rings. The Li^+^ can be replaced with non-coordinating PPh_4_^+^ by a metathesis reaction between **1** and PPh_4_Cl to obtain [PPh_4_][EtCxBi] (**2**), revealing a pseudo-C_4v_ symmetric anion with the Bi adopting a non-VSEPR square pyramidal geometry (Fig. 2c; see Supplementary Section 2.3).

**Fig. 3:**
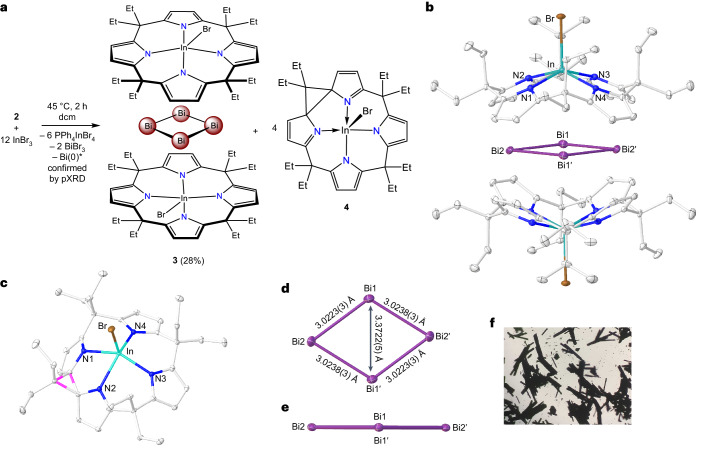
Synthesis and molecular structures of 3 and 4. **a**, Synthesis of compound **3** and **4**; *undefined stoichiometries of Bi metal formed. dcm, dichloromethane. **b**, The molecular structure of **3** in solid state. Hydrogen atoms are omitted for clarity. The {Bi_4_} ring is disordered over two positions. Two bismuth atoms are symmetry generated (inversion in the centre of the Bi4 rhomboid 1 − *x*, 1 − *y* and 1 − *z*; Supplementary Fig. 44). **c**, The molecular structure of **4** in solid state. Hydrogen atoms are omitted for clarity. Cyclopropyl ring is highlighted in red. **d**, Top view of the {Bi_4_} ring in **3** along with the Bi–Bi bond lengths. **e**, Side view of the {Bi_4_} ring in **3** showing the planarity. **f**, A microscopic photograph of the single crystals of **3**.

During our investigation of **2** as a transmetallation agent, we observed an unusual reactivity with InBr_3_. The reaction of **2** with InBr_3_ in dichloromethane resulted in a rapid colour change from light orange to dark red. After standing for 3 days at room temperature, dark-coloured crystals of **3** developed (Fig. 3f). The synthesis is reproducible, yielding up to 28% of isolated **3**. scXRD analysis showed a planar rhomboid {Bi_4_} ring encapsulated by two indiumbromide-calix[4]pyrrolates, [(Bi_4_)(EtCxInBr)_2_] (Fig. 3b). The In–Br (2.5355(4) Å) bond lengths in **3** are in line with literature-known bromidoindinates In–Br (2.5541(4)–2.5615(4) Å)^38^. The In–N (average 2.240(3) Å) are in the range of pentacoordinated indium centres, for example, (bis)amidinate indiumbromide (2.1727(18)–2.2503(19) Å)^39^. Each indiumbromide-calix[4]pyrrolate can be understood as dianionic, rendering the four-membered Bi ring formally tetracationic, with a formal oxidation state of each Bi atom as +1. The Bi–Bi bond lengths of the rhomboid are 3.0223(3) Å and 3.0238(3) Å, which are in the range of literature-known Bi(I)–Bi(I) bonds (2.970 to 3.038 Å)^40,41^.

The Bi1–Bi1′ distance (3.3772(5) Å) is substantially elongated, indicating only weak transannular interaction. The closest Bi–C_pyrrole_ distance in **3** is 2.982 Å, which is longer than reported Bi–C covalent bonds (around 2.26 Å)^42–46^. Despite the Bi–C distance in **3** being shorter than the sum of van der Waals radii (3.770 Å), these parameters indicate minor covalent interaction and justify the formal tetracationic classification as a [Bi_4_]^4+^ ring. This interpretation is further supported by bond analysis tools, as described in the Quantum chemical investigations section.

Compound **3** is insoluble in any solvent and could not be characterized in solution, but solid-state ^13^C- and ^15^N-MAS-NMR (MAS, magic-angle-spinning), infra-red and Raman spectroscopy are in line with the expected signatures and with the DFT-computed spectroscopic features. Elemental analysis of the isolated crystals of complex **3** gave carbon values consistently 8–10% lower than expected. We surmised the formation of bismuth metal covering the crystalline material as a source for this deviation. Indeed, powder XRD (pXRD) of the isolated solid showed the diffraction pattern of **3** (matching peaks with the calculated pattern; Supplementary Fig. 11), beside a species matching with metallic bismuth (Supplementary Fig. 12; http://www.crystallography.net/cod/9008576.html). pXRD analysis of the solid residue forming along with the crystals of **3** showed a diffraction pattern corresponding to pure bismuth metal only (Supplementary Fig. 13).

The planar, rhomboidal structure of [Bi_4_]^4+^ is surprising and contrasts with previous literature reports of four-membered Bi(I) systems. Indeed, the four-membered R_4_Bi_4_ structures exhibit a pronounced buckling in a butterfly shape^40,41^. In one of the first experimental reports on cationic bismuth clusters, the presence of a [Bi_4_]^4+^ was suggested from spectral studies on bismuth in liquid BiCl_3_, but the solid phase was consistently found to contain the [Bi_9_]^5+^ ion in a lower oxidation state^47^. As discussed in a later section, DFT computations identified the planar rhomboid as the global minimum structure also for the parent, free [Bi_4_]^4+^. However, before treating the electronic structure, the formation mechanism shall be discussed.

**Fig. 4:**
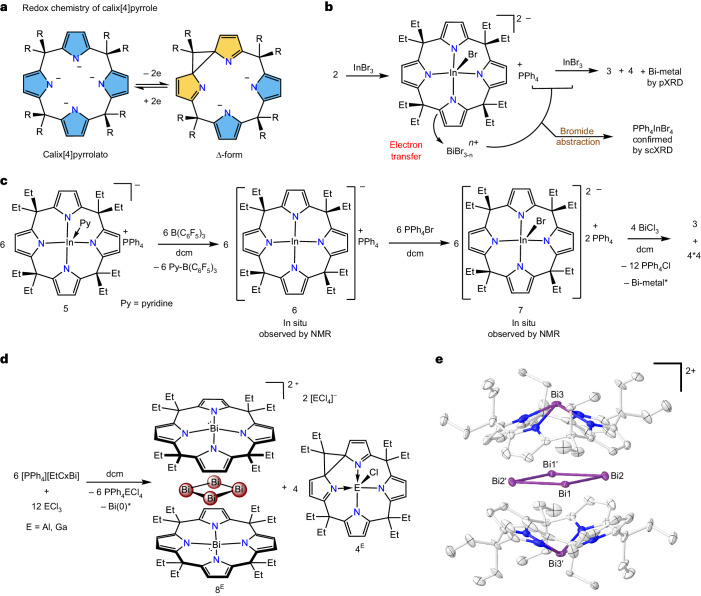
Underlying redox chemistry, formation mechanism, control experiments and derivatives of 3. **a**, Redox chemistry of the calix[4]pyrrole ligand. dcm, dichloromethane. **b**, Proposed reaction pathway for the formation of **3** and **4**. **c**, In situ generation of compound **7** and reaction with BiCl_3_ for the synthesis of **3** and **4** to support the proposed mechanism. **d**, Reaction of **2** with AlCl_3_ and GaCl_3_. **e**, The molecular structure of the cation in **8**^Al^ in solid state. Hydrogen atoms and anion are omitted for clarity. *Bi metal is formed due to the overreduction of Bi(I) species via other reaction channels.

Since the deposition of Bi metal was observed during the process, we assumed that [Bi_4_]^4+^ might represent an intermediate in metal formation that becomes stabilized as the side product **3** in the presence of indiumbromide-calix[4]pyrrolates. But what serves as the reducing agent? The redox chemistry of calix[4]pyrrolates has been of interest within the context of transition metal complexes^48–50^ and recently been extended by us to complexes of antimony^51^. The oxidation by 2e^−^ results in a so-called Δ-form, in which a cyclopropyl ring forms upon dearomatization of two pyrrole rings (Fig. 4a). Indeed, characterization of the supernatant of the reaction of **2** with InBr_3_ (Fig. 3a) revealed the formation of [ΔEtCxInBr] (**4**) and PPh_4_InBr_4_ as stoichiometric by-products by scXRD (Fig. 3c) and NMR spectroscopy (Supplementary Figs. 21–24; see Supplementary Section 1.2.3 for details). Hence, the formation of **4** revealed the calix[4]pyrrolato ligand as a source of the electrons for the reduction of Bi(III) over Bi(I) to elemental bismuth. The exact sequence of steps and inner-sphere versus outer-sphere electron transfer is impossible to distinguish, but the following is one plausible pathway. Transmetallation of Bi(III) with In(III) is furnishing [EtCxInBr]^2−^ and [BiBr_3−__*n*_]^*n*+^ (Fig. 4b). Dianionic [EtCxInBr]^2−^ reduces [BiBr_3−__*n*_]^*n*+^ to [Bi^I^Br_1−__*n*_]_*m*_^*n*+^ along with the formation of the two-electron oxidized [ΔEtCxInBr] (**4**).

The released bromide ions are captured by InBr_3_, as confirmed by the stoichiometric formation of PPh_4_InBr_4_. Alternatively, InBr_3_ might activate BiBr_3_ during the reduction process by bromide abstraction. Thus, from the perspective of the reducing agent [EtCxInBr]^2−^, the formation of [ΔEtCxInBr] **4** might also be described as a Lewis acid-assisted oxidative C–C coupling. Ultimately, the Bi(I)-cationic cluster forms and gets trapped within remaining bowl-shaped dianionic [EtCxInBr]^2−^. This reaction illustrates the multi-purpose nature of calix[4]pyrrolato ligand as both a mild reductant and supramolecular host.

To check whether the transmetallation product [EtCxInBr]^2−^ can reduce Bi(III) to Bi(I), we generated [PPh_4_]_2_[EtCxInBr] **7** in situ by an alternative synthesis and reacted with BiCl_3_ (Fig. 4c and Supplementary Section 1.2.4). Indeed, the formation of **3**, **4**, PPh_4_Cl and bismuth metal was observed, supporting the above-suggested pathway. Interestingly, [PPh_4_]_2_[EtCxInBr] **7** is thermally unstable and decomposes within 4 h in solution and within 5 days in solid state at room temperature. On the other hand, after encapsulating [Bi_4_]^4+^ in compound **3**, [EtCxInBr]^2−^ is stable at room temperature for months. Based on the redox potentials of **2** and **7** determined by cyclic voltammetry (Supplementary Section 1.2.6), **2** cannot be excluded to serve as an alternative reducing agent towards deliberated Bi(III). However, **2** did appear unreactive towards BiCl_3_, corroborating the idea that transmetallation is required to initiate the reaction sequence.

To confirm the generality of the formation of **3** and to obtain potential derivatives thereof, **2** was reacted with the lighter group 13 halides AlCl_3_ and GaCl_3_. Indeed, also in these cases, the formation of an encapsulated [Bi_4_]^4+^ species was observed, but the supramolecular cavity, this time composed of two monoanionic [EtCxBi]^−^ originating from starting materials **2**, with two additional AlCl_<sub4</sub_^−^ or GaCl_4_^−^ counteranions, respectively (**8**^**Al**^ and **8**^**Ga**^; Fig. 4d). At the same time, the corresponding transmetallated, oxidized products [ΔEtCxECl] **4**^**Al**^ and **4**^**Ga**^ and PPh_4_ECl_4_ (E = Al, Ga) were observed by NMR spectroscopy and high-resolution mass spectrometry (HR-MS) in the supernatant. The exclusive detection of oxidized **4**^**Al**^ and **4**^**Ga**^ (instead of corresponding oxidized Bi-containing compounds) further supports that the transmetallated dianions are favoured as reducing agents compared with **2**. It is interesting to note that literature-known pentacoordinated calix[4]aluminates and gallates that are isostructural to the putatively involved dianions [EtCxAlX]^2−^ and [EtCxGaX]^2−^ do not feature the bowl-shaped structure of [EtCxInBr]^2−^ but a ruffled conformation^52,53^ and are thus not suitable to trap [Bi_4_]^4+^. Corresponding trends were confirmed by the computation of related thermodynamics (Supplementary Section 3.6). Hence, supramolecular trapping requires a bowl-shaped species, which in this case represents [EtCxBi]^−^. Of note, the bond lengths inside the {Bi_4_} unit of a crystallographically characterized salt of a salt containing **8**^**Al**^ are similar to that in **3** (see comparison in Supplementary Section 2.3), despite the different charge state of the supramolecular host (−1 compared with −2 in **3**). This observation indicates that the electronic structure in {Bi_4_} does not strongly depend on the trapping sphere and supports the above-chosen description and discussion as [Bi_4_]^4+^.

### Quantum chemical investigations

For the analysis of the electronic structure, density functional calculations (Perdew–Burke–Ernzerhof (PBE) functional, basis sets of triple zeta valence quality) were performed for **3** and M_4_ rings (for details and references, see Supplementary Information). For all charged species, the conductor-like screening model was used. Natural population analysis on the experimental structure of **3** yields a charge of +0.69 |e| for Bi1 and Bi3 (Fig. 3) and +0.45 |e| for Bi2 and Bi4, and thus a total charge of +2.3 |e| for the Bi_4_ ring. Since these numbers are not observables but indicate directions, we perceive the idealized description as [Bi_4_]^4+^[L^2−^]_2_ with four Bi(I) as reasonable. From calculation and analysis of localized molecular orbitals (MOs), it is evident that the difference between ideal and calculated charge comes from a partial delocalization of eight pi-type bonds to empty orbitals of the Bi_4_^4+^ ring (Supplementary Section 3.5). For further corroboration, a topological investigation of the density for the [Bi_4_]^4+^[L^2−^]_2_ interaction was performed and compared with that of the pentamethyl cyclopentadiene anion (Cp*) with Bi^+^, indicating a higher ionic character for the title compound due to the total density and the ratio of its curvatures at the bond critical points (BCPs). Moreover, energy decomposition analysis yields a predominantly ionic nature in bonding (80%, as discussed in Supplementary Section 3.5). Further support for this assignment is the structural parameters of the computed global minimum for the [Bi_4_]^4+^ without any ligand sphere, yielding a rhombus with 298/313/508 pm for Bi1–Bi2/Bi2–Bi4/Bi1–Bi3 that agrees with the encapsulated ring structure in **3** (301/332/502 pm for the computed and 302/337/502 pm for the scXRD structure).

**Fig. 5:**
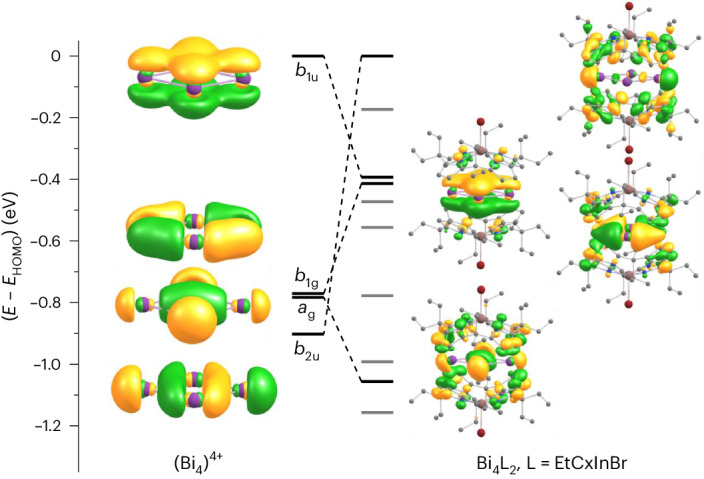
Highest occupied MOs in Bi_4_^4+^ and corresponding counterparts in 3. Contours are drawn at ±0.04 a.u.

For simplicity, we start the discussion of with the electronic structure of the isolated [Bi_4_]^4+^ rhombus. The four highest occupied MOs are shown in Fig. 5 (left).

The π-type highest occupied molecular orbital (HOMO) is the bonding linear combination of the 6*p* orbitals perpendicular to the plane, transforming like *b*_1u_ for the idealized *D*_2h_-symmetric structure. The following three orbitals are σ-type linear combinations of in-plane 6*p* orbitals, transforming like *b*_2u_, *b*_1g_ and *a*_g_. The *a*_g_ orbital is bonding between Bi2 and Bi4, and the *b*_2u_ orbital is antibonding between Bi1 and Bi3, rationalizing the rhomboid distortion. The four MOs arising from the Bi-6*s* orbitals are much lower in energy with *E* − *E*_HOMO_ amounting to −7.9/−8.5/−9.7/−11.2 eV.

**Fig. 6:**
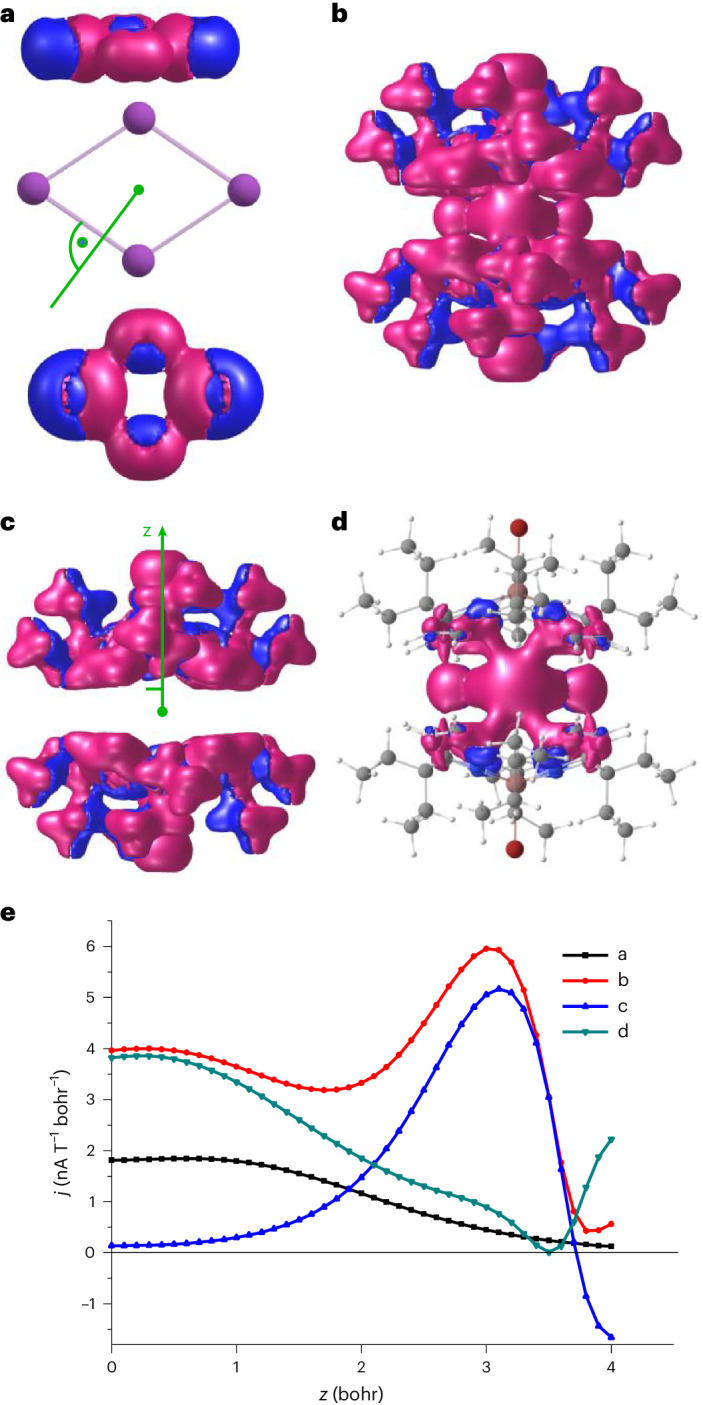
Induced ring-current analysis. **a**, Side and top view of absolute values of current densities in [Bi_4_]^4+^ (the green line indicates the orientation of the integration plane). **b**, Side view of absolute values of current densities in **3**. **c**, Side view of absolute values of current densities in [EtCxInBr]_2_^4−^ (the vertical line indicates the *z* direction, a horizontal line is drawn at 2 bohr. **d**, The difference between the current densities in **b** and **c**. Contours are drawn at 0.025 a.u, blue (red) contours indicate paratropic/diatropic areas, that is, areas with currents flowing counter-clockwise or clockwise. **e**, Current (*j*) profiles for **a**–**d** along the *z* direction defined above.

Four similar MOs are found for the encapsulated system in **3**, with HOMO and HOMO-1 being energetically interchanged (Fig. 5, right). These four MOs are intrinsically delocalized. If one applies a localization procedure to [Bi_4_]^4+^—we have chosen that of Pipek and Mezey as an example—one obtains an orbital that is practically identical to the HOMO (Supplementary Fig. 52). Consequently, the other three MOs result in three orbitals representing three-centre bonds distributed over the five Bi–Bi contacts. In such a situation, it is likely that the presence of a magnetic field will induce a ring current, which is an indication of aromaticity. Calculations of densities of induced magnetic ring currents from the response of the density to an external magnetic field perpendicular to the Bi_4_ ring (obtained with TURBOMOLE’s NMR module) via Biot–Savart’s law were done with the GIMIC tool (Fig. 6)^54,55^. The integration plane was chosen orthogonal to one of the four equivalent Bi–Bi bonds. Integration starts at the ring centre (green line in Fig. 6a, middle), and extends from 10 a.u. below to 10 a.u. above the ring plane. This way, a ring current of 9.1 nA T^−1^ is obtained for the isolated [Bi_4_]^4+^ (twice the area under the black curve in the current profile shown in Fig. 6e). The contribution of the π-type HOMO to the current can be estimated from the calculation of Bi_4_^6+^, where this orbital is unoccupied. For this system, one gets 6.5 nA T^−1^; thus, the π-contribution to the current in [Bi_4_]^4+^ is roughly one quarter only.

For comparison, the current in benzene, a typical π-aromat, is calculated to 11.4 nA T^−1^, and when removing the π-electrons (MOs *e*_1g_ and *a*_2u_) it vanishes (−0.1 nA T^−1^). Hence, electronic delocalization in the planar rhomboid of [Bi_4_]^4+^ is predominant in the σ-framework of the ring.

Does the situation change in the encapsulated ring in compound **3**? The absolute values of the current density of **3** are shown in Fig. 6b, that of the dianionic ligands only in Fig. 6c and that of the difference of both in Fig. 6d. Evidently, the ring current is maintained in the presence of the ligands, albeit somewhat extended to them. Integration over a plane extending from 2 a.u. below to 2 a.u. above the ring plane, which is a plausible choice according to Fig. 6c (green horizontal line), yields a current strength of 14.1 nA T^−1^ for the entire system. The contribution from the ligands in this region is 1.6 nA T^−1^. The difference of both, 13.2 nA T^−1^, corresponds to the current arising from the current density of the Bi_4_ ring in the presence of the ligands and is even higher than that of the isolated [Bi_4_]^4+^. The increase of currents and current densities in the presence of ligands is also reflected by nucleus-independent shifts (see Supplementary Information for details). For the bare [Bi_4_]^4+^ unit, they amount to ‒14.5 ppm at the ring centre and to ‒15.0 ppm at 1 a.u. above, whereas for the entire system **3**, one finds ‒34.2 and ‒31.6 ppm. Overall, the magnetic analyses indicate aromaticity in [Bi_4_]^4+^, within both the isolated and supramolecular trapped [Bi_4_]^4+^ unit. Indeed, in the experimental ^13^C-CP-MAS NMR (CP, cross-polarization) of **3**, unusually down-field shifted signals (>180 ppm) were observed. These shifts were reproduced by DFT computations and assigned to the pyrrole α-carbon atoms closest to the bismuth ring. The unusual shifts disappear upon replacing the [Bi_4_]^4+^ with 4 Xe atoms (Supplementary Section 3.7). Hence, this observation might be a spectroscopic indication of all-metal aromaticity, but further experiments and derivatives are required for a conclusive statement.

To inspect the generality of the rhomboid structural motif, we compared [Bi_4_]^4+^ with isoelectronic Pb_4_ and [Tl_4_]^4−^ and with their lighter homologues from rows 3p to 5p. All results hold when including spin–orbit coupling and changing the functional to PBE0. Details are documented in Supplementary Information; we summarize the main results here. For all 12 species, the global minimum is the rhombic ring with an electronic singlet state. Stationary points with quadrangular shape (triplets) or rectangular shape (singlets) were calculated as saddle points. Further local minima are tetrahedra (singlets), disfavoured by 62 kJ mol^−1^ [Tl_4_]^4−^ to 317 kJ mol^−1^ ([P_4_]^4+^), and butterfly structures (triplets), disfavoured by 17 kJ mol^−1^ [Tl_4_]^4−^ to 136 kJ mol^−1^ ([As_4_]^4+^). For the rhombic arrangements of [Al_4_]^4−^, [Ga_4_]^4−^, [In_4_]^4−^ and [Tl_4_]^4−^, which show comparably small HOMO–LUMO gaps (where LUMO is the lowest unoccupied molecular orbital) for the singlet state, additional triplet state minima are found, but were disfavoured by 11 kJ mol^−1^ to 14 kJ mol^−1^. Ring currents for the rhombic structures range from 5.2 nA T^−1^ ([Tl_4_]^4−^) to 14.8 nA T^−1^ ([Al_4_]^4−^), reflecting moderate to substantial aromatic character. Hence, the aromatic rhombic structure is favoured for all 16-valence electron (VE) M_4_ species, including Al_4_^4−^. This latter ion has been observed in the gas phase by mass spectrometry as Li_3_Al_4_^−^ (ref. ^13^). But why has it been described as a rectangular antiaromat? Indeed, the rectangular topology of [Al_4_]^4−^ becomes a minimum in the anisotropic sphere of four Li^+^ and the current is overall paratropic, −5.0 nA T^−1^, reflecting weak antiaromaticity. The latter comparison offers an interesting conclusion: within an anisotropic Coulomb field (for example, in Li_3_[Al_4_]^−^), the antiaromatic rectangular is a minimum for 16VE M_4_ but an aromatic rhomboid minimum within a spherical (more isotropic) charge sphere (for example, as Al_4_^4−^ in a dielectric continuum, or as [Bi_4_]^4+^ observed in isoelectronic **3**).

## Conclusions

Here, we describe the isolation and characterization of a cationic all-metal σ-aromatic ring, the formally tetracationic {Bi_4_}. The highly electron-deficient unit is trapped as compound **3** in a highly symmetric charge field of a cavity spanned by two π-electron-rich calix[4]pyrrolato units. The similarity of the bowl-shaped, dianionic calix[4]pyrrolato indinate to the versatile host corannulene is to be noted^56^. The reduction of a Bi(III) precursor to the Bi(I) containing **3** occurs along with the formation of elemental bismuth, and it can be speculated whether [Bi_4_]^4+^ represents a rare snapshot during metal formation^57,58^. Interestingly, the reduction cascade is initiated by adding a Lewis acid, while the reducing equivalents originate from the electron-rich ligand—contrasting with common strategies based on reducing agents. The planar rhomboid of [Bi_4_]^4+^ is identified as the preferred structure for all isoelectronic four-atomic 16VE *p*-block element systems, providing rich opportunities to extend experimental and theoretical directions in metal-cluster design. Further, this study indicates that changes in the charge distribution around isoelectronic ionic metal rings can influence the preference over aromatic (rhomboid 16VE systems in symmetric charge sphere, for example, **3**) versus antiaromatic (rectangular in asymmetric charge sphere, for example, Li_3_[Al_4_]^−^) minima. These subtle effects might provide new opportunities to control electron delocalization or charge carrier mobility with implications for materials design. From a more fundamental perspective, these insights question if excessively deterministic discussions on aromaticity, non-aromaticity or antiaromaticity are always reasonable, or if the projection of Hückel’s model finds its limitation if leaving from the second-row elements towards the heavier analogues.

## Online content

Any methods, additional references, Nature Portfolio reporting summaries, source data, extended data, supplementary information, acknowledgements, peer review information; details of author contributions and competing interests; and statements of data and code availability are available at 10.1038/s41557-024-01530-z.

## Supplementary information

Supplementary Information Supplementary Figs. 1–55, discussion and Tables 1–13.

Supplementary Data 1 Crystallographic data for compound 1; CCDC reference no. 2264827.

Supplementary Data 2 Crystallographic data for compound 2; CCDC reference no. 2264830.

Supplementary Data 3 Crystallographic data for compound 3; CCDC reference no. 2264829.

Supplementary Data 4 Crystallographic data for compound 4; CCDC reference no. 2264828.

Supplementary Data 5 Crystallographic data for compound 8_AlOAl; CCDC reference no. 2334621.

Supplementary Data 6 Cartesian coordinates of all DFT computed structures.

## Data Availability

All data generated or analysed during this study are included in this manuscript and its supplementary information files. The structures of compounds **1**, **2**, **3**, **4** and **8**^**AlOAl**^ were determined by scXRD. Crystallographic data for the structures reported have been deposited at the Cambridge Crystallographic Data Centre, under deposition numbers CCDC 2264827 (**1**), 2264830 (**2**), 2264829 (**3**), 2264828 (**4**) and 2334621 (**8**^**AlOAl**^). Copies of the data can be obtained free of charge via https://www.ccdc.cam.ac.uk/structures/. Details of the quantum chemical calculations can be found in Supplementary Information together with the relevant references to the methods used and information on their availability. The optimized Cartesian coordinates of all compounds mentioned explicitly in the main text or Supplementary Information are provided in a supplementary data file.
