# Supplementary material for: Supramolecular trapping of a cationic all-metal σ-aromatic {Bi4} ring
Source: Nat Chem. 2024 May 17;16(9):1523–30. doi: 10.1038/s41557-024-01530-z (PMC11374680; doi:10.1038/s41557-024-01530-z)
Supplement: Supplementary file 1 — Supplementary Figs. 1–55, discussion and Tables 1–13. [file 41557_2024_1530_MOESM1_ESM.pdf]

nature chemistry

Article

https://doi.org/10.1038/s41557-024-01530-z

Supramolecular trapping of a cationic all-metal σ-aromatic $\{Bi_4\}$ ring

In the format provided by the authors and unedited

Table of Contents

1. Experimental Section 2
1.1. General Methods 2
1.2. Synthesis and characterizations 4
1.2.1. Synthesis of Lithium *meso*-octaethylcalix[4]pyrrolato bismuthate [Li(thf)$_2$EtCxBi] (1) 4
1.2.2. Synthesis of tetraphenylphosphonium *meso*-octaethylcalix[4]pyrrolato bismuthate [PPh$_4$][EtCxBi] (2) 8
1.2.3. Synthesis of complexes 3 and 4. 11
1.2.4. Second route for the synthesis of 3 and 4 for mechanism studies 22
1.2.5. In-situ generation of complex 6 and 7 and reaction with BiCl$_3$ 25
1.2.6. Cyclic Voltammetry Experiment 29
1.2.7. Reduction of 3 with KC$_8$ under ball milling condition 31
1.2.8. Synthesis of 8[E] 32
1.2.9 Synthesis of meso-octaethylcalix[4]pyrrole (H$_4$EtCx) 36
1.2.10 Synthesis of tetra-lithium-meso-octaethylcalix[4]pyrrolato [Li$_4$·(thf)$_3$EtCx][2] 37
2. X-Ray Diffraction 39
2.1. Supplementary Table 1. Crystal data and structural refinements 40
2.2. X-ray structures 41
2.3. Discussion of selected metric parameters 45
3. Quantum chemical calculations 46
3.1. General Comments 46
3.2. Computational Data 47
3.3. Computed UV-Vis spectrum of 3 50
3.4. Vibrational Spectroscopy 51
3.5. Bond analysis: Localized MOs, Bond-critical points, energy decomposition analysis and a simple electrostatic model 51
3.6. Deformation of “ruffled” to “domed” calix[4]pyrrolates 54
3.7. Influence of the Bi$_4$ unit on the ^{13}C shifts 55
4. References 56

1. Experimental Section

1.1. General Methods

All used reagents and solvents were purchased from commercial sources. Unless otherwise noted, all manipulations were carried out under a dry nitrogen or argon atmosphere. Solvents were degassed prior to use with three freeze-pump-thaw cycles and were stored in sealed Schlenk ampoules over activated molecular sieve (3 or 4 Å, respectively) under a dry argon atmosphere. Liquid reactants were degassed for at least 10 minutes with a constant stream of dry argon through the fluid phase and were dried by storage over activated molecular sieve (3 or 4 Å, respectively). Solid reagents were dried and purified if necessary either by the application of vacuum and elevated temperature, or by sublimation under reduced pressure at elevated temperature. All reactions on preparative scale were carried out in flame-dried standard laboratory glassware under a dry argon atmosphere using Schlenk line techniques. Syringes, magnetic stirring bars, and needles were dried and/or flushed with argon prior to use. Reaction on the NMR sample scale were done in dry J. Young NMR tubes. Compounds sensitive to ambient conditions were handled and stored in a Sylatech glove box filled with dry nitrogen gas. Removal of solvents *in vacuo* was performed using a Schlenk line. Literature-known compounds H_4EtCx,[1] [Li_4·(thf)$_3$EtCx],[2] were synthesized following published procedures and the details for the synthesis are mentioned in section 1.2.9 and 1.2.10.

Nuclear magnetic resonance (NMR) spectra were collected with a Bruker BZH 200/52, a Bruker DPX 200, a Bruker Avance II 400, or a Bruker Avance III 600 spectrometer at 298 K unless otherwise noted. Measurements with the Bruker Avance spectrometers were carried out by the NMR facility of the Institute of Inorganic Chemistry of the University of Heidelberg. Chemical shifts δ are given in parts per million (ppm) relative to the tetramethylsilane resonance. ^{1}H NMR data is reported as follows: chemical shift δ [ppm], multiplicity (s = singlet, br s = broad singlet, d = doublet, t = triplet, q = quartet, quint. = quintet, sept = septet, m = multiplet), scalar spin-spin coupling constant [Hz] as $^XJ_{AB}$ (X = number of chemical bonds between coupled nuclei; A, B = coupled nuclei), integration value, signal assignment. ^{13}C{^{1}H} NMR data is reported as follows: chemical shift δ [ppm], multiplicity (only for $^XJ_{CP}$, d = doublet), type of carbon atom (CH_3, CH_2, CH, C_q), signal assignment. The protons of the aromatic pyrrole rings of the calix[4]pyrrolato ligand are denoted "β-H", the respective carbon atoms "β-C". The quaternary carbon atoms of the aromatic pyrrole rings in the ligand are named "C_q-

pyrrole". The atoms of the ligand's Ethyl groups are called "α-Et, -CH_2-" or "α-Et, -CH_3" respectively and the quaternary carbon atoms to which they are attached "α-C".

Solid state NMR experiments were conducted on a Bruker Avance III 500 MHz NMR spectrometer equipped with a standard bore (54 mm room temperature bore) 11.7 T magnet. A double resonance magic angle sample spinning (MAS) probe was used for 4.0 mm spinners. The probe is doubly tuned to the frequency of the observe nucleus – ^{13}C and ^{15}N at 125.78 MHz and 50.69 MHz, respectively – and to the frequency of ^{1}H at 500.22 MHz. Chemical shift referencing was done by the substitution method following the IUPAC recommendations: ^{13}C chemical shifts were calibrated setting the CH_2-signal position in adamantane to 37.77 ppm and calculating the ^{15}N reference frequency using the tabulated values (10,136767 for ^{15}N und 25.145020 for ^{13}C). Following this procedure ^{15}N chemical shifts refer to the IUPAC recommended standard $MeNO_2$. During the NMR measurements the rotor was spun at the magic angle (54.7°) at a rotation frequency of 10 kHz for ^{13}C (if not stated otherwise) and 5 kHz for ^{15}N. The sample temperature was not regulated. The CP-MAS NMR spectra were measured with the Bruker standard pulse program cp with a ramped contact time of 2 ms and a recycle delay of 5 s for ^{13}C. For ^{15}N a ramped contact time of 5 ms and a recycle delay of 2s was applied. A spinal64 proton broadband decoupling with a RF field of 100 kHz were used. For the ^{13}C CP-MAS NMR spectrum 10.240 scans and for the ^{15}N CP-MAS NMR spectrum 495.175 scans were accumulated, respectively.
Powder X-ray diffraction data were recorded with a Stoe Stadi P [Cu-K_α radiation, λ= 1.5406 Å, Ge(111)-monochromated] in sealed glass capillaries as sample containers (Ø = 0.5 mm). Further data processing was done by using software such as Profex,[3] QualX,[4] and Origin-Pro.
X-band EPR spectra (~9.6 MHz) were recorded with a spectrometer (Bruker Elexsys E500 EPR) with a continuous wave dual-mode resonator (Bruker ER 4116DM).

CV measurements were investigated with the EmStat3+ Blue from PalmSens Compact Electrochemical Interfaces. The program PSTrace 5.7 was used to record all measurements. The experiments were conducted in the glovebox SylaTech Y05G under N_2 atmosphere in a glass cell using a three-electrode configuration. A glassy carbon electrode, with a working area of 0.07 cm^2, was used as working electrode, a platinic wire as counter electrode; and a silver wire served as quasi reference electrode. As internal standard ferrocene was measured at the very end of each measurement. The spectra were plotted with OriginPro 2019 (9.6.0). The CV

spectra were collected at room temperature at a scan rate of 100 mV/s in dichloromethane, unless otherwise stated. The electrolytes of choice were NBu_4PF_6. The electrolyte was dried overnight under reduced pressure and stored in the glovebox. The concentration of the electrolyte solution was 0.1 M in a total electrolyte volume of 6 mL. The solutions were stirred between each CV measurement and kept under N_2 atmosphere throughout the measurements.

Solid sample of **3** were prepared and encapsulated inside of a glovebox. Raman spectra of these films were acquired with a Renishaw inVia confocal Raman microscope (633 nm excitation), in backscattering configuration, equipped with a 50× long working distance objective (N.A. 0.5, Olympus). To minimize the influence of spot-to-spot variations, maps with >200 spectra were recorded and averaged.

1.2. Synthesis and characterizations

1.2.1. Synthesis of Lithium *meso*-octaethylcalix[4]pyrrolato bismuthate [Li(thf)$_2$EtCxBi] (1)

[Li_4·(thf)$_3$EtCx] (2.5 g, 3.20 mmol) and $BiCl_3$ (1.01 g, 3.20 mmol) were added to a Schlenk flask. To this solid mixture, 30 mL of pre-cooled (-40 °C) thf was added. The reaction mixture was stirred at room temperature for 16 h. Then all the volatiles were removed under vacuum. 50 mL of 2:1 mixture of dcm/hexane was added to the residue and stirred for 1 hour and filtered. The filtrate was dried *in-vacuo* and the red solid was washed with 5*2 mL of cold pentane. The product was dried under vacuum. The crystals for X-ray diffraction were grown by cooling (-40 °C) a saturated solution **1** in pentane.

Yield: 2.30 g (80 %, 2.56 mmol). **Elem. Anal.** for **[Li(thf)$_2$EtCxBi]·**calculated: C, 58.92; H, 7.19; N, 6.25. Found: C, 58,89; H, 7.01; N, 6,31.

^{1}H NMR (600 MHz, CD_2Cl_2, 298 K): δ= 6.28 (s, 8H, C*H*, β-C), 3.49 (m, 8H, C*H*$_2$-thf), 1.90 (q, $^3J_{HH}$ = 7.3 Hz, 8H, C*H*$_2$-ethyl), 1.81 (q, $^3J_{HH}$ = 7.2 Hz, 8H, C*H*$_2$-ethyl), 1.73 (m, 8H, C*H*$_2$-thf), 0.74 (t, $^3J_{HH}$ = 7.4 Hz, 12H, C*H*$_3$-ethyl), 0.62 (t, $^3J_{HH}$ = 7.3 Hz, 12H, C*H*$_3$-ethyl).

^{13}C{^{1}H} NMR (151 MHz, CD_2Cl_2, 298 K): δ= 143.7 (*C$_q$*, C$_q$-pyrrole), 109.5 (*C*H, [illegible]-C), 68.5 (*C*H$_2$-thf), 46.7 (*C$_q$*, α-C), 38.0 (*C*H$_2$-ethyl), 34.5 (*C*H$_2$-ethyl), 25.8 (*C*H$_2$-thf), 9.8 (*C*H$_3$-ethyl), 9.7 (*C*H$_3$-ethyl).

^{7}Li NMR (233 MHz, CD_2Cl_2, 298 K): δ = -2.21.

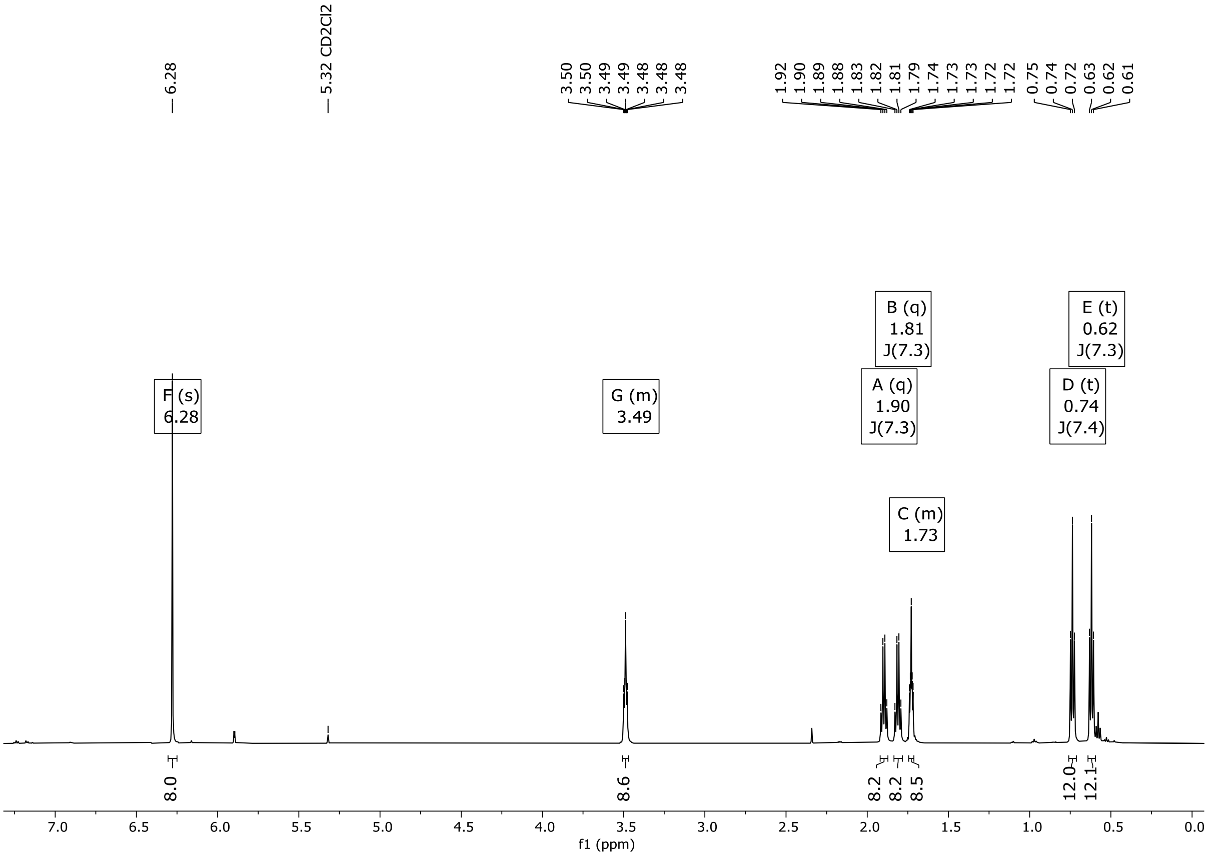

Supplementary Figure 1. ^{1}H NMR (600 MHz, 298 K, CD_2Cl_2) spectrum of compound **1**.

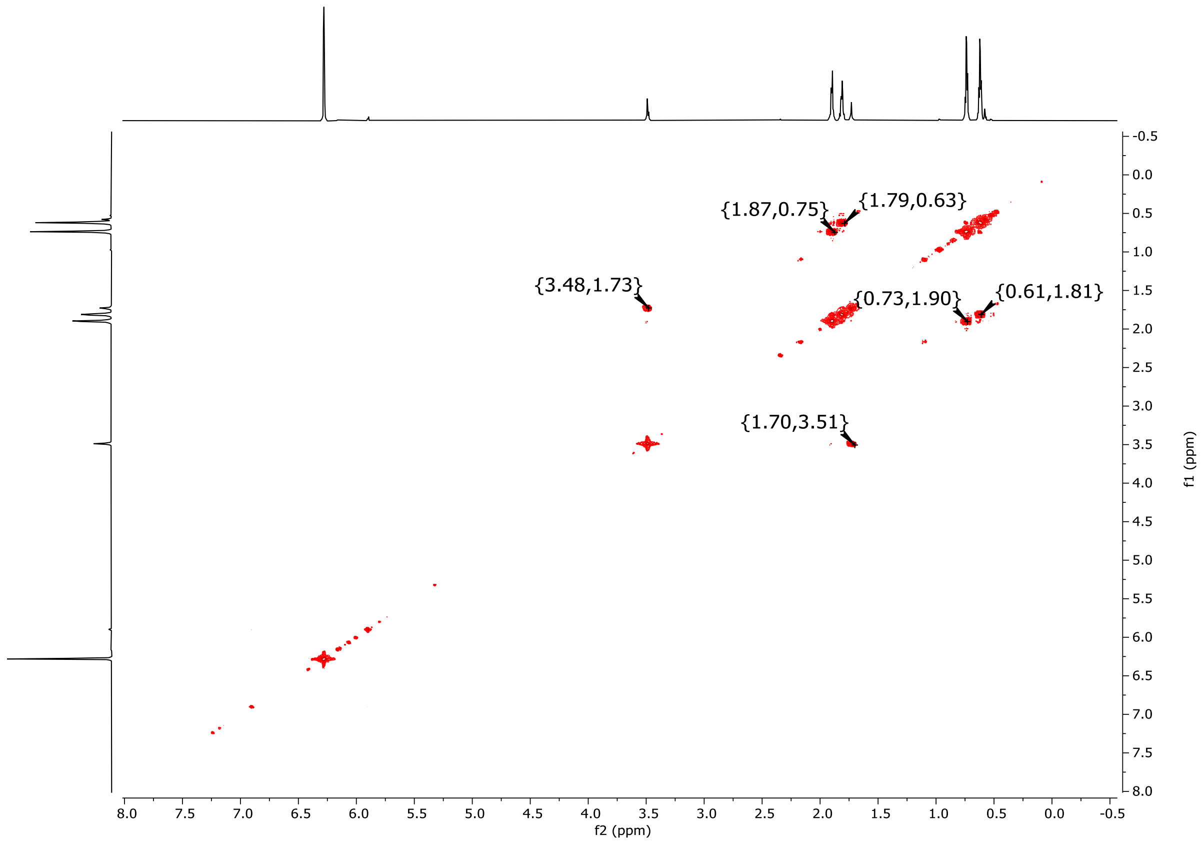

Supplementary Figure 2. ^{1}H-^{1}H COSY NMR (600 MHz, 298 K, CD_2Cl_2) spectrum of compound **1**.

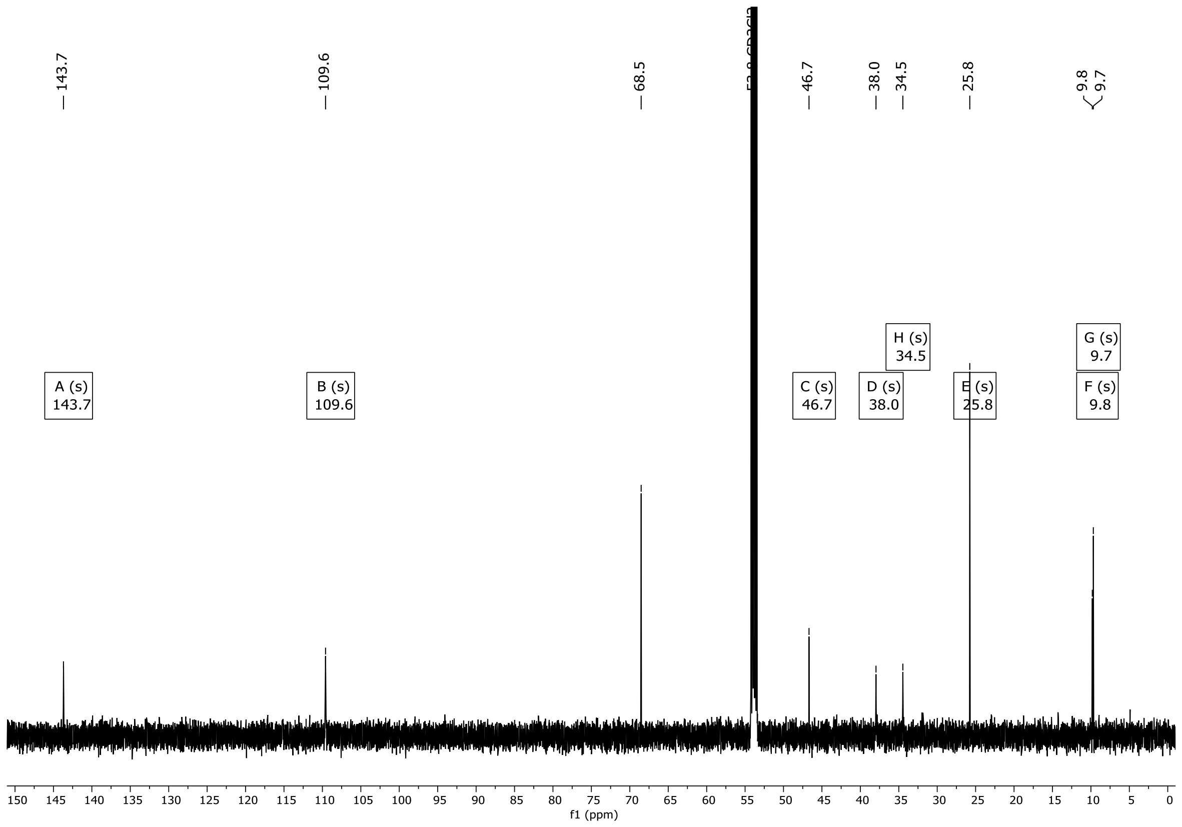

Supplementary Figure 3. $^{13}C\{^{1}H\}$ NMR (151 MHz, 298 K, CD_2Cl_2) spectrum of compound **1**.

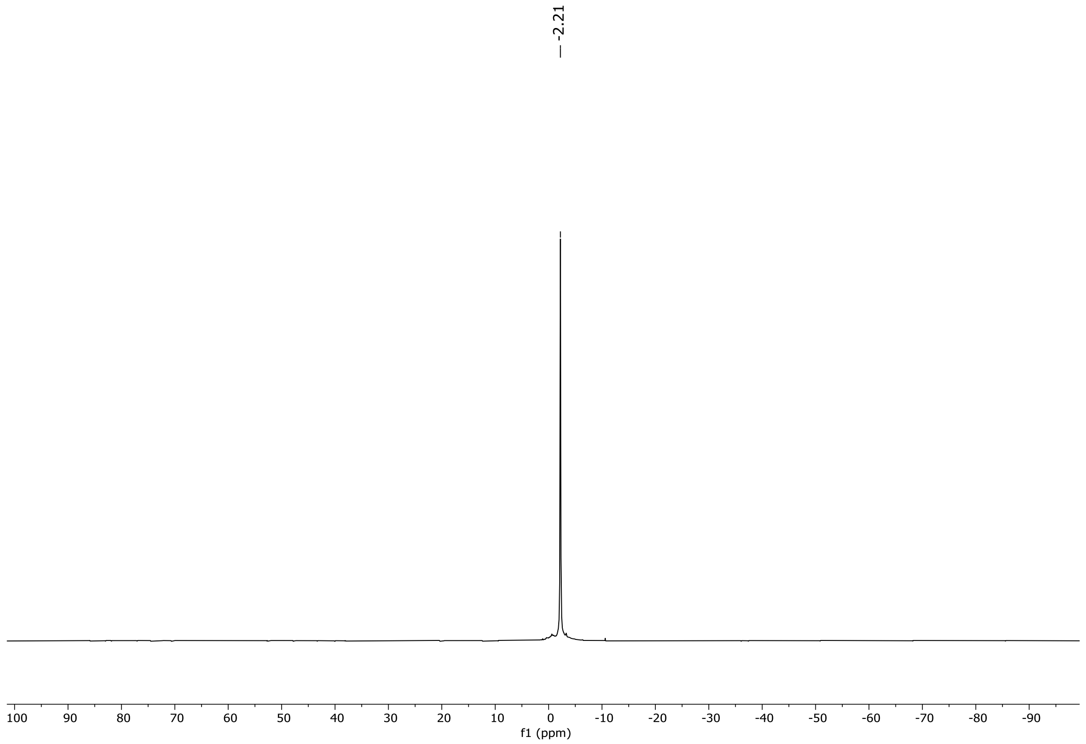

Supplementary Figure 4. ^{7}Li NMR (233 MHz, 298 K, CD_2Cl_2) spectrum of compound **1**.

1.2.2. Synthesis of tetraphenylphosphonium *meso*-octaethylcalix[4]pyrrolato bismuthate [PPh_4][EtCxBi] (2)

[Li(thf)$_2$EtCxBi]·(**1**) (3 g, 3.34 mmol) was dissolved in 30 mL of dichloromethane and PPh_4Cl (1.25 g, 3.34 mmol) was added as a solid to the solution. The reaction mixture was stirred for 16h at room temperature. The solvent was removed under vacuum. The solid obtained was dissolved in 35 mL (30 mL dcm with 5 mL pentane) and stirred for 6 hours to allow complete precipitation o LiCl. The reaction mixture was filtered and all the volatiles were removed from the filtrate. The solid obtained was washed with 2*10 mL of hexane/pentane and dried under vacuum. The crystals for X-ray diffraction were grown from vapour diffusion of pentane in a dichloromethane solution of **2**.

Yield: 2.70 g (82%, 2.49 mmol). **Elem. Anal.** for **[PPh_4][EtCxBi]·(dcm)·**calculated: C, 62.62; H, 6.03; N, 4,79. Found: C, 62.96; H, 5,84; N, 5,34. Compound **2** always contains 1 to 0.5 equivalent of dcm, as seen in the ^{1}H NMR (even after drying overnight under high vacuum) (Supplementary Figure 5) and also in XRD.

^{1}H NMR (600 MHz, CD_2Cl_2): δ = 7.90–7.84 (m, 4H, PPh_4^+), 7.73–7.67 (m, 8H, PPh_4^+), 7.60–7.53 (m, 8H, PPh_4^+), 6.02 (s, 8H, C*H*, β-C), 1.90 (q, *J* = 7.3 Hz, 8H, C*H*$_2$-ethyl), 1.84 (q, *J* = 7.2 Hz, 8H, C*H*$_2$-ethyl), 0.81 (t, *J* = 7.3 Hz, 12H, C*H*$_3$-ethyl), 0.64 (t, *J* = 7.2 Hz, C*H*$_3$-ethyl).

^{13}C NMR (151 MHz, CD_2Cl_2): δ = 142.7 (C_q, C_q-pyrrole), 136.2 (d, PPh_4^+, $^4J_{CP}$ = 3.1 Hz), 134.7 (d, PPh_4^+, $^2J_{CP}$ = 10.3 Hz), 131.0 (d, PPh_4^+, $^3J_{CP}$ = 12.9 Hz), 117.8 (d, PPh_4^+, $^1J_{CP}$ = 89.6 Hz), 105.0 (*C*H, β-C), 46.5 (C_q, α-C), 37.3 (*C*H$_2$-ethyl), 37.2 (*C*H$_2$-ethyl), 10.5 (*C*H$_3$-ethyl), 10.4 (*C*H$_3$-ethyl).

^{31}P{^{1}H} NMR (243 MHz, CD_2Cl_2): δ = 23.25.

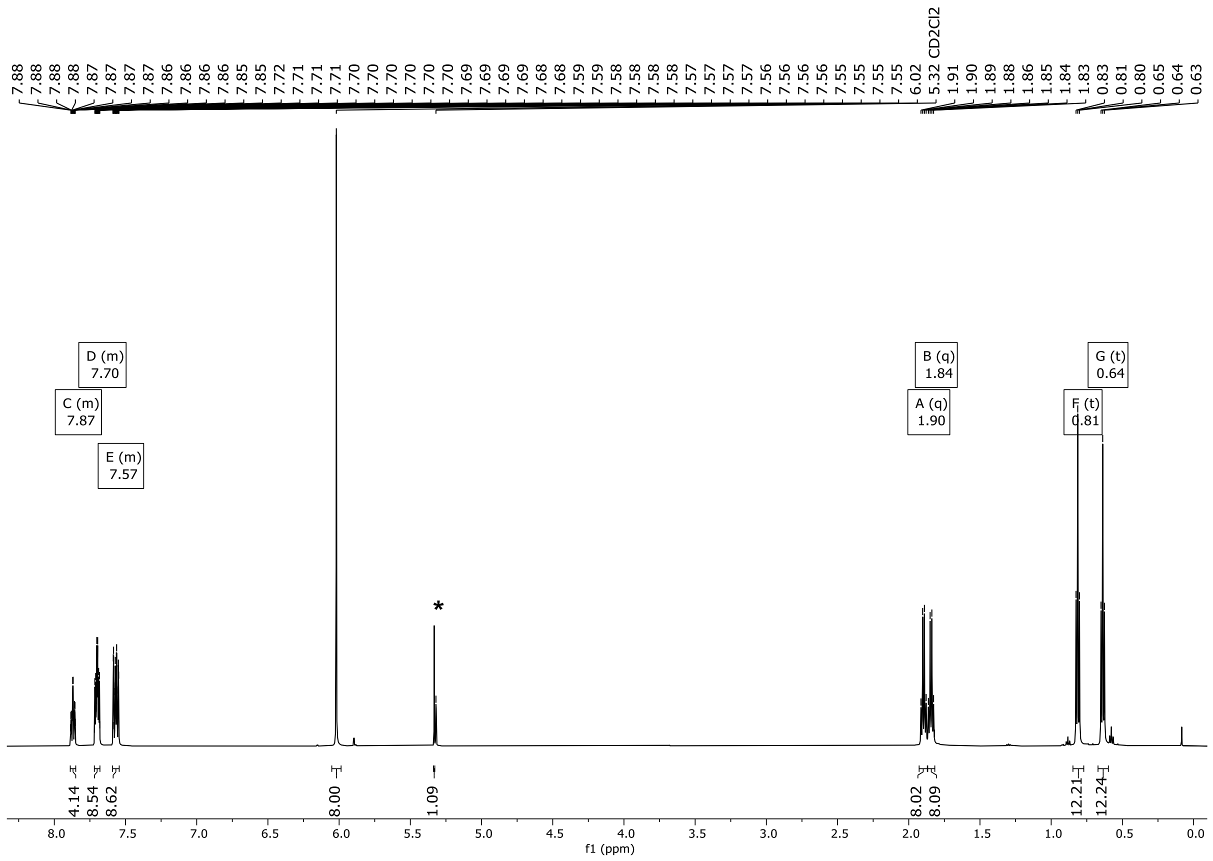

Supplementary Figure 5. ^1H NMR (600 MHz, 298 K, CD_2Cl_2) spectrum of compound **2**. * = residual one equivalent of dichloromethane.

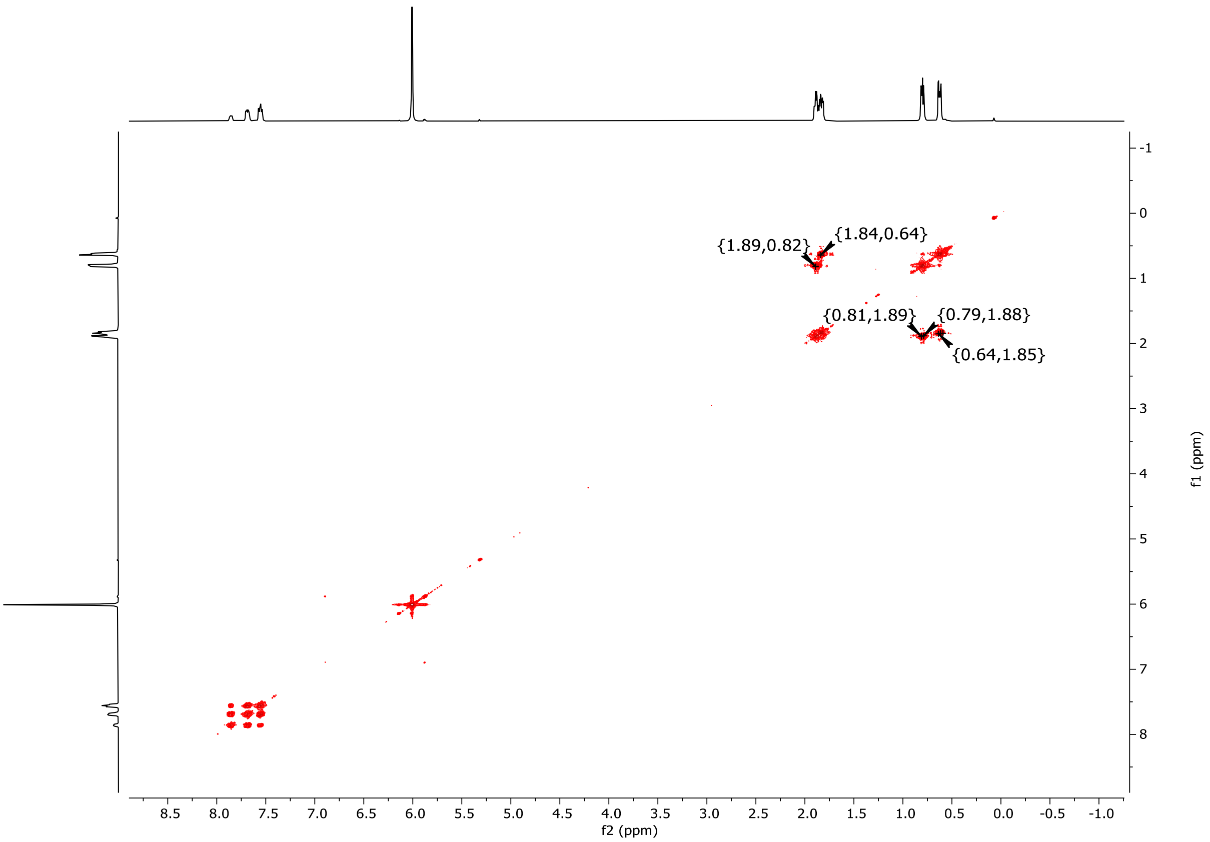

Supplementary Figure 6. ^{1}H-^{1}H COSY NMR (600 MHz, 298 K, CD_2Cl_2) spectrum of compound **2**.

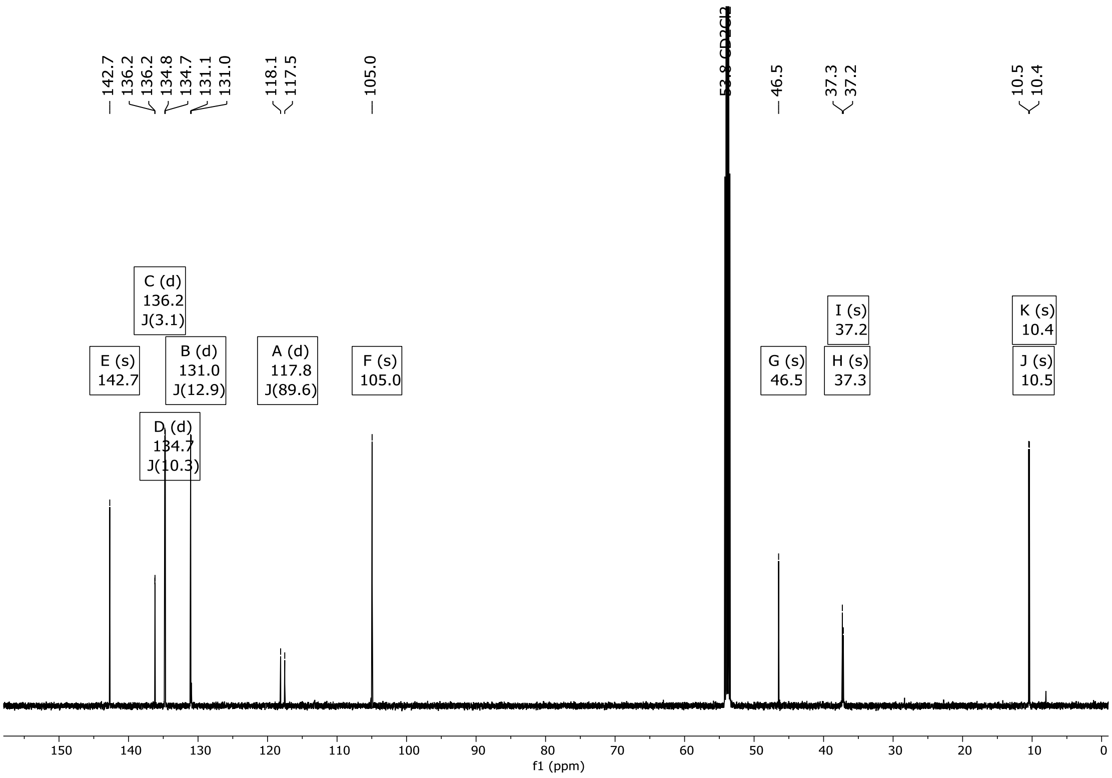

Supplementary Figure 7. $^{13}C\{^{1}H\}$ NMR (151 MHz, 298 K, CD_2Cl_2) spectrum of compound **2**.

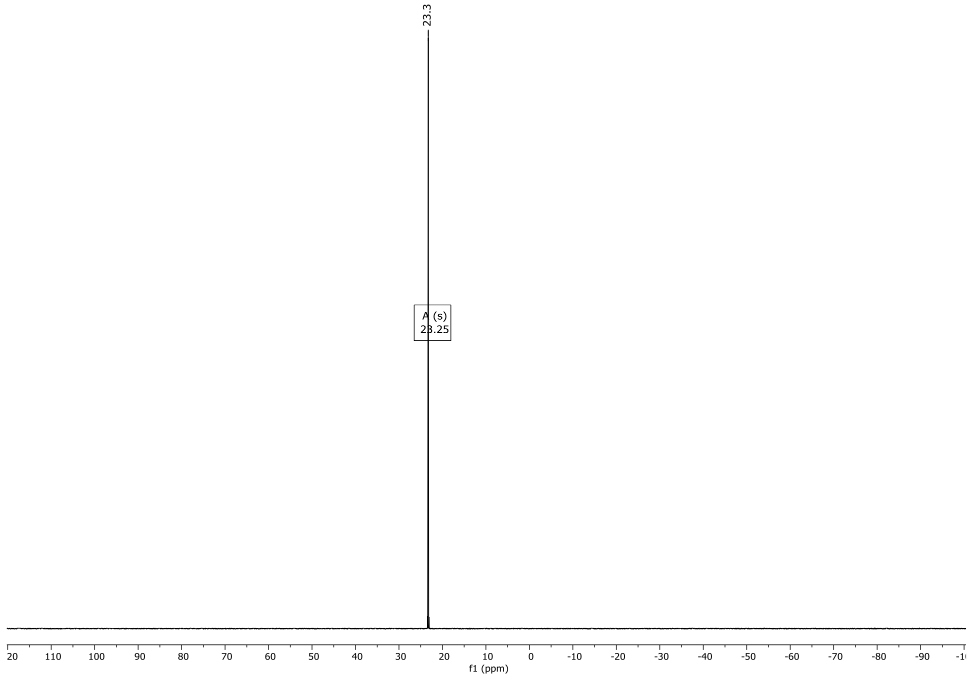

Supplementary Figure 8. $^{31}P\{^{1}H\}$ NMR (243 MHz, 298 K, CD_2Cl_2) spectrum of compound **2**.

1.2.3. Synthesis of complexes 3 and 4.

To a solution of **2** (250 mg, 0.214 mmol) in dcm (5 mL), $InBr_3$ (114 mg, 0.320 mmol) was added. The reaction mixture was sonicated for 10 minutes and then heated at 45 °C for 2 hours without stirring. The heating was turned off, and the reaction tube was allowed to stand for 1 week without disturbing. During this time, the reaction color changed to wine red and dark-colored crystals of compound **3** were deposited. The mother liquor consists of **4** and PPh_4InBr_4, which were separated (see below). The deposited crystals were separated by filtration (workup of filtrate given in next paragraph). The crystals of **3** were washed with pentane, toluene, dcm, thf (each 2*3 mL) to remove any impurities. Compound **3** is not soluble in any solvent; this could be attributed to the large size of the molecule and its low flexibility.

Yield of sample of **3**: 30 mg (calculated yield of **3** given below). Characterization of **3** in solution was impossible due to the insolubility in common organic solvents.

^{13}C MAS NMR (125.78 MHz): 187.84 (C_q), 180.96 (C_q), 148.01 (C_q), 145.60 (C_q), 108.28 (β-C), 107.25 (β-C), 104.56 (β-C), 66.88 (DCM), 44.64 (α-CH_2), 43.7 (Cq, α-C), 35.18 (α-CH_2), 29.68(

α-CH$_2$), 27.89 (α-CH$_2$), 25.27 (α-CH$_2$), 23.53 (α-CH$_2$), 22.78 (α-CH$_2$), 13.12 (α-CH$_3$), 11.37 (α-CH$_3$), 7.48 (s, α-CH$_3$), 6.71 (s, α-CH$_3$).

^{15}N MAS NMR (50.69 MHz): −143.84, −167.03, −170.48, −173.58, −176.60.

Measurements of **3** at different spinning rates confirmed the nature of the smaller, broad signals in the region 130, 160 and 190 as spinning side bands, but the band at 180 ppm and 145 as true peaks (see Supplementary Figure 16/17). Solid-state NMR of the free octaethyl-calix[4]pyrrol ligand confirmed the presence of multiple peaks (more than expected based on symmetry arguments) in the alkyl region originating from packing asymmetry (see Supplementary Figure 18/19).

Mass spectrometric measurement by ESI, LDI, MALDI in DCTB were showing corresponding peaks and isotopic patterns matching with one "cap" - the dianionic ocatethyl-calix[4]-bromidoindinate - but the molecule peak of **3** could not be observed.

The elemental analysis of the isolated crystals of compound **3** consistently gave 8-9% lower carbon values than the calculated values. To determine the source of impurity, the powder XRD of the isolated crystals was measured (Supplementary Figure 10). The pXRD data showed the signals corresponding to complex **3** (matching with the predicted spectrum from the scXRD data) (Supplementary Figure 11). The pXRD spectrum also showed the intensities corresponding to metallic bismuth identified by phase identification from the JCPDS database (Supplementary Figure 12). The contamination of metallic bismuth is the source of low carbon values in the EA analysis. Despite several washing of the crystals, the bismuth impurities could not be removed. The pXRD data is given below.

Calculation of the amount of Bi(metal) in the isolated sample of **3**.

Values for elemental analysis for samples of **3**:

1) C, 29.41; H, 3.37; N, 3.59.
2) C, 29.79; H, 3.33; N, 3.46.
3) C, 30.36; H, 3.58; N, 3.62.

Average of found values **C, 29.85; H, 3.42; N, 3.55**.

The purity of the sample can be calculated individually for each element CHN by the ratio of the CHN-content of the measured values with that in pure **3**·CH_2Cl_2 (C, 36.78; H, 4.14; N, 4.70). It yields a purity of 81.2% based on C; of 82.6% based on H and of 75.5% based on N.

Based on the 81.2% purity of the 30 mg sample, this translates to 24 mg of **3**·CH_2Cl_2 [2383.909 g/mol], which corresponds to 1,007 · 10^{-5} mol, and a yield of **28%**.

Note on magnetic measurements: Solid-state EPR spectroscopy and SQUID measurements revealed trace amounts of paramagnetic species (max. 2%, as quantified by SQUID). These were assigned to side products occurring upon exposure to air, which is unavoidable during the EPR/SQUID sample preparation at our facilities. In any case, it was confirmed that higher paramagnetic spin states should not play a role for compound **3**. This is in line with the DFT-computed triplet state of **3**, which is 125 kJ mol^{-1} above the singlet state.

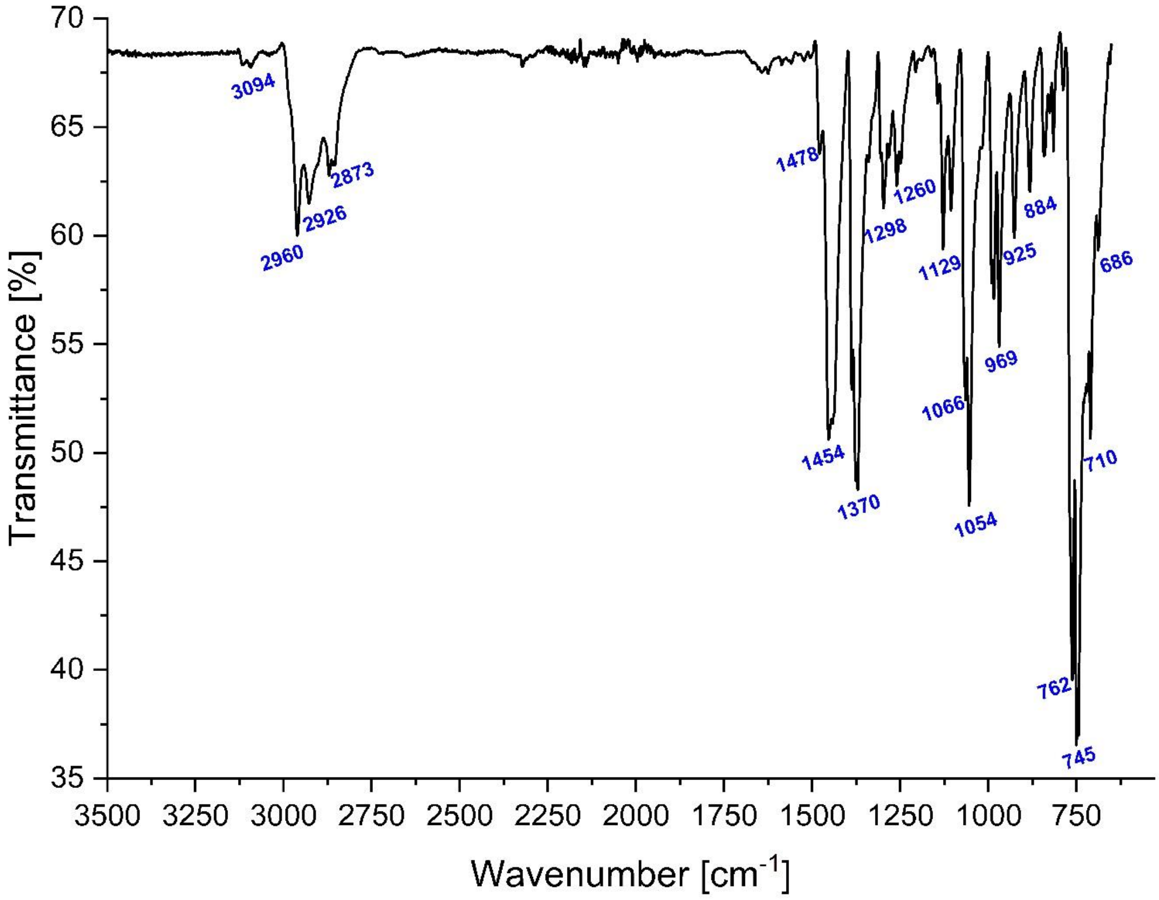

Supplementary Figure 9. IR(ATR) spectrum of complex **3** in solid-state.

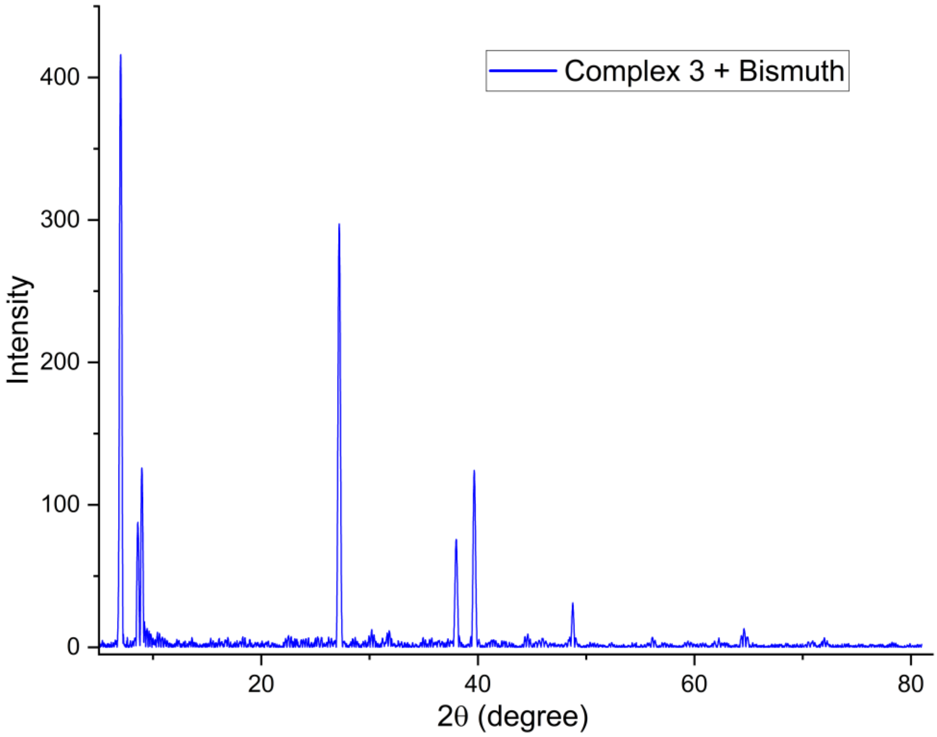

Supplementary Figure 10. The origin plot of PXRD spectrum of the crystals of complex **3**.

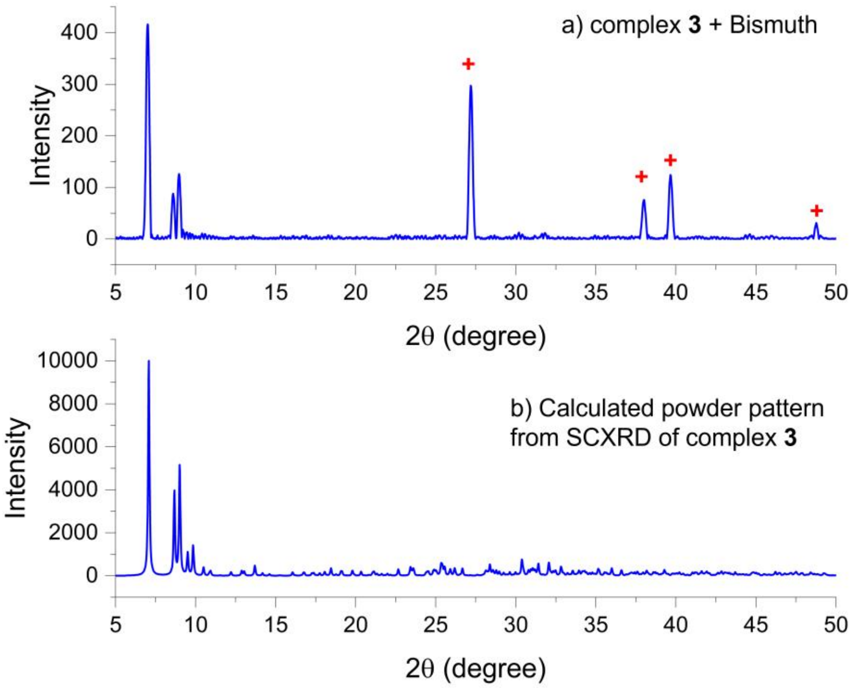

Supplementary Figure 11. a) The pXRD spectrum of crystals of complex **3**. b) Using Mercury software, the calculated powder pattern from the scXRD data of complex **3**. The stacked spectra showed the matching between the calculated and the experimental spectrum. + = impurity of metallic bismuth.

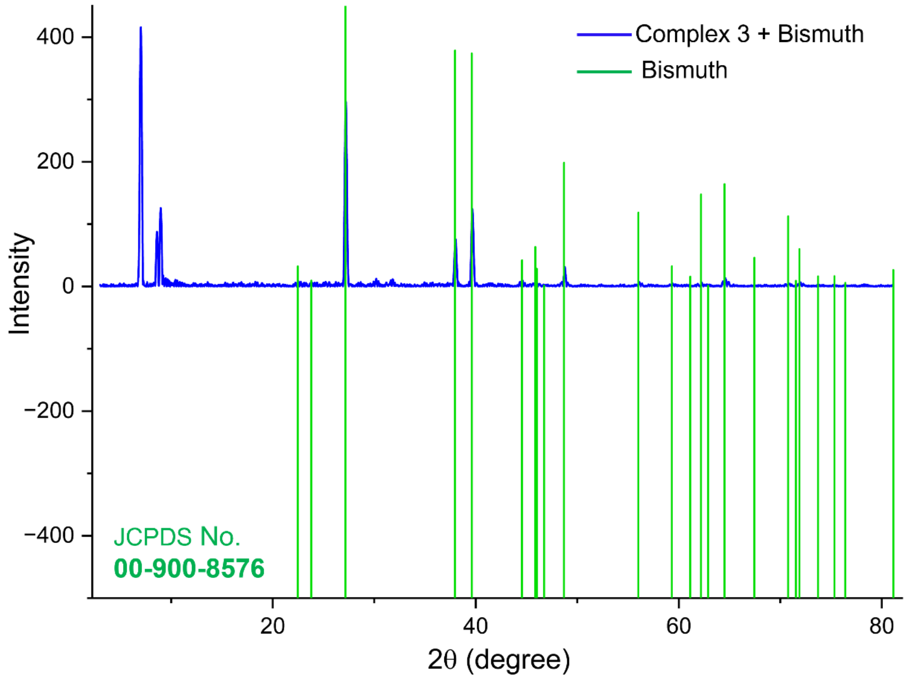

Supplementary Figure 12. The PXRD spectrum of crystals of complex **3** (above, blue spectrum), the impurity identified as bismuth metal (JCPDS number 00-900-8576)[5] by using search and match option with QualX[4] software and COD database.[6]

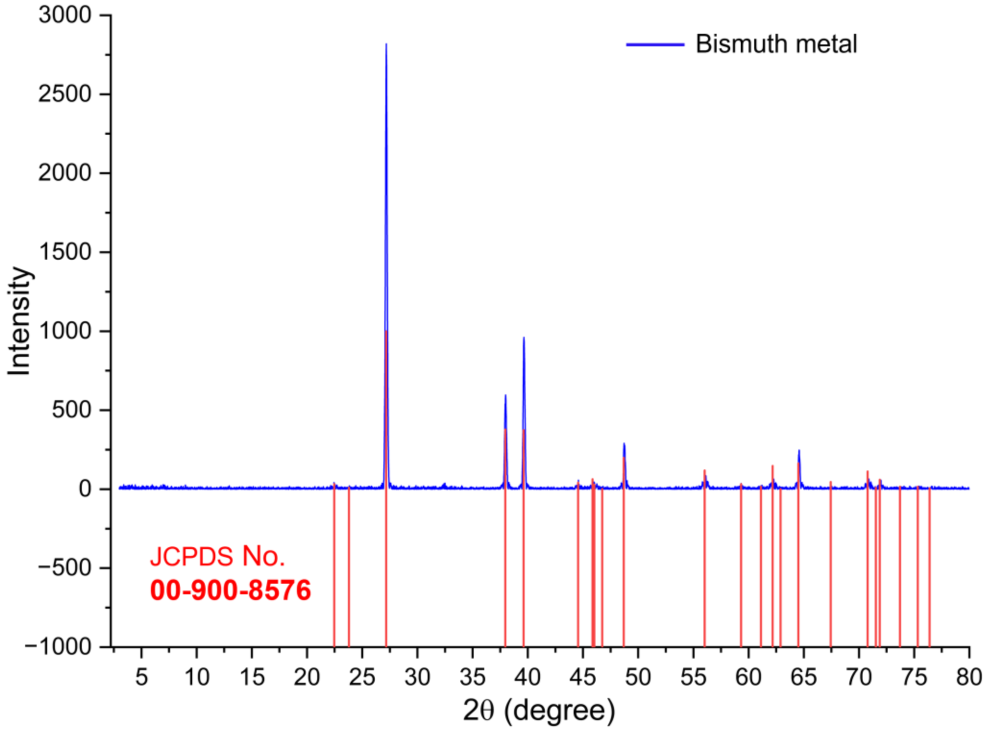

Supplementary Figure 13. The PXRD spectrum of powder deposited during the formation of complex **3**, the isolated powder was identified as bismuth metal (JCPDS number 00-900-8576)[5] by using search and match option with QualX[4] software and COD database.[6]

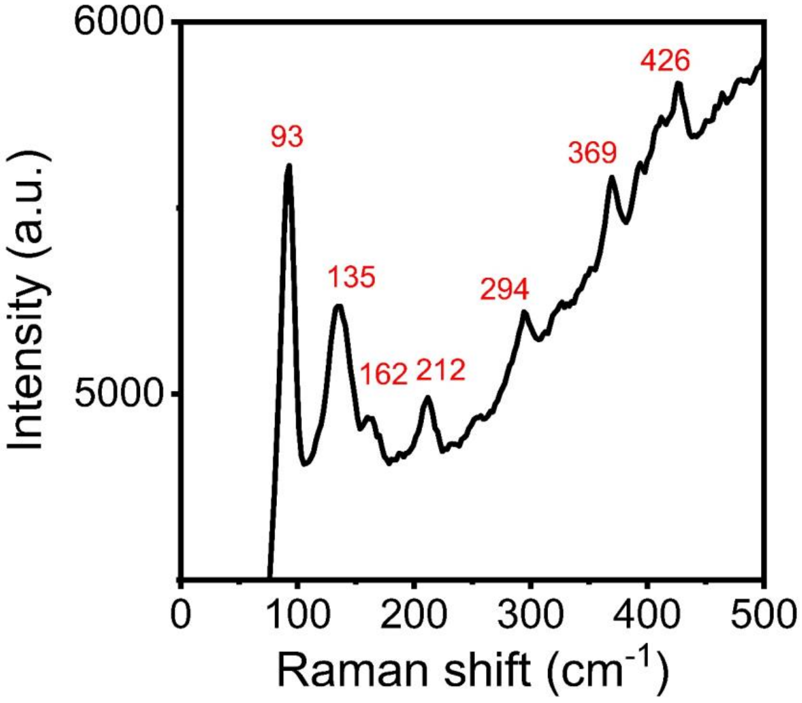

Supplementary Figure 14. Raman spectrum of solid sample of **3** (for assignment of bands, see section S3.3). (for assignment of bands, see section S3.4)

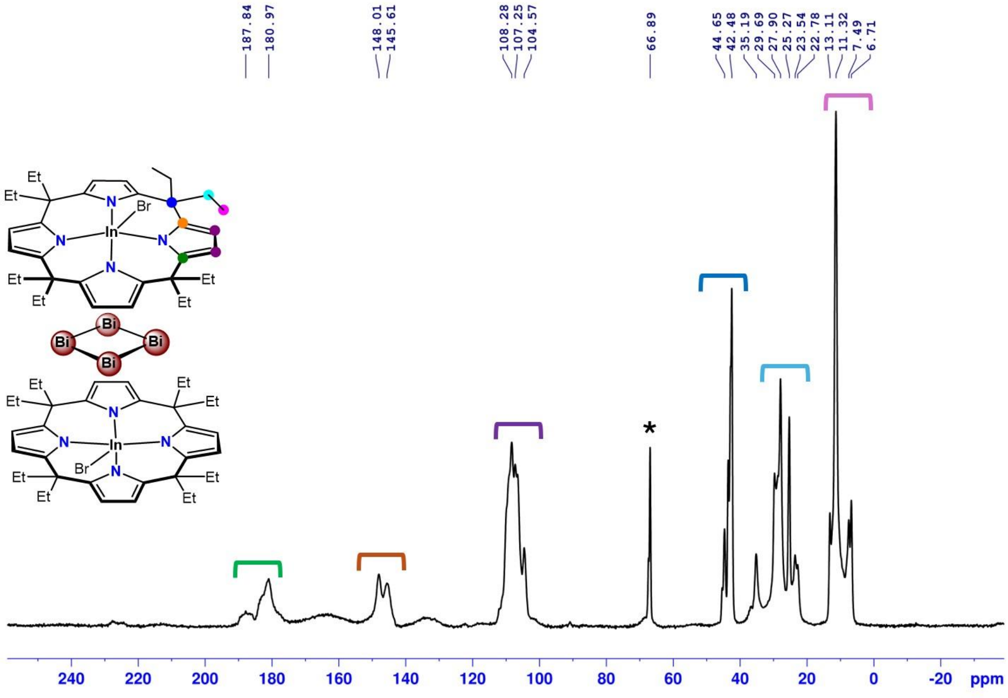

Supplementary Figure 15. ^{13}C CP-MAS NMR spectrum of **3; *** = CH_2Cl_2.

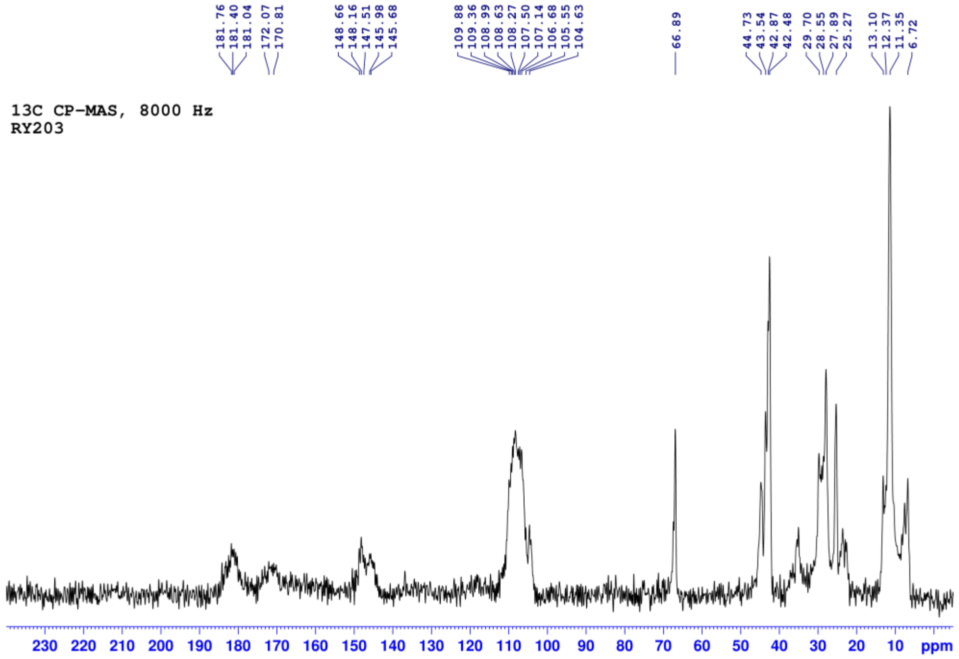

Supplementary Figure 16. ^{13}C CP-MAS NMR of **3** at 8000 Hz spinning rate.

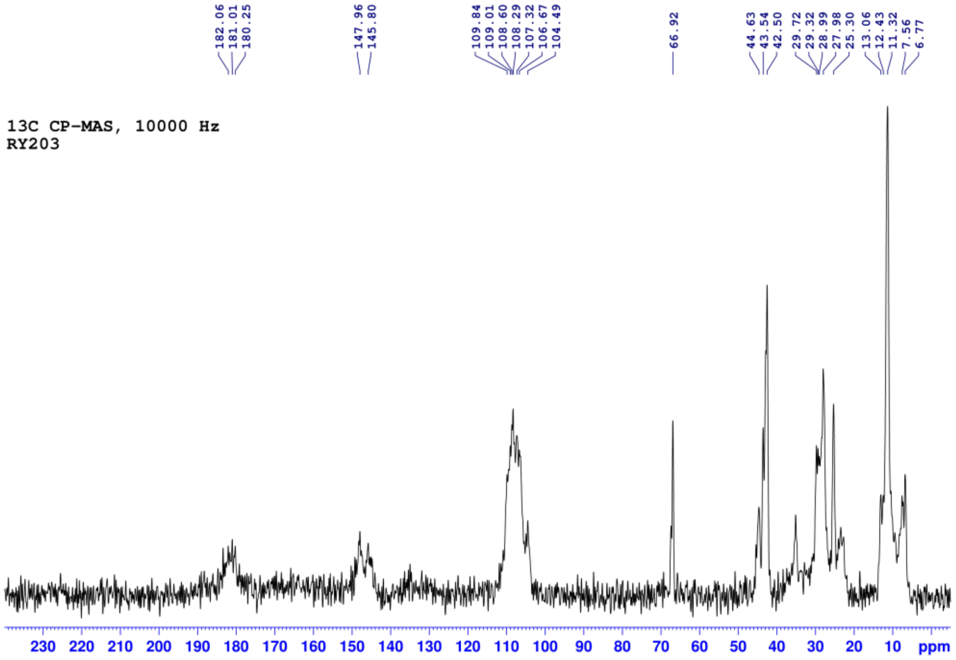

Supplementary Figure 17. ^{13}C CP-MAS NMR of **3** at 10000 Hz spinning rate.

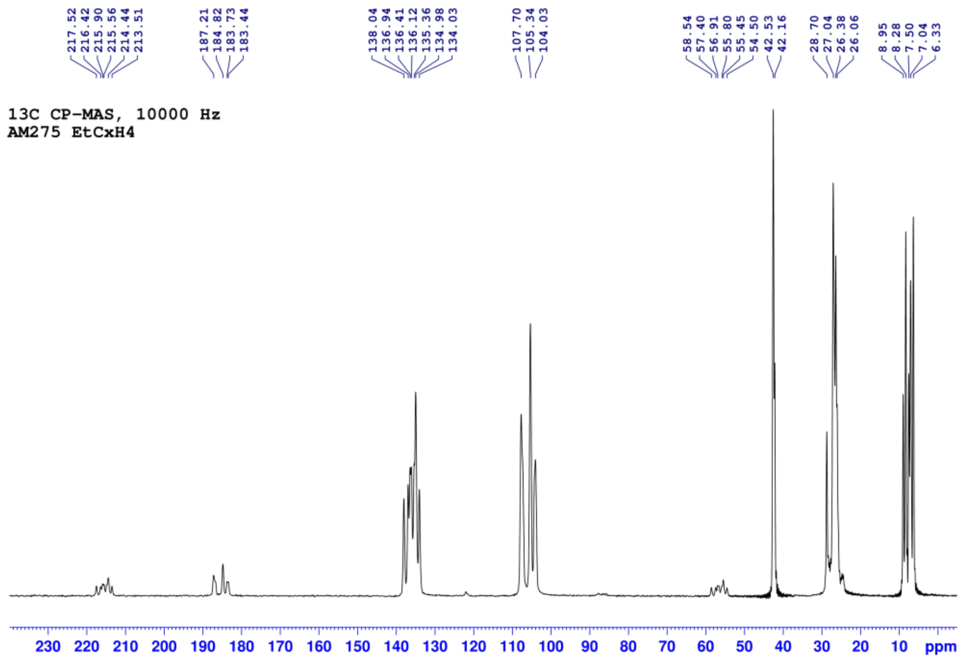

Supplementary Figure 18. ^{13}C CP-MAS NMR of **EtCx[4]pyrrole** at 10000 Hz spinning rate.

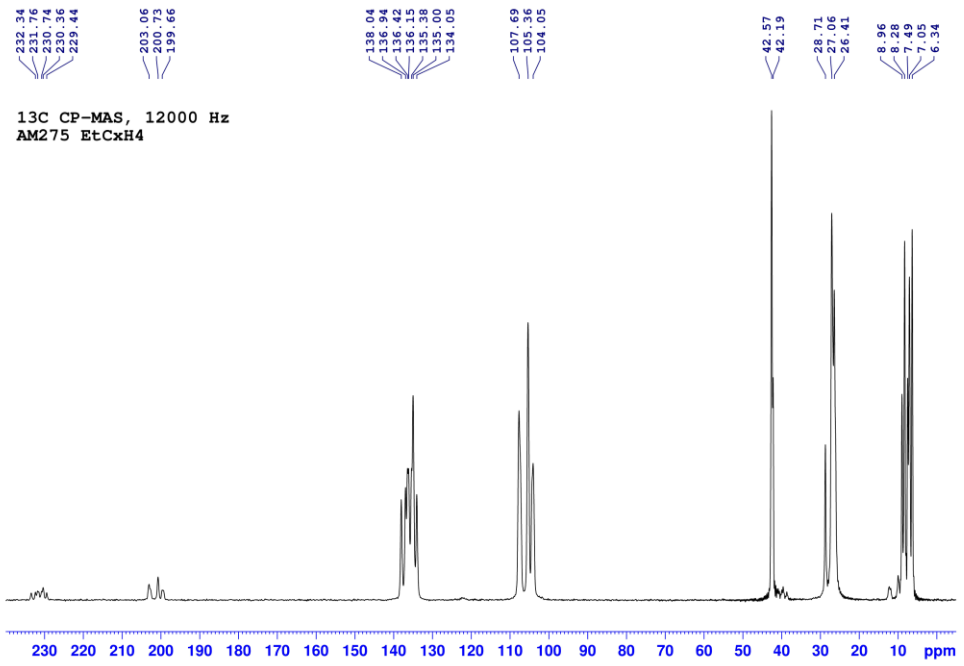

Supplementary Figure 19. ^{13}C CP-MAS NMR of **EtCx[4]pyrrole** at 12000 Hz spinning rate.

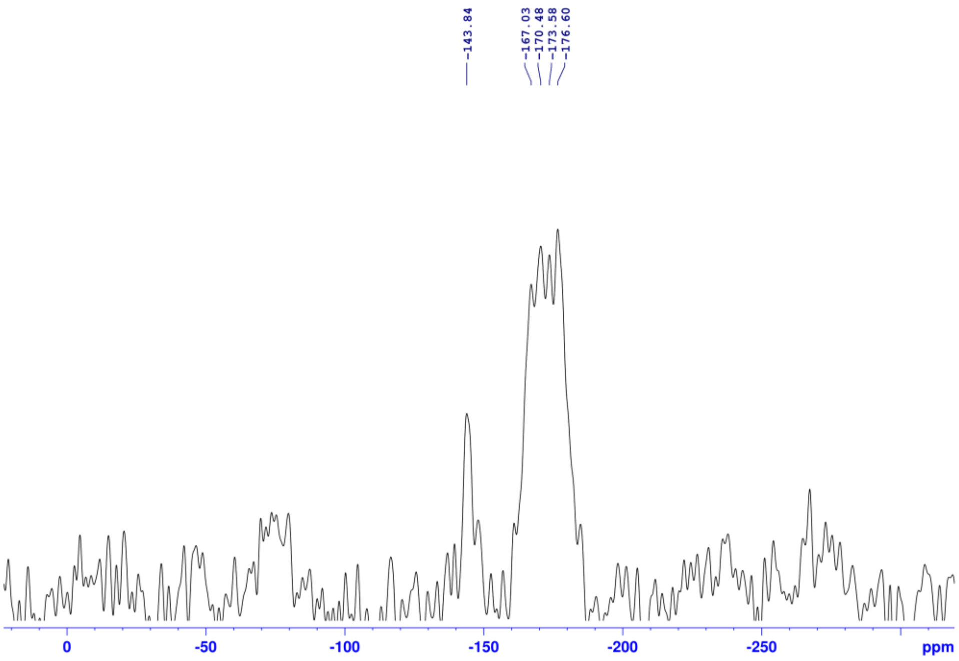

Supplementary Figure 20. ^{15}N MAS NMR of **3**.

Compound 4

Workup of the filtrate: all the solvents were removed from the filtrate, and the residue was dissolved in toluene 10 mL and allowed to stand at room temperature for 16 h and then at -40 °C for 6 hours. The reaction mixture was filtered (PPh_4InBr_4 was identified by SCXRD as one of the components of the residue at this stage) and all the volatiles were removed from the filtrate. The orange solid was washed with pentane and dried *in vacuo*. The crude product was further purified by recrystallization by standing a saturated solution of benzene at room temperature-

Yield of **4**: 70 mg (62%, 0.095 mmol). **Elem. Anal.** for **[$C_{36}H_{48}N_4BrIn$]** calculated: C, 59.11; H, 6.61; N, 7.66. Found: C, 59.07; H, 6.58; N, 7.83.

LIFDI Mass Spectrometery in thf: **[$C_{36}H_{48}N_4BrIn$]** calculated: 730.21012 m/z found: 730.1912 m/z.

^{1}H NMR (600 MHz, toluene-d$_8$, 298 K): δ = 6.34 (br, $\Delta\nu_{1/2} \approx$ 6.9 Hz, 8H, C*H*, β-C), 2.50 (br, $\Delta\nu_{1/2} \approx$ 30 Hz, 8H C*H*$_2$-ethyl), 1.99 (br, $\Delta\nu_{1/2} \approx$ 50 Hz, 8H, C*H*$_2$-ethyl), 0.89 (br, $\Delta\nu_{1/2} \approx$ 30 Hz, 12H C*H*$_3$-ethyl), 0.78 (t, $^3J_{HH}$ = 7.2 Hz, 12H, C*H*$_3$-ethyl).

^{1}H NMR (600 MHz, toluene-d_8, **343** K): δ = 6.34 (s, 8H, C*H*, β-C), 2.46 (q, $^{3}J_{HH}$ = 7.3 Hz, 8H), 2.02 (q, $^{3}J_{HH}$ = 7.3 Hz, 8H, CH_2-ethyl), 0.91 (t, $^{3}J_{HH}$ = 7.3 Hz, 12H, CH_3-ethyl), 0.76 (t, $^{3}J_{HH}$ = 7.2 Hz, 12H, CH_3-ethyl).

^{13}C{^{1}H} NMR spectrum of compound **2** could not be observed due to the delocalization of the cyclopropyl ring of **2**.

*Note: The ^{1}H NMR spectrum of compound **4** shows a symmetric species, which is unprecedented for a Δ-form of the calix[4]pyrrole ligand. This behavior has been fully investigated for Δ-calix[4]pyrrolato-indium-halides. Compound **4** is a by-product but can be directly synthesized by other routes. The complete study of VT-NMR, EPR, VT-UV-vis spectroscopy of compound **4** along with other Δ-calix[4]pyrrolato-indium-halides will be published in a separate manuscript. Here we only report the ^{1}H NMR, elemental analysis, LIDFI mass spectrometery, SCXRD of compound **4** to establish the identity and purity.*

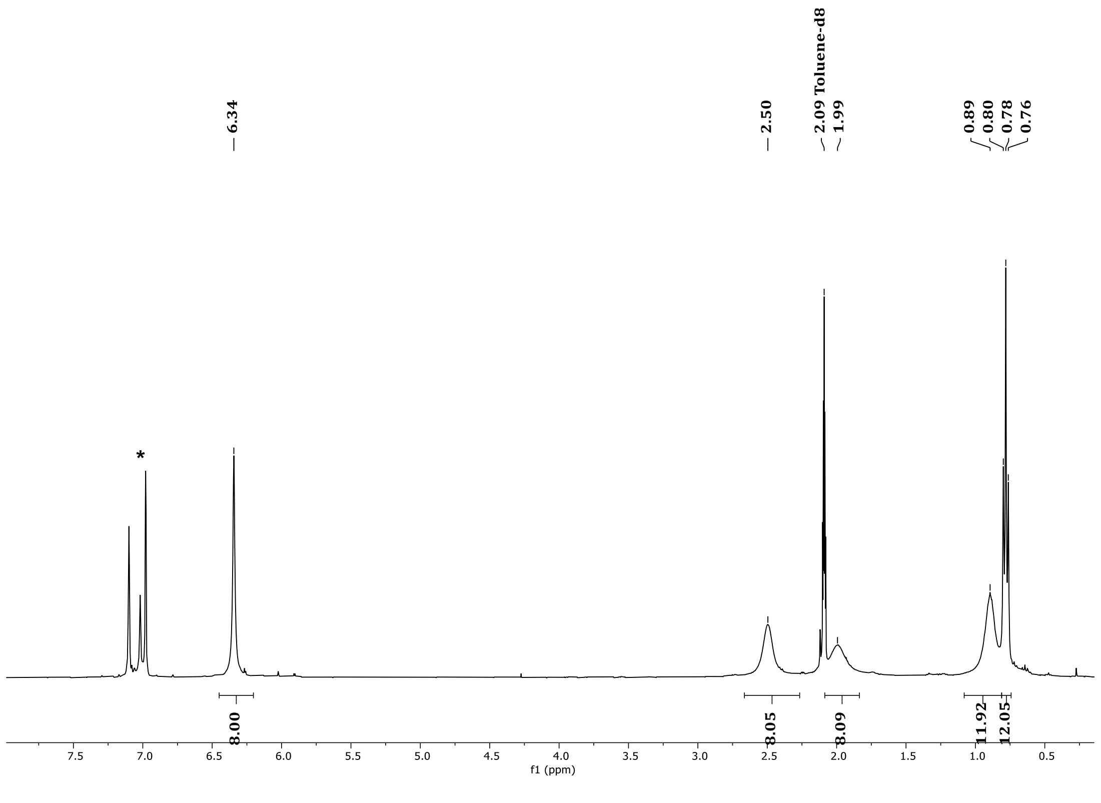

Supplementary Figure 21. ^{1}H NMR (600 MHz, 298 K, toluene-d_8) spectrum of compound **4**. * = residual protio solvent from toluene-d_8.

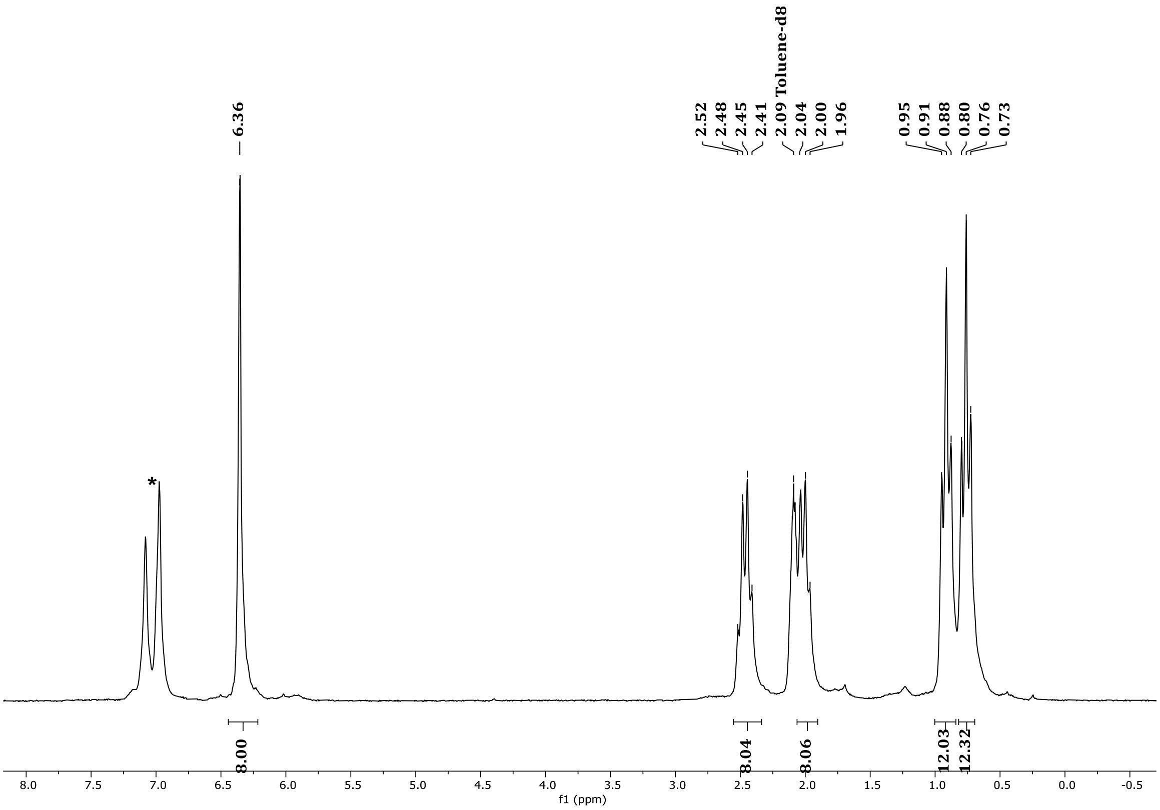

Supplementary Figure 22. ^{1}H NMR (600 MHz, **343 K**, toluene-d_8) spectrum of compound **4**. * = residual protio solvent from toluene-d_8.

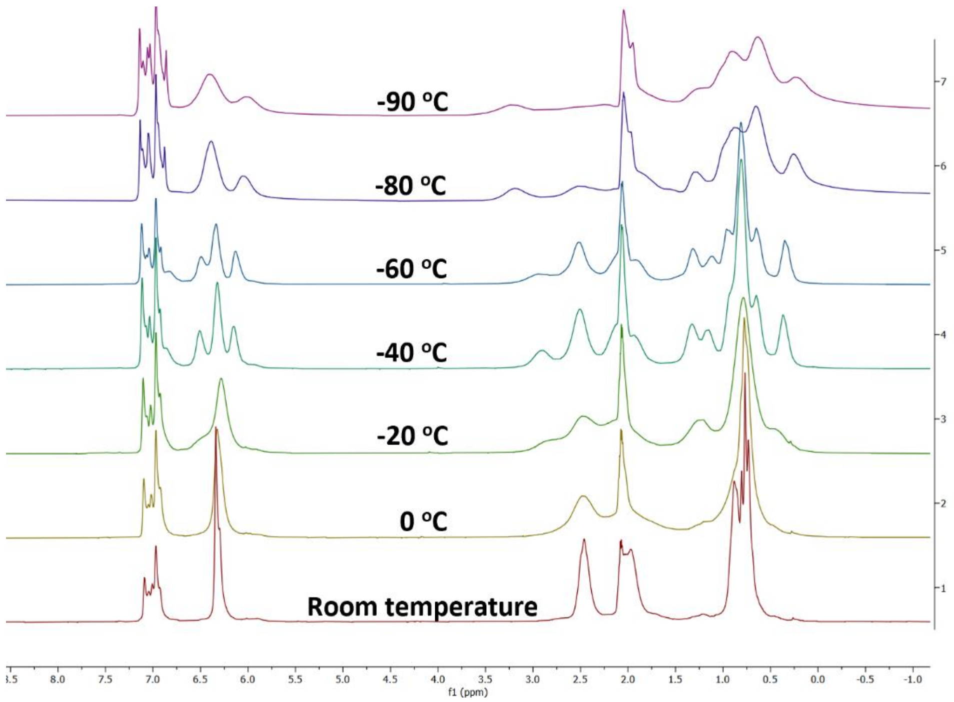

Supplementary Figure 23. VT ^{1}H NMR (200 MHz, 298 to 208 K, toluene-d_8) spectrum of compound **4**.

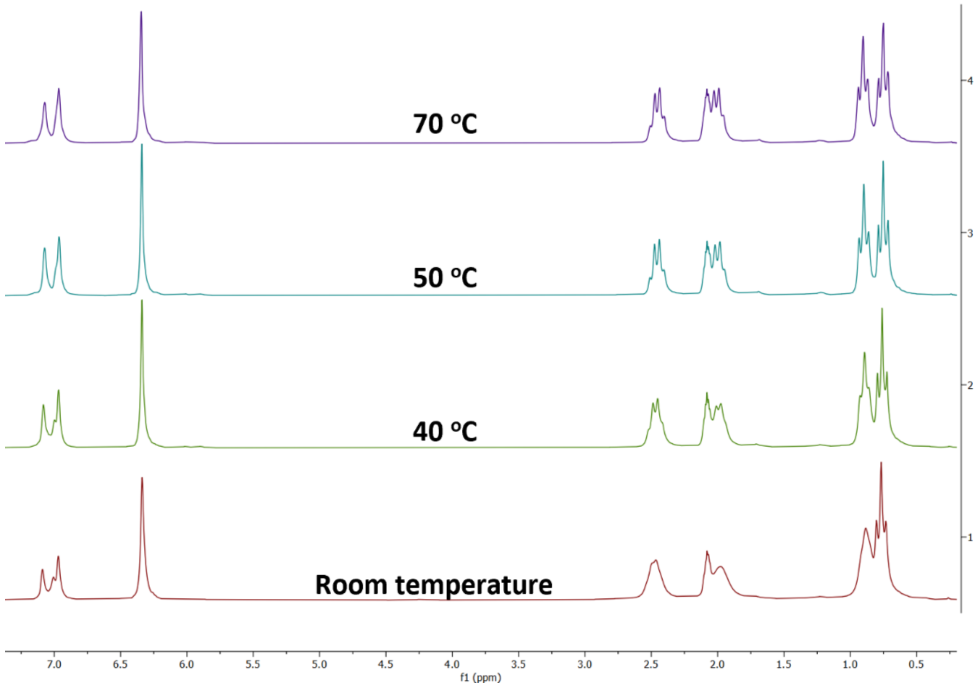

Supplementary Figure 24. VT ^{1}H NMR (200 MHz, 298 to 343 K, toluene-d_8) spectrum of compound **4**.

1.2.4. Second route for the synthesis of 3 and 4 for mechanism studies

Synthesis of tetraphenylphosphonium *meso*-octaethylcalix[4]pyrrolato indinate-(pyridine) (5)

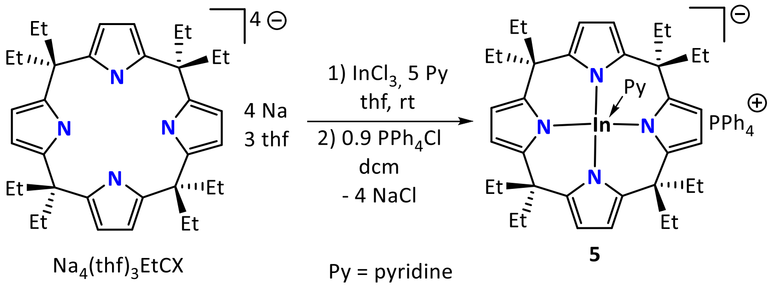

$Na_4(thf)_3EtCx$[7] (300 mg, 0.354 mmol) was dissolved in 10 mL thf and pyridine (142 µL, 1.77 mmol) was added to the solution. Then, a thf (15 mL) solution of $InCl_3$ (78 mg, 0.354 mmol) was added to the solution dropwise. The reaction mixture was stirred at room temperature for 6 hours, and all the volatiles were removed under vacuum. The solid obtained was suspended in 20 mL dcm, and PPh_4Cl (199 mg, 0.319 mmol) was added as a solid to the

suspension. The reaction mixture was further stirred for 16 hours, and the solvent was removed *in-vacuo* to ensure complete precipitation of NaCl. The reaction mixture was suspended in a mixture of solvent dcm:pentane (15 mL: 5mL), stirred for 3 hours and filtered over celite padded filter frit. The filtrate was dried, and the last step was repeated for complete elimination of NaCl. The solid obtained was dried for 24 hours at high vacuum. *Note*: *Even after prolonged drying, a slight excess of pyridine was observed. The ^{1}H NMR showed that there are 1.2 to 1.5 equivalents of pyridine per In-atom.*

Yield of **5**: 330 mg (87%, 0.309 mmol). **Elem. Anal.** for **5** was not be attempted due to the presence of slightly excess pyridine, variable from 1.5 equivalent to 1.2 equivalent per In-atom.

^{1}H NMR (600 MHz, CD_2Cl_2): δ = 7.89–7.84 (m, 4H, PPh_4^+), 7.73–7.67 (m, 8H, PPh_4^+), 7.59-7.55 (m, 10H, PPh_4^+ and *p-H*-pyridine overlapped), 7.17 (br, $\Delta\nu_{1/2} \approx$ 15 Hz, 2H, *o-H*-pyridine), 7.08–7.03 (m, 2H, *m-H*-pyridine), 5.92 (s, 8H, C*H*, β-C), 1.75 (q, *J* = 7.2 Hz, 16H, CH_2-ethyl), 0.54 (t, *J* = 7.2 Hz, 24H, CH_3-ethyl).

^{13}C NMR (151 MHz, CD_2Cl_2): δ = 149.3 (*o-C*-pyridine), 144.5 (C_q, C_q-pyrrole), 138.45, (*p-C*-pyridine) 136.18 (d, PPh_4^+, $^{4}J_{CP}$ = 3.1 Hz), 134.74 (d, PPh_4^+, $^{2}J_{CP}$ = 10.3 Hz), 131.01 (d, PPh_4^+, $^{3}J_{CP}$ = 12.9 Hz), 124.0 (*m-C*-pyridine), 117.82 (d, PPh_4^+, $^{1}J_{CP}$ = 89.6 Hz), 104.21 (*C*H, β-C), 46.67 (C_q, α-C), 38.29 (*C*H_2-ethyl), 10.27 (*C*H_3-ethyl).

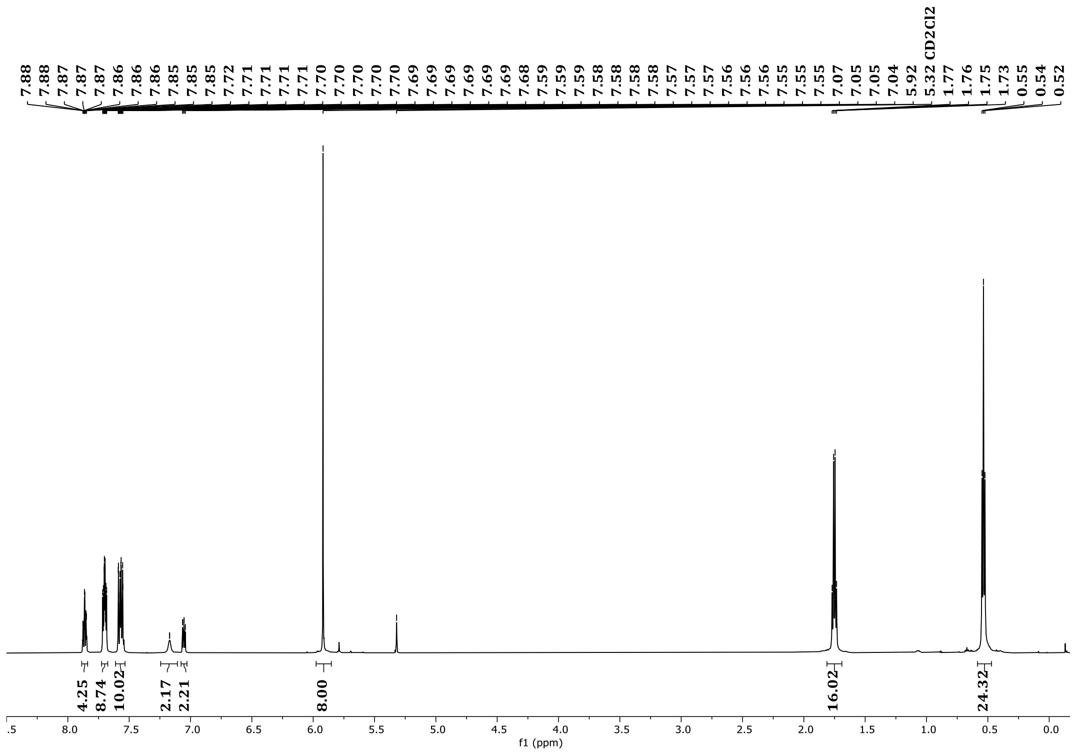

Supplementary Figure 25. ^1H NMR (600 MHz, 298 K, CD_2Cl_2) spectrum of compound **5**.

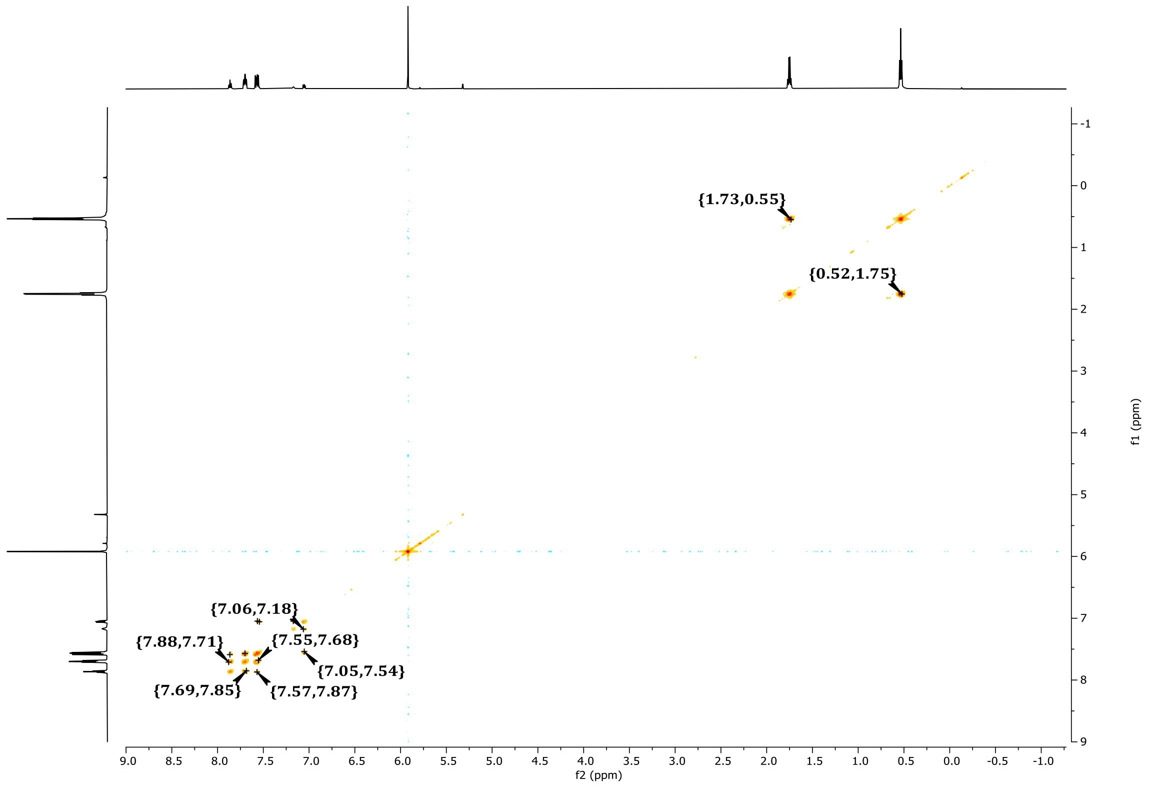

Supplementary Figure 26. ^1H-^1H COSY NMR (600 MHz, 298 K, CD_2Cl_2) spectrum of compound **5**.

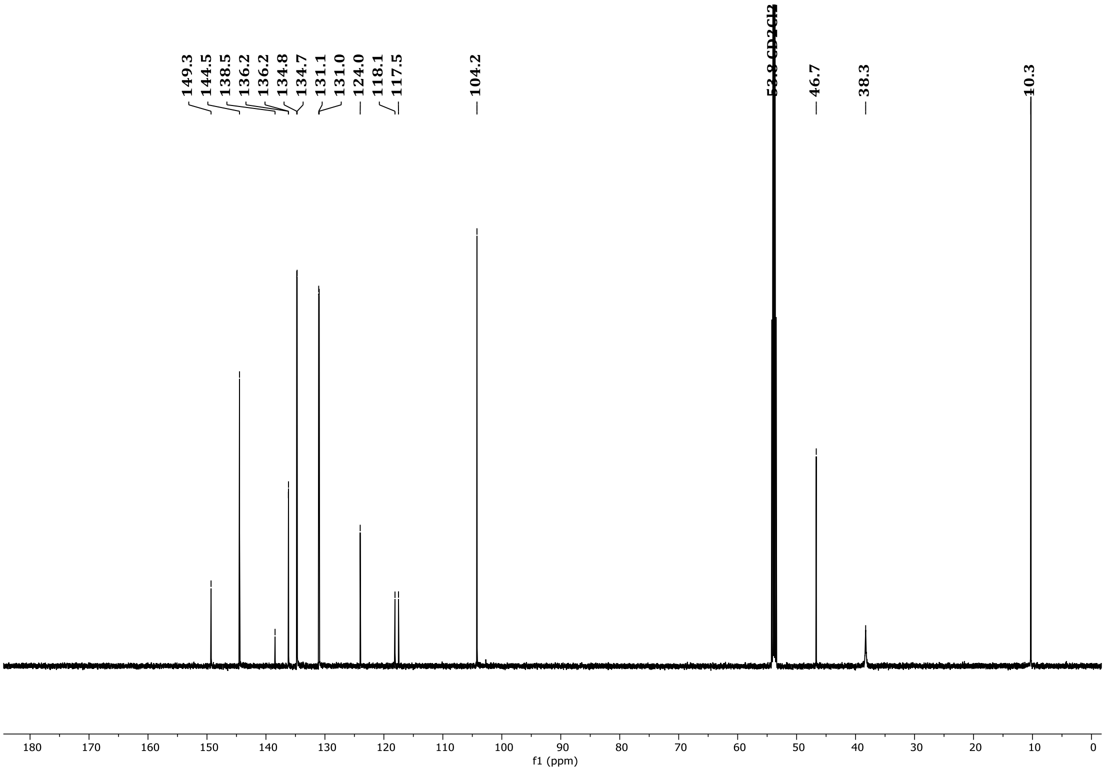

Supplementary Figure 27. $^{13}C\{^{1}H\}$ NMR (151 MHz, 298 K, CD_2Cl_2) spectrum of compound **5**.

1.2.5. In-situ generation of complex 6 and 7 and reaction with $BiCl_3$

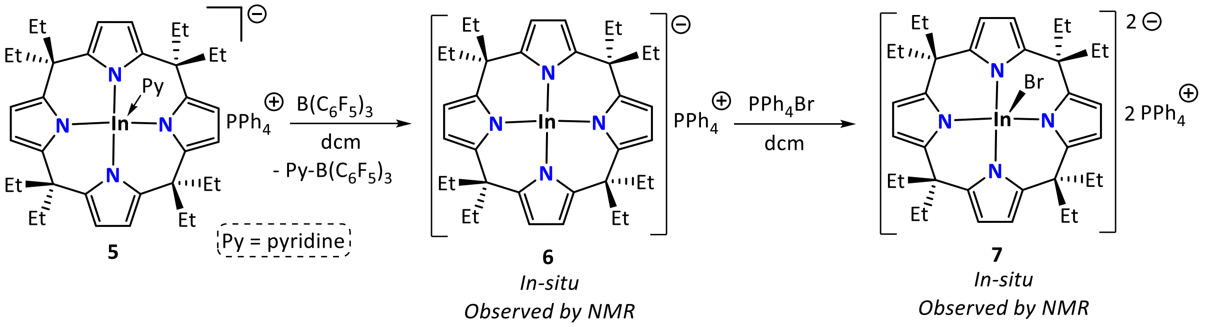

For the synthesis of complex **7**, complex **6** was synthesized by removing pyridine. Complex **5** (100 mg, 0.093 mmol) was dissolved in 5 mL of dcm and $B(C_6F_5)_3$ (52 mg, 0.102 mmol) was added, and stirred for 5 minutes (the ^{1}H NMR spectrum of the same reaction at an NMR scale (10 mg of **5**) showed formation of a symmetric species assigned as **6** and pyridine-$B(C_6F_5)_3$ (Supplementary Figure 28)). PPh_4Br (39 mg, 0,093 mmol) was added to the reaction mixture and stirred for 10 minutes at room temperature. The NMR spectrum at this stage showed the formation of complex **7** (Supplementary Figure 30).

In both cases, after 5 minutes aliquot was taken for HRMS measurement to check the formation of **6** and **7.**

HRMS (ESI neg.) m/z calculated for **6** $[M]^-$: $[C_{36}H_{48}InN_4]$: 651.2918, found: 651.2935.

HRMS (ESI neg.) m/z calculated for **7** $[M+H]^-$: $[C_{36}H_{49}BrInN_4]$: 731.2179 , found: 731.2195.

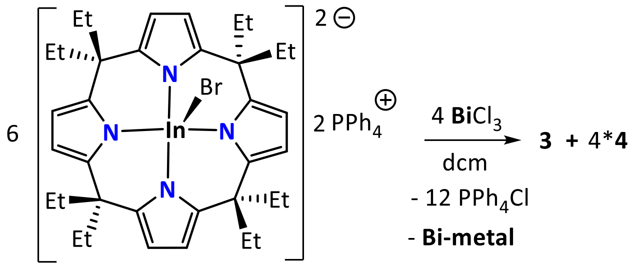

7
In-situ
Observed by NMR

Purification and isolation of complexes **6** and **7** were not attempted, as these complexes were thermally unstable and decomposed during the work-up. Hence, the synthesis of complex **3** and **4** from this route was done by directly using the reaction solution with $BiCl_3$ (22 mg, 0.070 mmol). As soon as the $BiCl_3$ was added to the reaction mixture, it turned dark wine red, indicating the formation of **4** and reduction of Bi(III). The reaction mixture was stirred for 10 minutes and then left standing at room temperature overnight. The crystals of complex **3** deposited in the reaction flask along with the formation of Bi-metal powder. The identity of complex **3** was confirmed by scXRD. The workup of the reaction showed lower yields of **3** and **4** as compared to the previous procedure (Section 1.2.3) and hence it was not followed further.

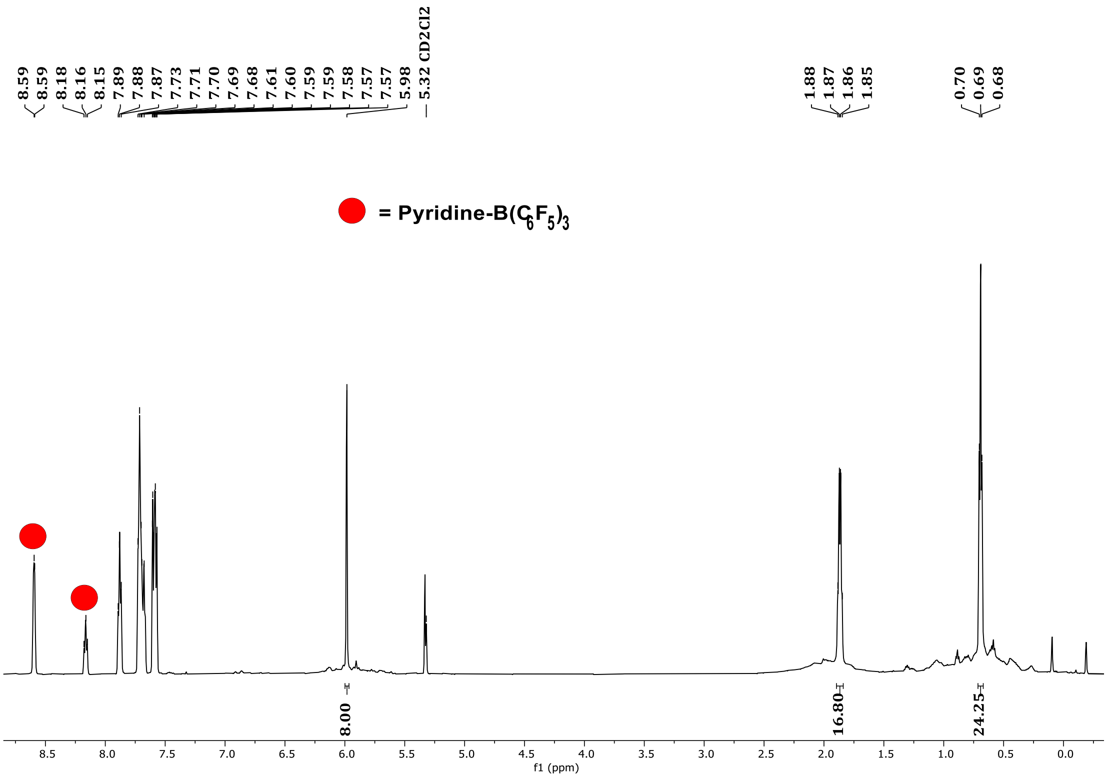

Supplementary Figure 28. ^{1}H NMR (600 MHz, 298 K, CD_2Cl_2) spectrum of reaction between compound **5** and $B(C_6F_5)_3$ after 10 minutes. The spectrum shows compound **6** along with pyridine-$B(C_6F_5)_3$.

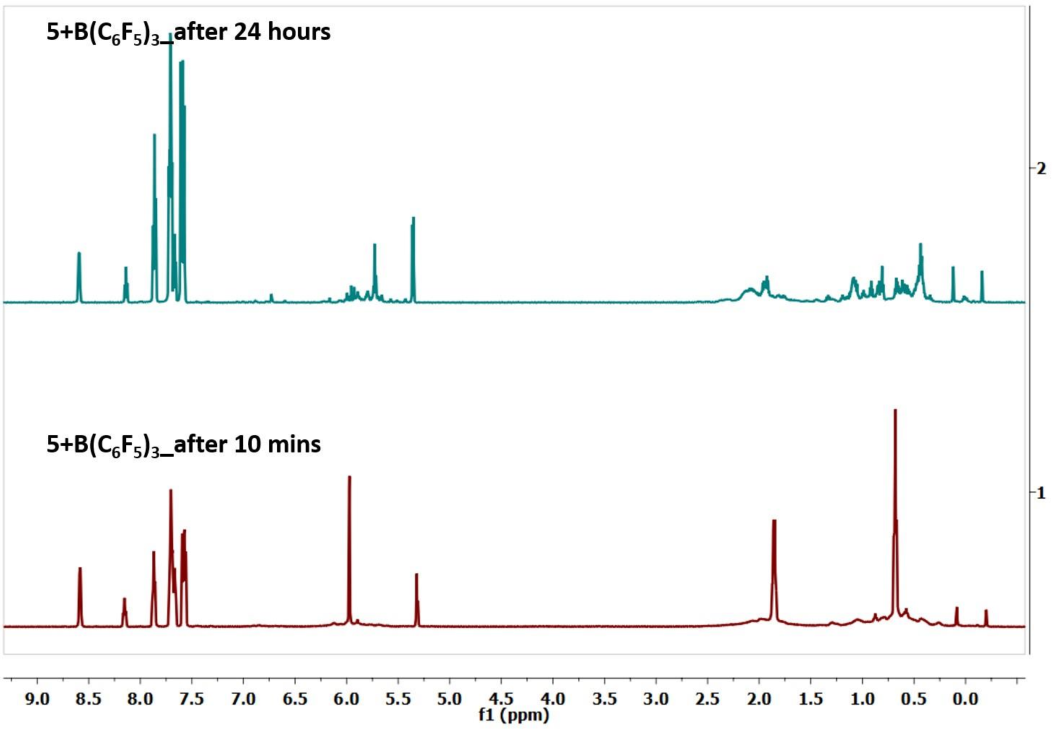

Supplementary Figure 29. Decomposition of **6** at room temperature**.**

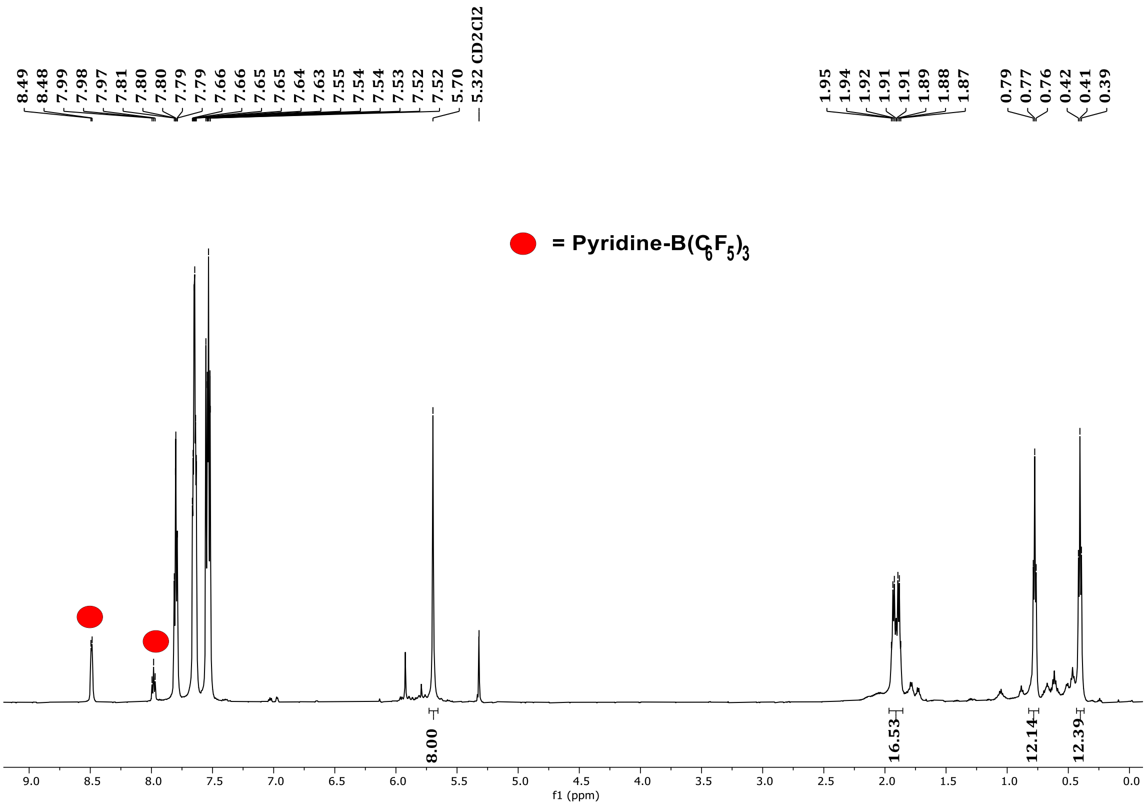

Supplementary Figure 30. ^{1}H NMR (600 MHz, 298 K, CD_2Cl_2) spectrum of reaction between compound **6** (*in-situ formed*) and PPh_4Br after 15 minutes. The spectrum showing compound **7** along with pyridine-$B(C_6F_5)_3$.

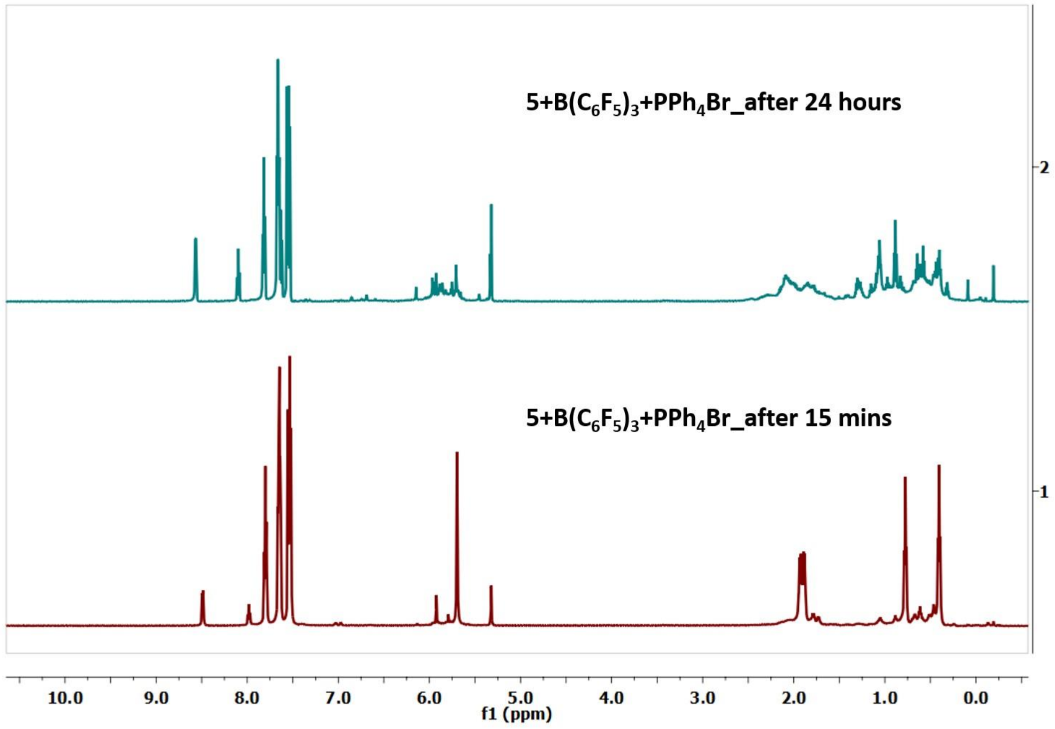

Supplementary Figure 31. Decomposition of **7** at room temperature**.**

1.2.6. Cyclic Voltammetry Experiment

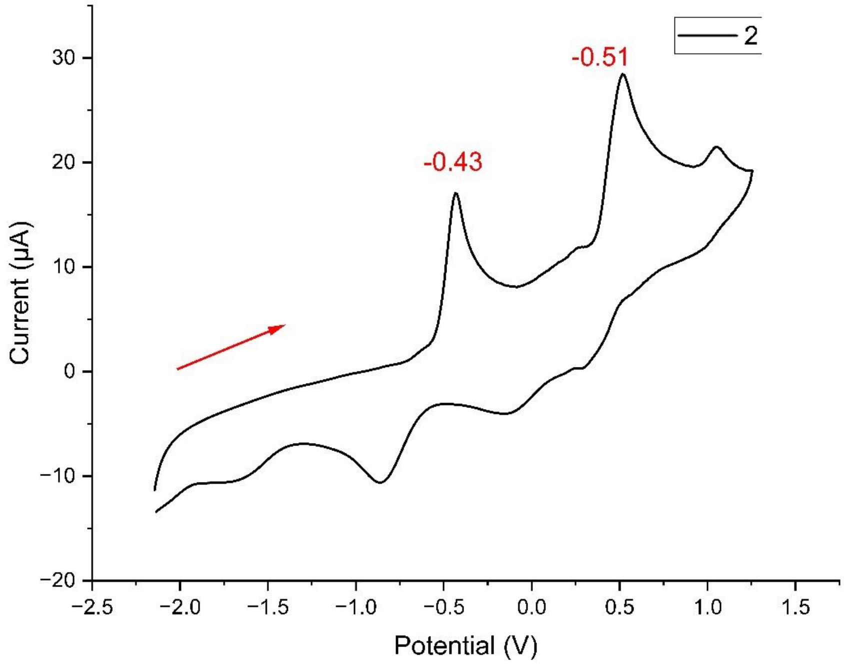

Supplementary Figure 32. CV measurement of **2** in dichloromethane with *n*Bu_4NPF_6 at 100 mV/s.

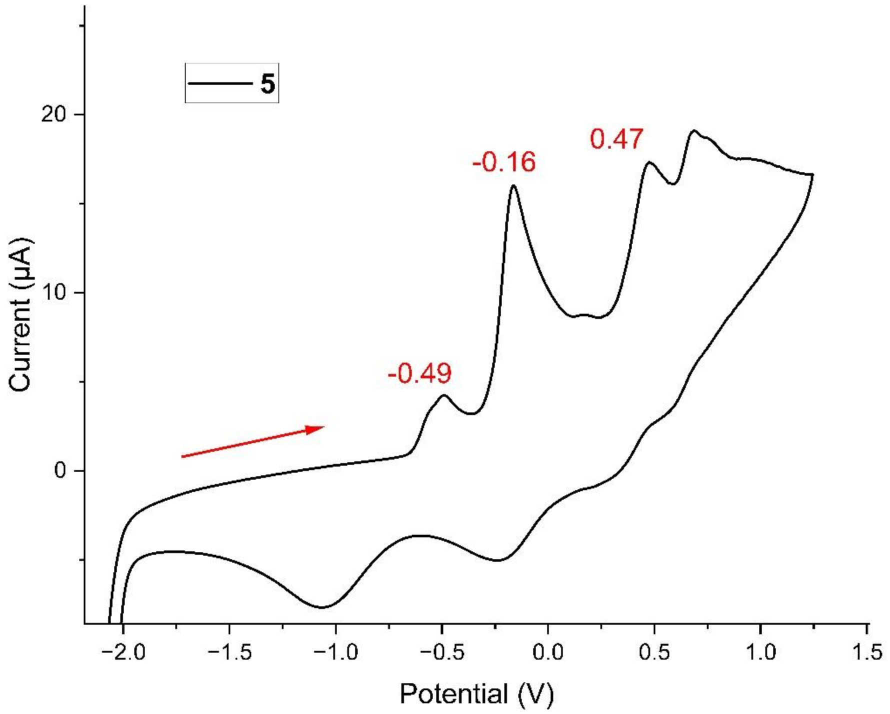

Supplementary Figure 33. CV measurement of **5** in dichloromethane with *n*Bu_4NPF_6 at 100 mV/s.

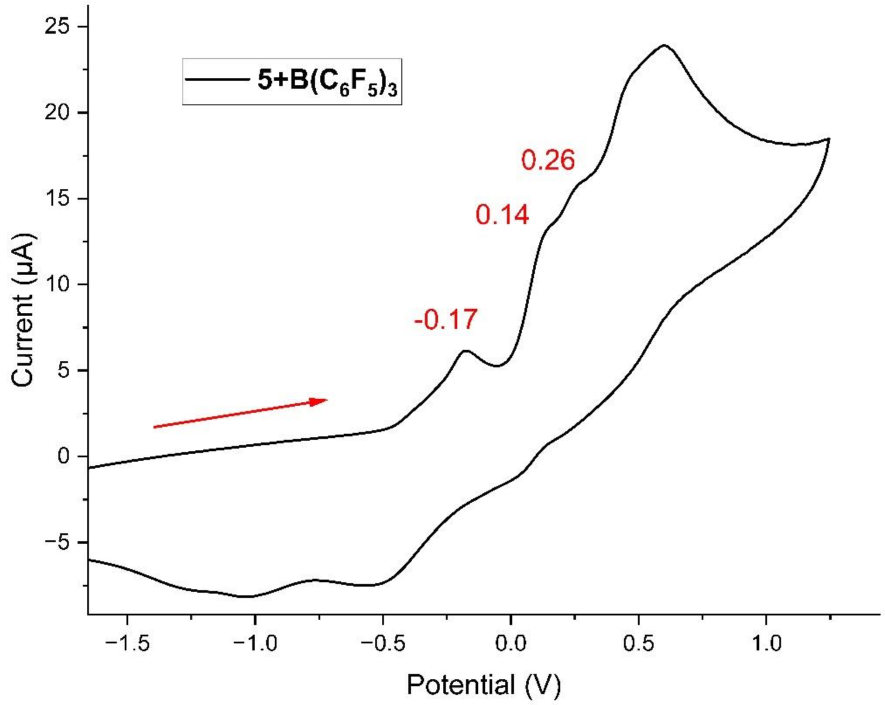

Supplementary Figure 34. CV measurement of **5** in presence of equivalent amount of **$B(C_6F_5)_3$** in dichloromethane with *n*Bu_4NPF_6 at 100 mV/s.

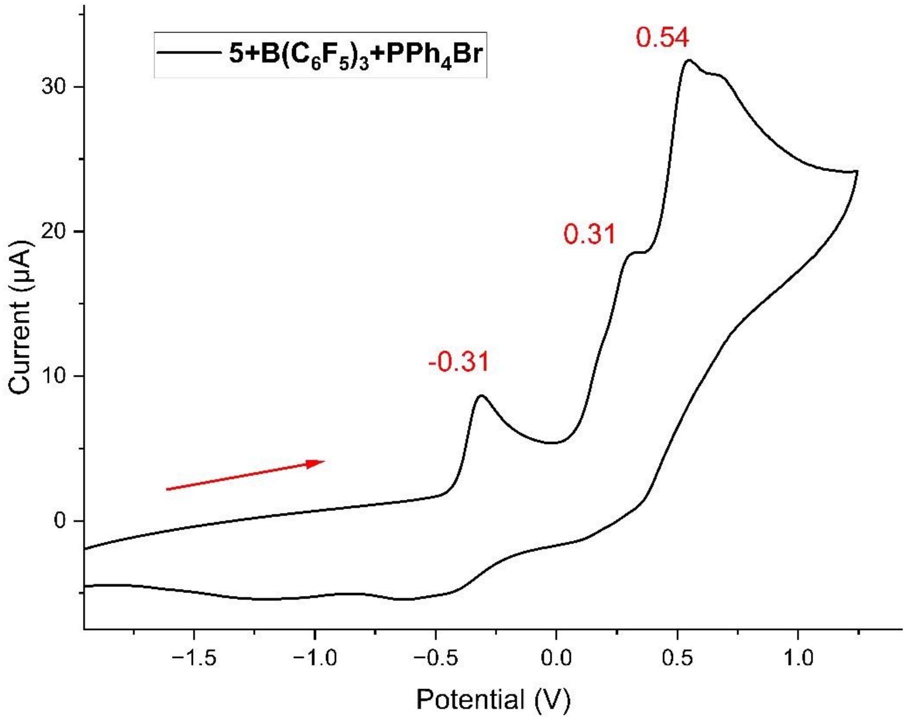

Supplementary Figure 35. CV measurement of **5** in presence of equivalent amount of **$B(C_6F_5)_3$** and **PPh_4Br** in dichloromethane with *n*Bu_4NPF_6 at 100 mV/s.

1.2.7. Reduction of 3 with KC_8 under ball milling condition

3 + KC_8 $\xrightarrow[\text{180 mins, 30 Hz}]{\text{ball milling}}$ **Bi** metal + amorphous potassium-salts of calix[4]pyrrolates

In a 1.5 ml stainless steel ball mill jar, **3** (10 mg, 4.349 µmol) and **KC_8** (2.35 mg, 17.3 µmol) was added and milled for 180 mins at 30 Hz speed. The residue was washed with THF and pXRD was taken which confirms the presence of only Bi metal. pXRD of the sample before and after ball milling is shown below.

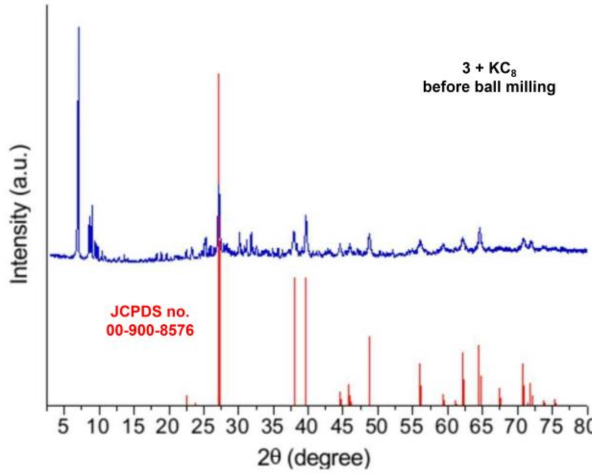

Supplementary Figure 36. pXRD spectrum complex **3** with KC_8 before ball milling. As shown above Bi metal powder is present already in sample of **3**.

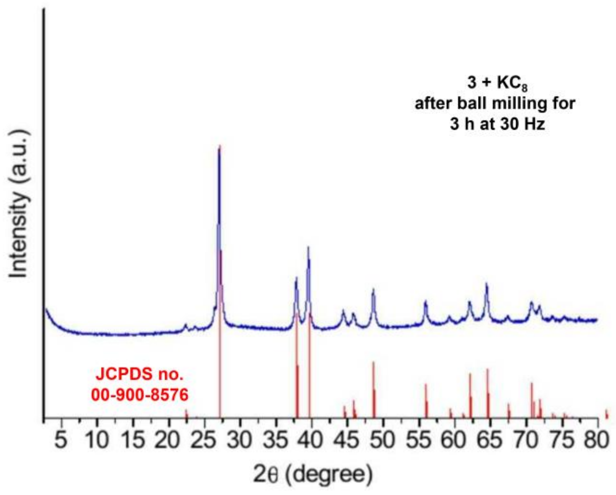

Supplementary Figure 37. pXRD spectrum complex **3** with KC_8 after ball milling. Only peaks of polycrystalline Bi powder are present.

1.2.8. Synthesis of 8^E

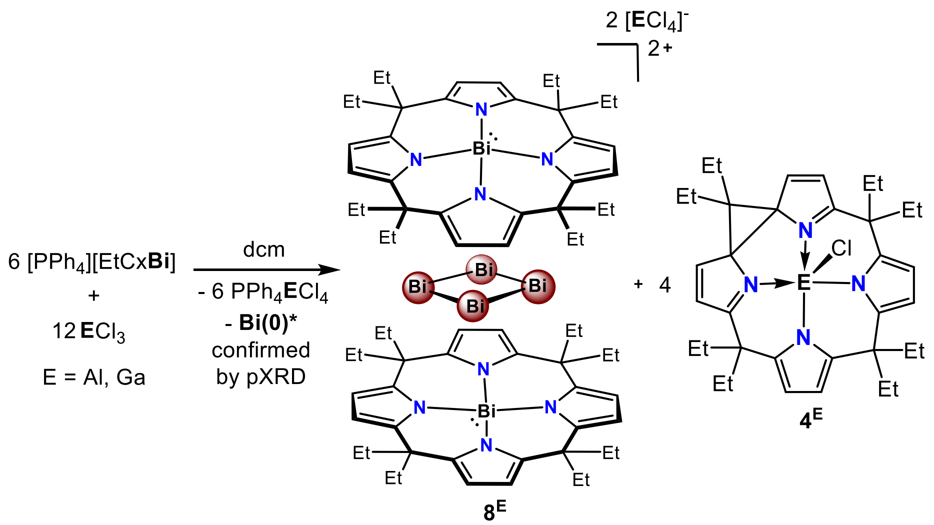

2 ml DCM was added in a vial containing **2** (200 mg, 0.184 mmol) and **ECl_3** (E = Al, 48 mg, 0.368 mmol and E = Ga, 64 mg, 0.092 mmol). Immediately the solid was dissolved with deep red color solution appears. The vial was kept at room temperature for 3 days. After that dark black color crystalline solid with black powder precipitated which was washed with DCM, THF, CH_3CN, Hexane.

Yield of **8^E**: E = Al: 25mg (24 %), E= Ga: 33 mg (31%).

HRMS (ESI pos.) m/z calculated for **8^{Ga}** $[M-Cl]^-$: $[C_{72}H_{96}Bi_6N_8Cl]$: 2361.6269, found: 2361.6263.

Elemental analysis: calcd for **8^{Ga}** $[C_{72}H_{96}Bi_6Cl_8Ga_2N_8]$: C 31.44, H 3.52, N 4.07, found: C 25.34, H 3.30, N 3.38. The EA data match with the compound with three Bismuth. $[C_{72}H_{96}Bi_6Cl_8Ga_2N_8]$3Bi: C 25.60, H 2.87, N 3.32.

IR (ATR) (cm^{-1}): For **8^{Al}** : 3096, 2963, 2926, 2872, 1455, 1376, 1302, 1293, 1230, 1185, 1126, 1060, 983, 914, 762. For **8^{Ga}**: 3099, 2963, 2909, 2867, 1789, 1455, 1376, 1304, 1290, 1252, 1127, 1058, 987, 970, 929, 884, 841, 771, 758, 701.

*Note: A few crystals of complex **8^{AlOAl}** were obtained during the reaction of $AlCl_3$ with **2**. The major product of that reaction is **8^{Al}**. The complex **8^{AlOAl}** features the anion $[(Al_2Cl_5O)_2]^{2-}$ which is formed during the reaction possibly due to traces of air/moisture contamination. Whereas scXRD of **8^{Al}** and **8^{Ga}** only provided connectivity (see Supplementary figures 48 and 49), a full analysis of bond parameters was possible with the complex **8^{AlOAl}** containing the anion $[(Al_2Cl_5O)_2]^{2-}$ (see figure Supplementary 50).*

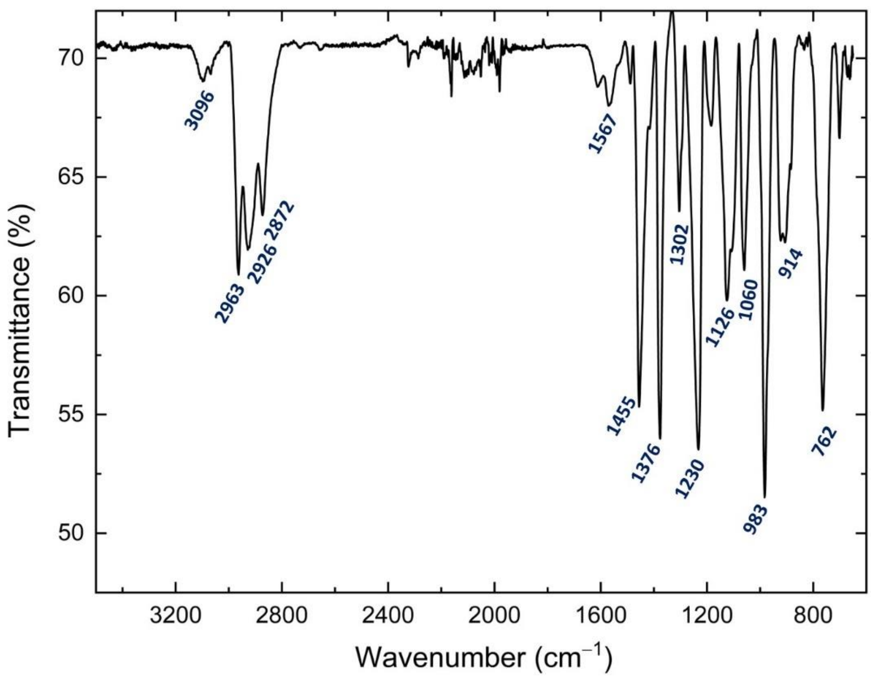

Supplementary Figure 38. FT-IR spectra (ATR) of a solid sample of **8^{Al}**.

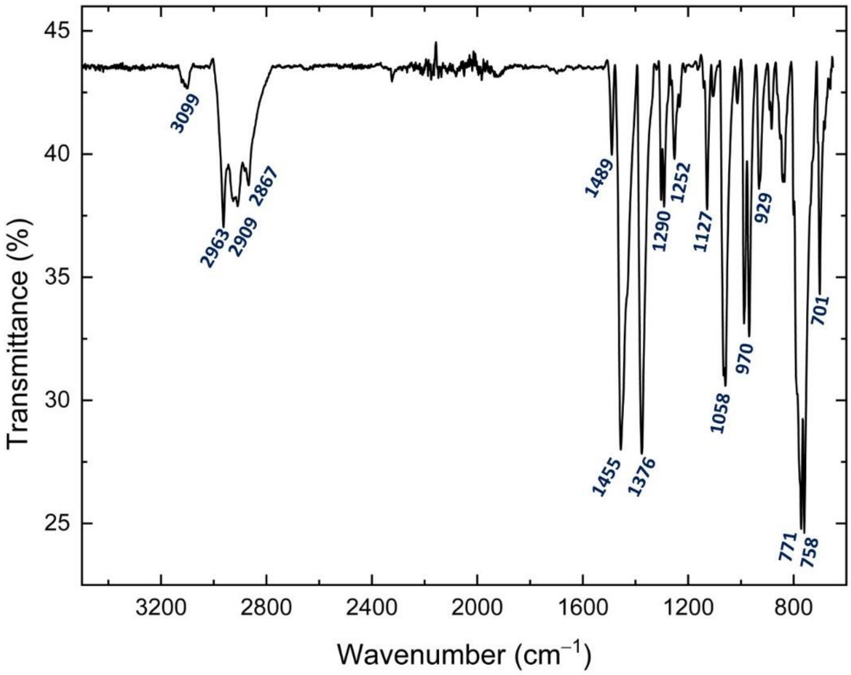

Supplementary Figure 39. FT-IR spectra (ATR) of a solid sample of **8^{Ga}**.

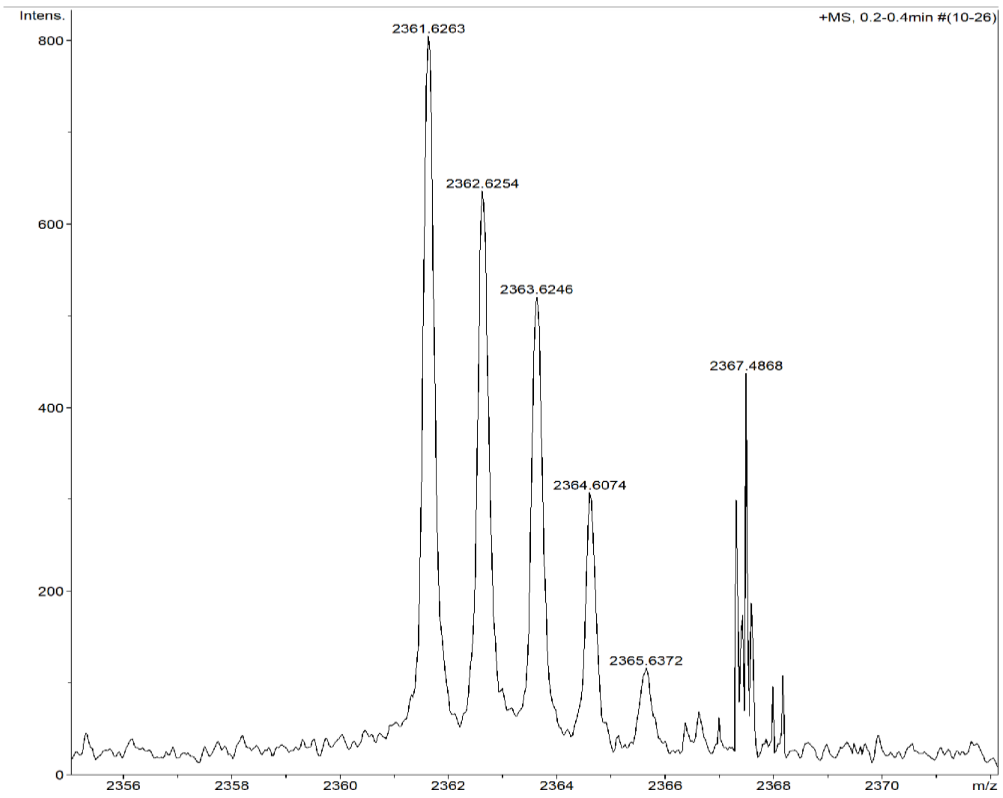

Supplementary Figure 40. Experimental ESI-MS spectrum of $\mathbf{8^{Ga}}$

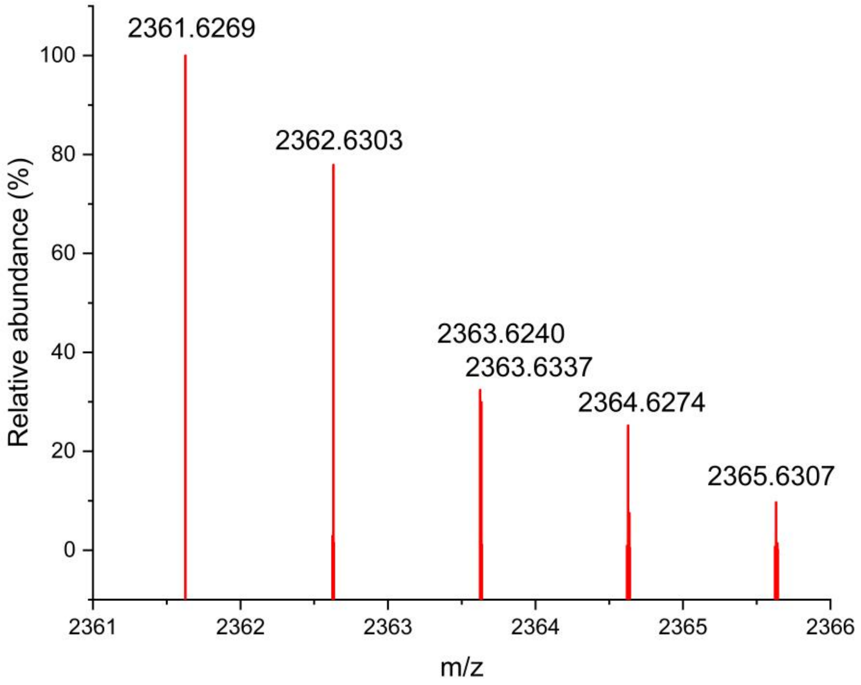

Supplementary Figure 41. Calculated ESI-MS spectrum of $\mathbf{8^{Ga}}$

Characterization of the filtrate: all the solvents were removed from the filtrate, and the residue was dissolved in toluene 5 mL and allowed to stand at room temperature for one day. The mixture was filtered (PPh_4ECl_4 was identified as the residue) and the filtrate contains $\mathbf{4^E}$.

LIFDI Mass Spectrometery in toluene: for $\mathbf{4^E}$: calcd for E = Al [$C_{36}H_{48}AlClN_4$][M]: 598.3383 m/z, found : 598.3341 m/z and calcd for E = Ga [$C_{36}H_{48}GaClN_4$] [M] : 640.2823 m/z, found: 640.2905 m/z.

1.2.9 Synthesis of meso-octaethylcalix[4]pyrrole (H_4EtCx)

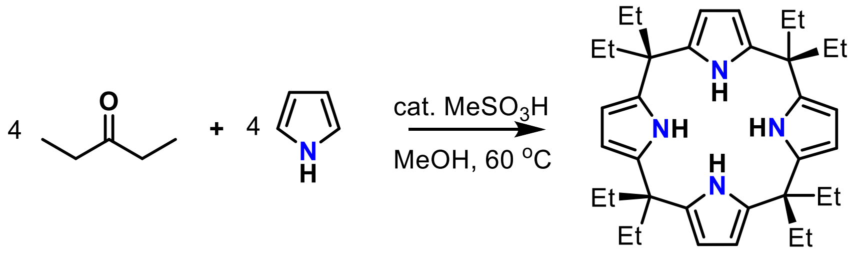

H4EtCx

In a 500 mL round bottom flask, pentan-3-one (19.26 g, 23.77 mL, 223.5 mmol, 1.0 eq) and methanol (180 mL) were mixed. To this flask, freshly distilled pyrrole (15 g, 15.5 mL,223.5 mmol, 1.0 eq) and methanesulfonic acid (4-5 drops) were added. The reaction mixture was stirred for 20 h at 60 °C, during which the reaction mixture turned dark brown, and a colorless solid precipitated. The reaction was cooled to room temperature, and all the volatiles were removed *in-vacuo*. The crude product was purified by flash column chromatography on silica gel with dichloromethane/petroleum ether (40:60) as eluent. The solvent was removed from the combined product fractions and meso-octaethylcalix[4]pyrrole was obtained as a colorless solid (9.5 g, 30% yield).

The analytical data of the product matched with the reported procedure.[1]

^{1}H NMR (200 MHz, CD_2Cl_2, 298 K): δ ^{1}H [ppm] = 6.90 (br s, 4H, N-H), 5.90 (d, $^3J^{HH}$ = 2 Hz, 8H, β-H), 1.80 (br s, 16H, α-methylene), 0.57 (t, $^3J_{HH}$ = 8 Hz, 24H, α-Me).

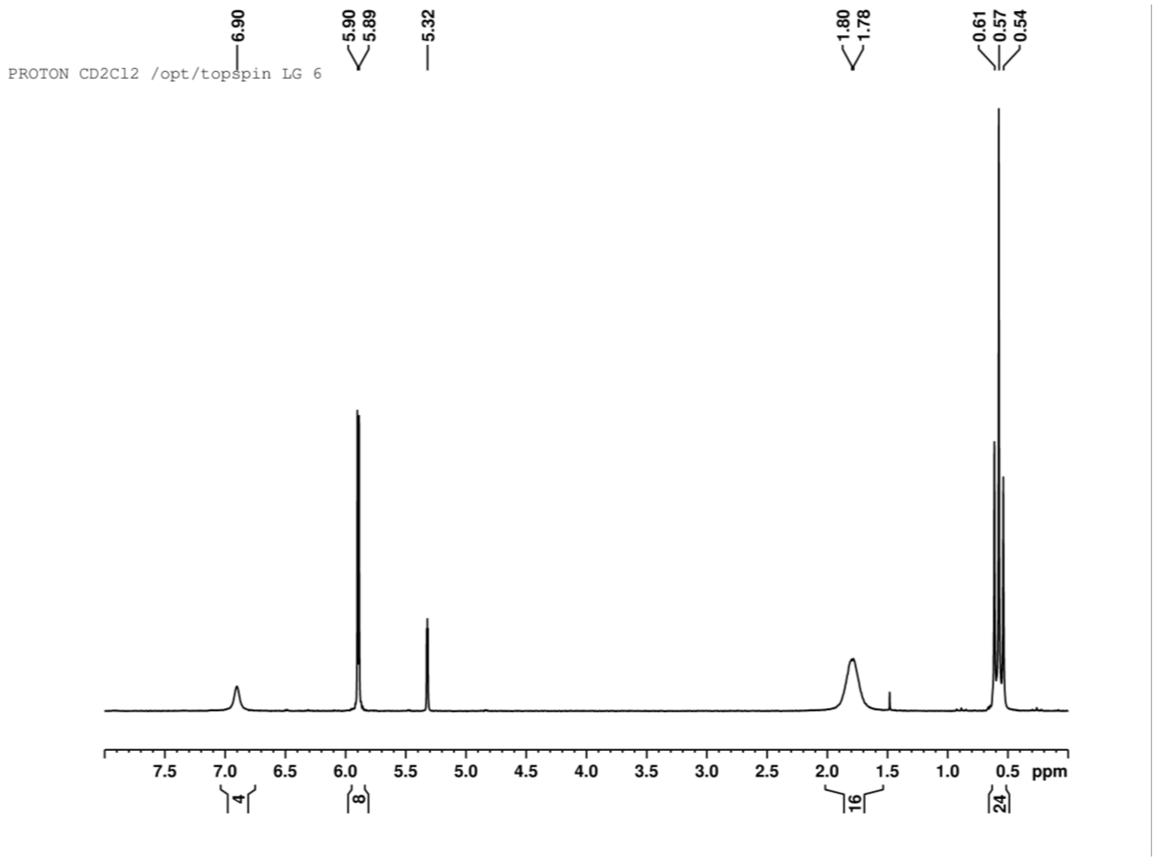

Supplementary Figure 42. ^{1}H NMR (200 MHz, 298 K, CD_2Cl_2) spectrum of H_4EtCx. Signal at 5.32 ppm is due to residual protio solvent.

1.2.10 Synthesis of tetra-lithium-meso-octaethylcalix[4]pyrrolato [Li_4·$(thf)_3$EtCx][2]

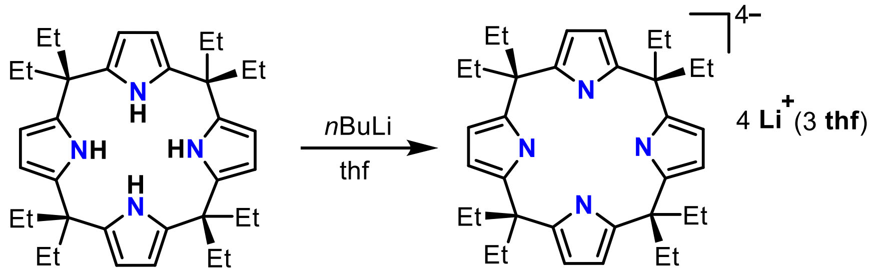

H_4EtCx **$Li_4(thf)_3$EtCx**

In a 250 mL two neck Schlenk flask, 5 g of H_4EtCx (9.24 mmol) was dissolved in thf (50 mL). To this solution *n*-BuLi (1.6 M, 23.11 ml, 36.98 mmol) solution in hexane was added dropwise. One neck of the Schlenk flask was connected to an over-pressure bubbler to release the butane gas formed during the reaction. The reaction mixture was heated to 50 °C for four hours. After that, all the volatiles were removed under reduced pressure to obtain an off

white solid. The residue was washed with 3*15 mL of hexane and dried under vacuum to obtain **[Li_4·(thf)$_3$EtCx]** (6.8 g, 95% yield).

The analytical data of the product matched with the reported procedure.[2]

^{1}H NMR (200 MHz, CD_2Cl_2, 298 K): δ ^{1}H [ppm] = 5.85 (s, 8H, β-H), 3.48 (m, 12H, thf) 1.79 (m, 28H, 16H from α-methylene overlapped with 12H from thf), 0.80 (t, $^3J_{HH}$ = 6 Hz, 24H, α-Me).

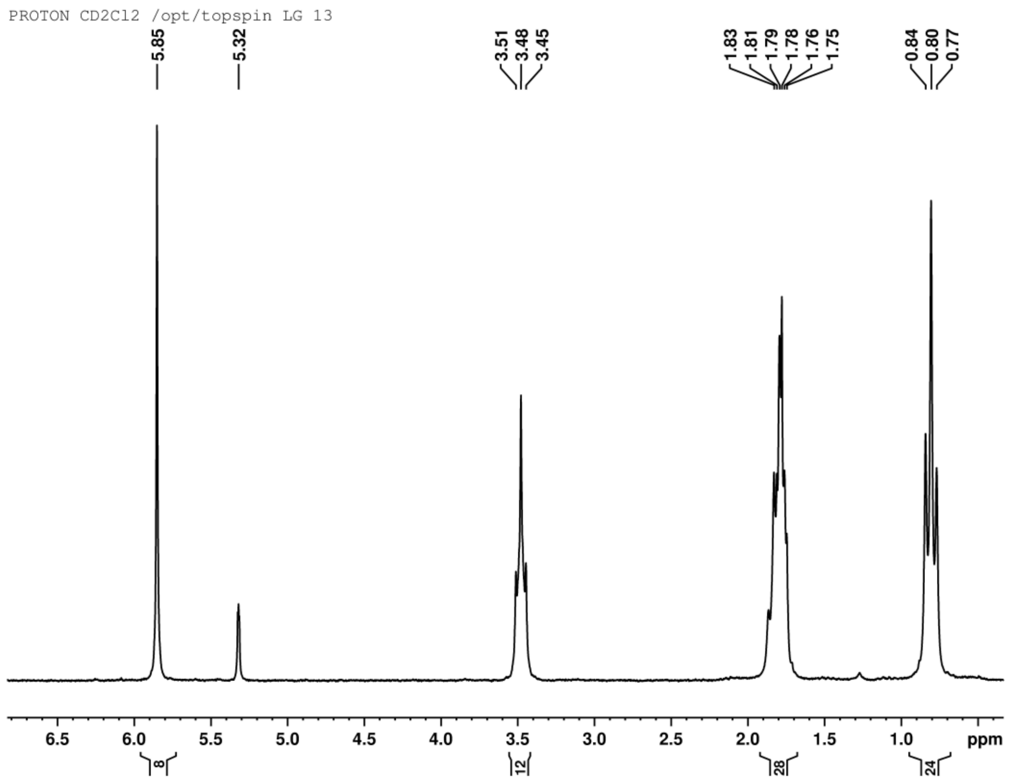

Supplementary Figure 43. ^{1}H NMR (200 MHz, 298 K, CD_2Cl_2) spectrum of [Li_4·(thf)$_3$EtCx]. Signal at 5.32 ppm is due to residual protio solvent.

2. X-Ray Diffraction

Single crystal X-ray diffraction data were collected using Bruker D8 VENTURE with PHOTONIII detector, D8 Goniometer and INCOATEC Mo/Cu microsource. Crystals were selected under perfluoropolyether oil, mounted on 0.1 to 0.3 mm diameter CryoLoops and quench-cooled using an Oxford Cryostream 800 open flow N_2 cooling device.[8] Data were collected at 100 K using monochromated Cu Kα or Mo Kα radiation (λ = 1.5418/0.71073 Å). Data processing was done with SHELXS/XL and refined by least squares on weighted F^2 values for all reflections, disordering of fragments was done with the help of the implemented DSR tool.[9, 10] Finalisation of gathered data was done using final cif tool[11] or Olex2-1.5[12].

Crystallographic data for the structures reported in this paper have been deposited with the Cambridge Crystallographic Data Centre as a supplementary publication. Copies of the data can be obtained free of charge on application to CCDC, 12 Union Road, Cambridge CB21EZ, UK (fax: (+(44)1223-336-033; email: deposit@ccdc.cam.ac.uk).

2.1. Supplementary Table 1. Crystal data and structural refinements

Compound	1	2	3	4	8^{AlOAl}
CCDC number	2264827	2264830	2264829	2264828	2334621
CIF-File	RY139_0	RY142_0	RY203_0	RY203_3	AM234_a
Formula	$C_{44}H_{64}LiN_4O_2Bi$	$C_{60}H_{68}BiClN_4P$	$C_{73}H_{98}N_8In_2Bi_4Br_2Cl_2$	$C_{39}H_{51}BrInN_4$	$C_{37}N_4Al_2Cl_7Bi_3H_{50}O$
$D_{calc.}$/ g cm^{-3}	1.517	1.394	2.169	1.445	2.164
μ/mm^{-1}	4.531	3.484	11.445	1.828	11.950
Formula Weight	896.91	1085.13	2383.87	770.56	2991.71
Colour	red	yellow	red	red	black
Shape	block-shaped	block-shaped	needle-shaped	block-shaped	block-shaped
Size/mm^3	0.19×0.13×0.07	0.21×0.20×0.17	0.24×0.05×0.04	0.13×0.09×0.05	0.20×0.14×0.10
T/K	100(2)	100(2)	100(2)	100.00	120(2)
Crystal System	triclinic	triclinic	monoclinic	monoclinic	triclinic
Space Group	P-1	P-1	$P2_1/n$	$P2_1/n$	P-1
a/Å	11.1439(5)	11.9649(8)	11.6549(10)	11.4969(5)	14.339(3)
b/Å	13.0136(6)	14.0945(8)	18.5943(19)	19.0433(10)	14.366(3)
c/Å	15.1013(7)	16.4809(11)	16.9971(17)	16.2624(8)	14.391(2)
α/°	103.396(2)	105.840(2)	90	90	114.309(7)
β/°	103.492(2)	95.231(3)	97.747(4)	95.902(2)	93.143(7)
γ/°	104.295(2)	101.859(2)	90	90	117.078(7)
V/Å^3	1963.99(16)	2584.5(3)	3649.9(6)	3541.6(3)	2295.8(7)
Z	2	2	2	4	1
Z'	1	1	0.5	1	1
Wavelength/Å	0.71073	0.71073	0.71073	0.71073	0.71073
Radiation type	MoK$_\alpha$	MoK$_\alpha$	MoK$_\alpha$	MoK$_\alpha$	MoK$_\alpha$
Θ_{min}/°	1.982	2.019	1.999	2.072	2.658
Θ_{max}/°	28.786	28.782	28.758	27.160	31.650
Measured Refl's.	92095	161949	176369	113155	154317
Indep't Refl's	10213	13434	9460	7855	15358
Refl's I≥2 σ(I)	9627	12993	8588	7013	13050
R_{int}	0.0574	0.0595	0.0874	0.0746	0.0751
Parameters	523	614	463	423	680
Restraints	288	0	43	0	652
Largest Peak	0.771	1.976	1.259	0.982	2.356
Deepest Hole	-1.179	-1.841	-1.168	-0.997	-2.189
GooF	1.062	1.198	1.114	1.113	1.039
wR_2 (all data)	0.0429	0.0644	0.0549	0.0713	0.0902
wR_2	0.0423	0.0641	0.0529	0.0688	0.0831
R_1 (all data)	0.0202	0.0275	0.0283	0.0343	0.0463
R_1	0.0182	0.0264	0.0240	0.0294	0.0353

2.2. X-ray structures

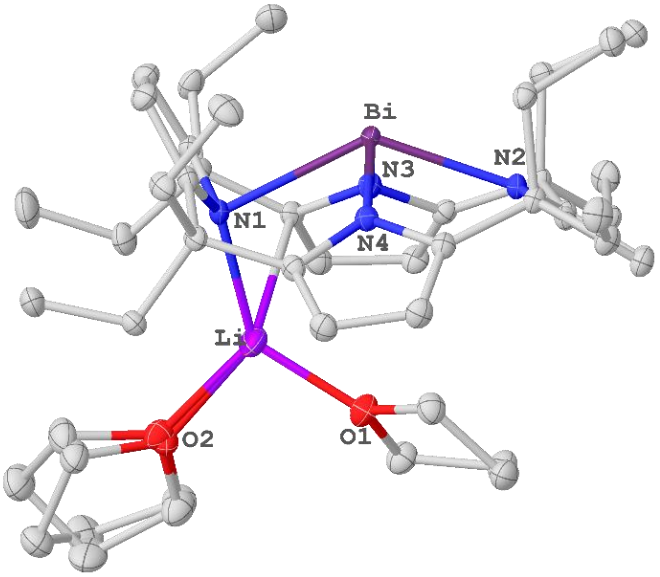

Supplementary Figure 44. Molecular structure of complex **1** in the solid state with thermal ellipsoids at 50% probability. The hydrogen atoms are omitted for clarity.

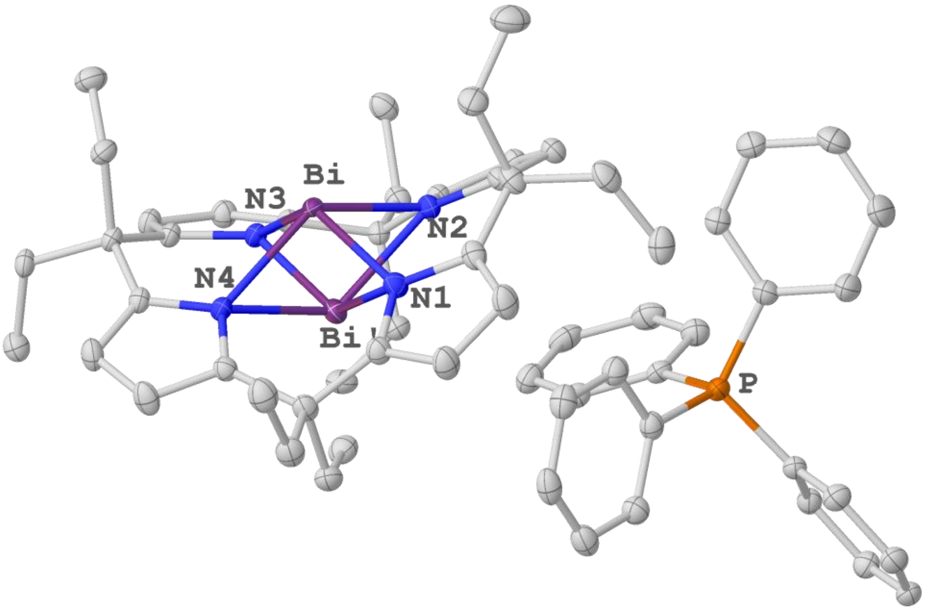

Supplementary Figure 45. Molecular structure of complex **2** in the solid state with thermal ellipsoids at 50% probability. The hydrogen atoms are omitted for clarity. Bi atom is occupying two positions as Bi and Bi'.

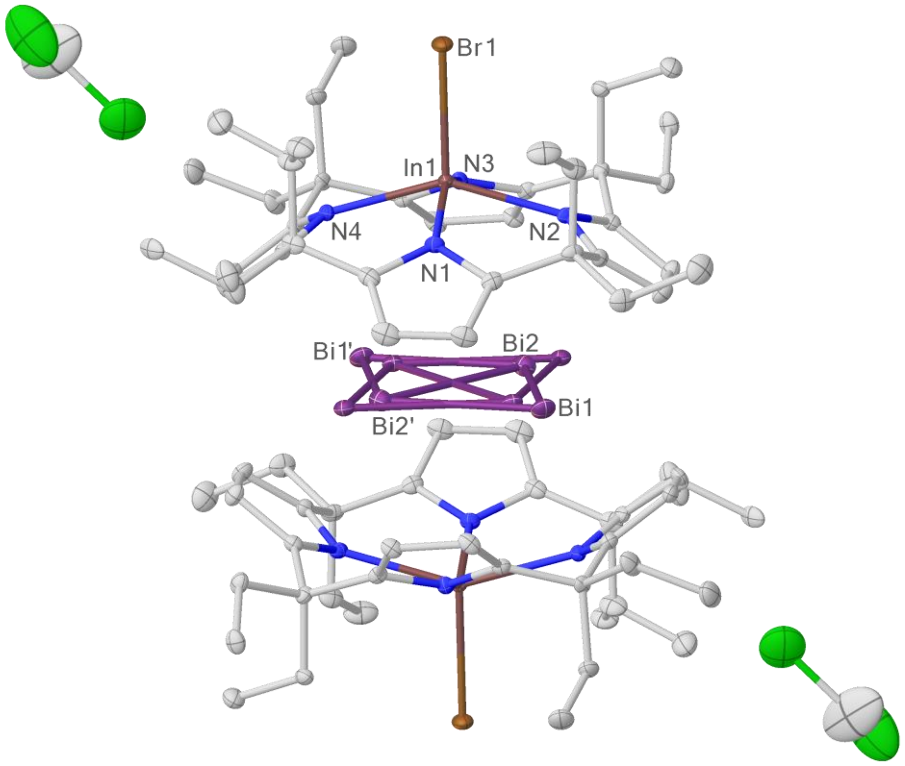

Supplementary Figure46. Molecular structure of complex **3** in the solid state with thermal ellipsoids at 50% probability. The hydrogen atoms are omitted for clarity. $[Bi]_4$ ring is occupying two positions due to rotational disorder.

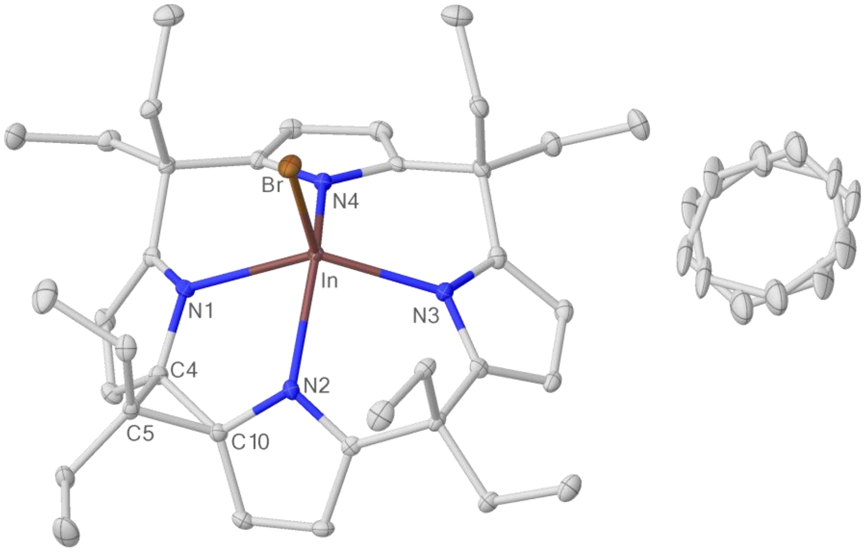

Supplementary Figure 47. Molecular structure of complex **4** in the solid state with thermal ellipsoids at 50% probability. The hydrogen atoms are omitted for clarity. Benzene ring is occupying two positions due to rotational disorder.

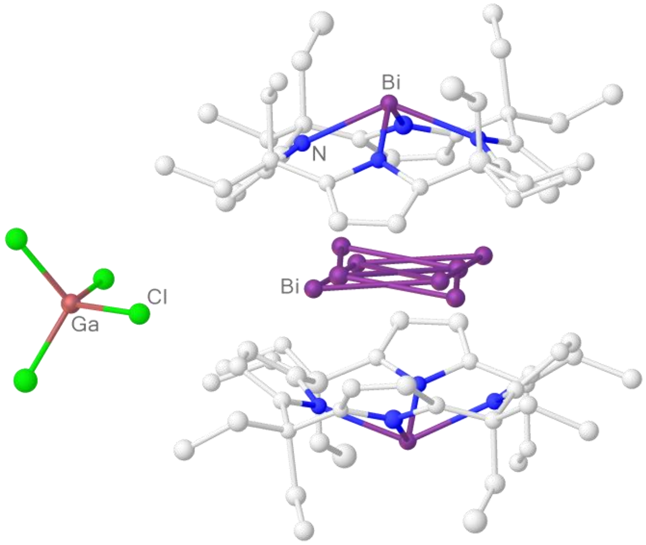

Supplementary Figure 48. Molecular structure of complex $\mathbf{8}^{Ga}$ in the solid state. The current model is obtained from scXRD, however, the quality of data was not good. This is for a pictoral depiction of the molecular structure. Despite several attempts good quality of single crystals of $\mathbf{8}^{Ga}$ could not be obtained.

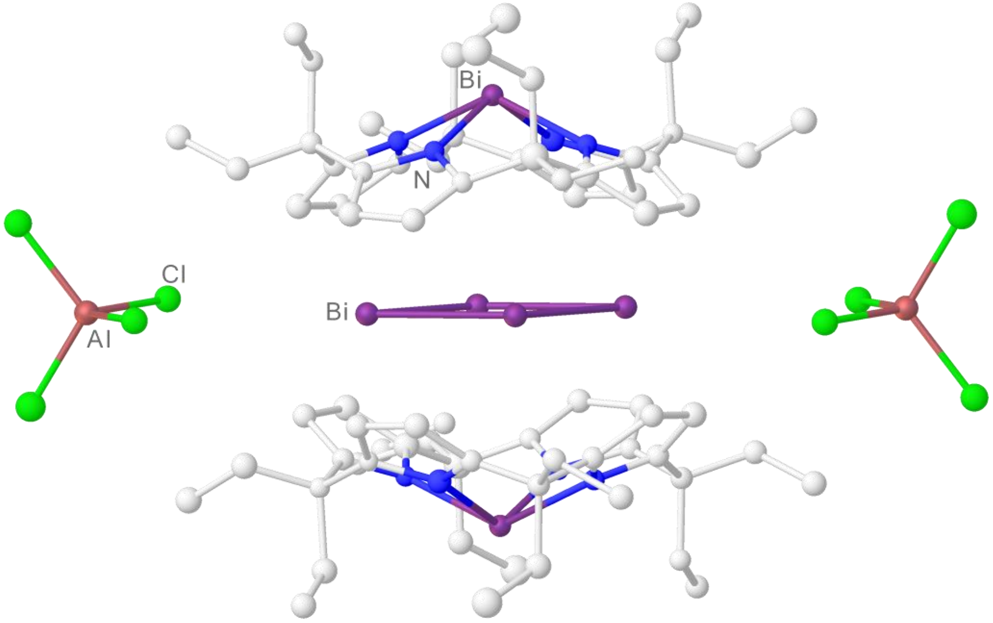

Supplementary Figure 49. Molecular structure of complex $\mathbf{8}^{Al}$ in the solid state. The current model is obtained from scXRD, however, the quality of data was not good. This is for a pictoral depiction of the molecular structure. Despite several attempts good quality of single crystals of $\mathbf{8}^{Al}$ could not be obtained.

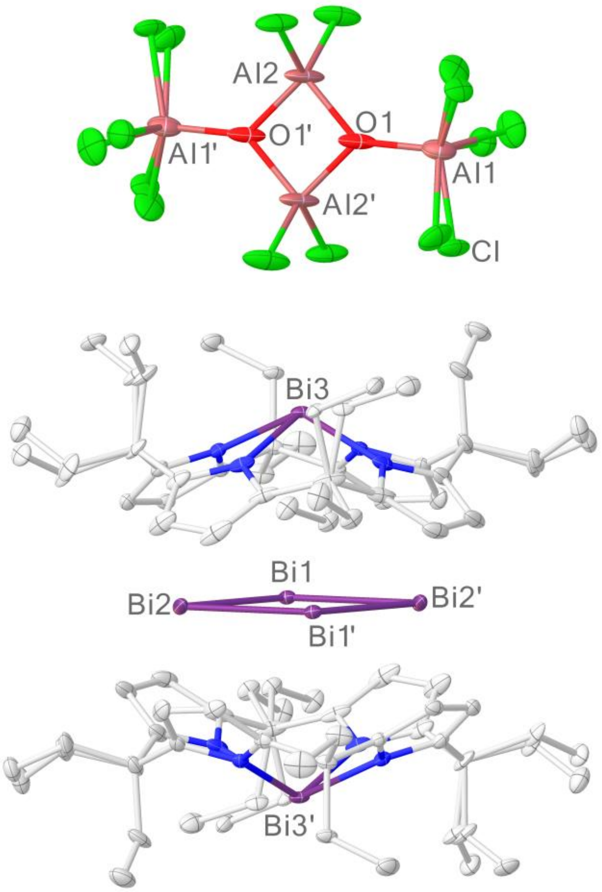

Supplementary Figure 50. Molecular structure of complex **8^{AlOAl}** in the solid state with thermal ellipsoids at 50% probability. The hydrogen atoms and dichloromethane solvents are omitted for clarity.

Comments on Checkcif-Alerts

Compound 3 (RY203_0)

PLAT307_ALERT_2_A Isolated Metal Atom found in Structure (Unusual) Bi1

PLAT307_ALERT_2_A Isolated Metal Atom found in Structure (Unusual) Bi2

PLAT307_ALERT_2_A Isolated Metal Atom found in Structure (Unusual) Bi1’

PLAT307_ALERT_2_A Isolated Metal Atom found in Structure (Unusual) Bi2’

Author Response: This is a structural feature in this compound. The central Bi4_4+ is coordinated between two [calixpyrrole(InBr)]2- ligands via pi-interactions to the pyrrole moieties.

Compound 8^{AlOAl} (AM234_a)

Alert level A

PLAT307_ALERT_2_A Isolated Metal Atom found in Structure (Unusual) Bi1

PLAT307_ALERT_2_A Isolated Metal Atom found in Structure (Unusual) Bi2

Author Response: Bi1 and Bi2 form a [Bi4]4+ unit, which is shielded by two calixpyrrole complexes.

2.3. Discussion of selected metric parameters

Non-VSEPR structure of **2**

The EtCx[4] ligand in **2** enforces a NON-VSEPR square pyramidal geometry at Bi center which lies a distance of 0.9464(2) Å above the N4 plane and the trans N-Bi-N angle of 129.23(8)° and 132.01(8)° respectively.

Comparison of Bi-Bi bond lengths in **3** and **8^{AlOAl}**

Complex	Bi-Bi	Bi-Bi (Diagonal)
**3**	3.0223(3), 3.0238(3)	3.3722(5)
**8^{AlOAl}**	3.0326(6), 3.0384(5)	3.2745(7)

3. Quantum chemical calculations

3.1. General Comments

Calculations were done with the program suite TURBOMOLE,[13] for the title compound employing the PBE functional[14] and def2-TZVP bases[15] together with corresponding effective core potentials[16] and auxiliary basis sets[17] for the Coulomb part, unless explicitly mentioned otherwise. Magnetically induced ring current densities were calculated and integrated with the GIMIC tool.[18] Cartesian coordinates of the optimized structure are available within the ASCII file 'OptimizedStructures.txt' (update at end).

For the detailed study of 4-atomic p-element 16VE species, in the first step a DFT (BP86[19,20]/def-SV(P)[17]) based genetic algorithm[21] was applied to Bi_4^{4+}, Pb_4, Tl_4^{4-} and to Al_4^{4-} in order to identify the relevant structural motifs of 4-atomic 16VE species of p elements. The conductor-like screening model[22] was employed with default parameters throughout. The relevant motifs turned out to be Rhombus (Rh), Butterfly (Bf), Quadrangle (Qu), Rectangle (Re) and Tetrahedron (Td). In the second step, systematic structure optimizations were carried for all these arrangements for these compounds as well as for the homologues of 3p to 5p rows assuming corresponding symmetries (D_{2h} for Rh and Re, C_{2v}, for Bf, D_{4h} for Qu and T_d for Td) for both triplet (T) and singlet (S) states with the PBE functional and dhf-TZVP bases. The Fermi-smearing approach[23] was used, but with the boundary condition to keep the number of unpaired electrons fixed to 0 or 2, respectively. In Supplementary Tables 2-4 we list data for all structures that a) stay in their topologies, b) fulfil the *aufbau* principle and c) for which the neutral species Si_4, Ge_4, Sn_4 and Pb_4 are proven to be minima by calculation of force constants (the calculation of force constants for the highly charged species turned out to be very unreliable, so in case of imaginary frequencies for the neutral species the cat- and anionic isoelectronic counterparts were disregarded as well).

For all cases, the rhombic structure in the electronic singlet state turned out to be most stable, other arrangements are significantly disfavoured throughout. In Supplementary Table 2 energies relative to this geometric/electronic state are given, in Supplementary Table 3 the HOMO-LUMO gaps and in Supplementary Table 4 the lowest vibration frequency of each neutral species. It is evident that obtained Bf arrangements are triplets throughout, whereas Td yield singlets. Re (singlet) and Qu (triplet) turned out to be saddle points throughout. Also, for Rh the lowest energy states are singlets, but here for Al_4^{4-} and the heavier homologues

also triplets are found (with somewhat higher energies); their occurrence is correlated with comparably small HOMO-LUMO gaps for the corresponding singlet states (around 0.6 for Al_4^{4-} and the heavier homologues, but 1.2 to 2.3 eV for the other compounds). In Supplementary Table 5 the magnetically induced ring currents and the nucleus-independent chemical shifts (NICS) are listed for the rhombic arrangements. They show overall diatropic currents between 5 and 15 nA/T which reflects aromatic character for the rhombic structures throughout. This is corroborated by negative NICS values. The Cartesian coordinates of all these structures are sampled in file 'OptimizedStructures.xyz'. Calculations for these structures were repeated with the PBE0 functional[24] with essentially the same outcome, see Supplementary Tables 6-9.

For quantification of the influence of spin-orbit coupling, in the third step the structures obtained so far were further optimized at two-component level[25] (with the PBE functional and dhf-TZVPall bases[26]). This was done only for the 5p and the 6p element compounds (with two-component effective core potentials and corresponding basis sets), and for technical reasons the symmetry was lowered to C_1 for all calculations. Due to spin-orbit coupling most of the triplet states converged to singlet states. The final results after structure optimization are listed in Supplementary Tables 10 to 11. Again, the rhombic structures turned out to be the most favourable one for all six elements, and the energetic distance to the tetrahedron is similar to the one-component case. Moreover, the HOMO-LUMO gaps are similar to the one-component calculation. Low-lying triplet-type states are observed only for In_4^{4-}, for the other cases they converge to singlets during the SCF procedure. In the tables "-" corresponds to: triplet state not converged.

3.2. Computational Data

Supplementary Table 2. Energies (in kJ/mol) with functional PBE for various 16 VE systems relative to the most stable rhombic (Rh) structure for butterfly (Bf), and tetrahedral (Td) arrangements for triplet (T) and singlet (S) states at one-component level. Rh is calculated within D_{2h} symmetry, Bf in C_{2v}, Td in T_d.

		Al_4^{4-}	Si_4	P_4^{4+}	Ga_4^{4-}	Ge_4	As_4^{4+}	In_4^{4-}	Sn_4	Sb_4^{4+}	Tl_4^{4-}	Pb_4	Bi_4^{4+}
Rh	S	0	0	0	0	0	0	0	0	0	0	0	0
	T	11	-	-	11	-	-	13	-	-	14	-	-
Bf	T	31	73	116	27	73	136	21	68	124	17	71	119
Td	S	99	209	317	113	169	225	78	113	149	62	81	109

Supplementary Table 3. HOMO-LUMO gaps (in eV) with functional PBE for various 16 VE systems at one-component level. See also Supplementary Table 2.

		Al_4^{4-}	Si_4	P_4^{4+}	Ga_4^{4-}	Ge_4	As_4^{4+}	In_4^{4-}	Sn_4	Sb_4^{4+}	Tl_4^{4-}	Pb_4	Bi_4^{4+}
Rh	S	0.58	1.24	2.30	0.59	1.25	2.07	0.57	1.17	1.60	0.57	1.24	1.39
	T	0.38	-	-	0.37	-	-	0.29	-	-	0.28	-	-
Bf	T	0.63	0.84	1.04	0.59	0.69	0.77	0.49	0.55	0.36	0.44	0.49	0.16
Td	S	0.49	0.56	0.78	0.54	0.70	0.89	0.53	0.65	0.82	0.58	0.72	0.88

Supplementary Table 4. Lowest vibration frequencies (in 1/cm) with functional PBE for various 16 VE systems at one-component level. See also Supplementary Table 2.

		Si_4	Ge_4	Sn_4	Pb_4
Rh	S	49	45	34	28
Bf	T	175	99	58	39
Td	S	195	113	80	60

Supplementary Table 5. Magnetically induced currents (j, in nA/T) and nucleus-independent chemical shifts at the ring centre, NICS0 (in ppm), as well as 1 Bohr above, NICS1, with functional PBE for rhombic systems in the singlet state at one-component level. See also Supplementary Table 2.

	Al_4^{4-}	Si_4	P_4^{4+}	Ga_4^{4-}	Ge_4	As_4^{4+}	In_4^{4-}	Sn_4	Sb_4^{4+}	Tl_4^{4-}	Pb_4	Bi_4^{4+}
j	14.8	14.6	12.3	13.0	11.9	11.0	10.3	11.0	11.3	5.2	9.0	11.5
NICS0	-3.3	-10.6	-17.6	-3.1	-9.1	-16.0	-2.9	-8.8	-15.9	+2.5	-6.2	-14.5
NICS1	-8.0	-16.1	-21.1	-7.3	-13.4	-18.0	-5.7	-11.3	-16.7	-0.5	-8.4	-15.0

Supplementary Table 6. Energies (in kJ/mol) with functional PBE0 for various 16 VE systems relative to the most stable rhombic (Rh) structure for butterfly (Bf), and tetrahedral (Td) arrangements for triplet (T) and singlet (S) states at one-component level. Rh is calculated within D_{2h} symmetry, Bf in C_{2v}, Td in T_d.

		Al_4^{4-}	Si_4	P_4^{4+}	Ga_4^{4-}	Ge_4	As_4^{4+}	In_4^{4-}	Sn_4	Sb_4^{4+}	Tl_4^{4-}	Pb_4	Bi_4^{4+}
Rh	S	0	0	0	0	0	0	0	0	0	0	0	0
	T	14	-	-	14	-	-	14	-	-	13	-	-
Bf	T	37	78	118	34	76	130	27	70	116	20	72	102
Td	S	102	220	342	121	184	249	89	126	163	73	93	117

Supplementary Table 7. HOMO-LUMO gaps (in eV) with functional PBE0 for various 16 VE systems at one-component level. See also Supplementary Table 6.

		Al_4^{4-}	Si_4	P_4^{4+}	Ga_4^{4-}	Ge_4	As_4^{4+}	In_4^{4-}	Sn_4	Sb_4^{4+}	Tl_4^{4-}	Pb_4	Bi_4^{4+}
Rh	S	1.82	2.81	4.16	1.83	2.73	3.63	1.70	2.48	2.93	1.66	2.51	2.63
	T	1.54	-	-	1.53	-	-	1.40	-	-	1.40	-	-
Bf	T	1.88	2.46	3.01	1.83	2.25	2.43	1.63	1.92	1.77	1.56	1.79	1.59
Td	S	1.68	2.03	2.44	1.70	2.05	2.39	1.57	1.84	2.14	1.59	1.87	2.15

Supplementary Table 8. Lowest vibration frequencies (in 1/cm) with functional PBE0 for various 16 VE systems at one-component level. See also Supplementary Table 6.

		Si_4	Ge_4	Sn_4	Pb_4
Rh	S	71	52	38	30
Bf	T	177	101	59	51
Td	S	229	128	88	64

Supplementary Table 9. Magnetically induced currents (j, in nA/T) and nucleus-independent chemical shifts at the ring centre, NICS0, as well as 1 Bohr above, NICS1, with functional PBE0 for rhombic systems in the singlet state at one-component level. See also Supplementary Table 6.

	Al_4^{4-}	Si_4	P_4^{4+}	Ga_4^{4-}	Ge_4	As_4^{4+}	In_4^{4-}	Sn_4	Sb_4^{4+}	Tl_4^{4-}	Pb_4	Bi_4^{4+}
j	15.9	15.6	13.5	14.6	11.9	12.1	12.2	12.5	12.3	6.5	9.7	12.0
NICS0	-5.6	-13.2	-21.0	-5.8	-12.1	-19.2	-6.2	-11.7	-18.7	-0.0	-8.1	-16.2
NICS1	-10.2	-18.5	-24.2	-9.9	-16.2	-21.0	-8.8	-14.1	-19.3	-2.8	-10.2	-16.7

Supplementary Table 10. Energies (in kJ/mol) at two-component level with the PBE functional for various 16 VE systems relative to the most stable rhombic (Rh) structure for butterfly (Bf), and tetrahedral (Td) arrangements. "S" denotes states with expectation values for the integrated spin density $|S|\approx 0$, T such with $|S|\approx 2$. All structures were optimized in C_1 symmetry.

		In_4^{4-}	Sn_4	Sb_4^{4+}	Tl_4^{4-}	Pb_4	Bi_4^{4+}
Rh	S	0	0	0	0	0	0
	T	15	-	-	-	-	-
Bf	T	24	68	-	-	-	-
Td	S	77	103	132	49	44	46

Supplementary Table 11. HOMO-LUMO gaps (in eV) for various 16 VE systems at two-component level with the PBE functional. See also Supplementary Table 10.

		In_4^{4-}	Sn_4	Sb_4^{4+}	Tl_4^{4-}	Pb_4	Bi_4^{4+}
Rh	S	0.57	1.20	1.60	0.71	1.57	1.63
	T	0.28	-	-	-	-	-
Bf	T	0.49	0.54	-	-	-	-
Td	S	0.50	0.62	0.79	0.60	0.86	1.20

3.3. Computed UV-Vis spectrum of 3

Electronic excitations were calculated at level PBE0/dhf-TZVP. In Fig. S49 the ten lowest singlet excitations are shown together with the intensity-weighted summed difference of non-relaxed densities of these ten lowest excited states to the ground state. The excitations exhibit a clear charge-transfer character from the pi-systems of the calixpyrroles to the HOMO of the Bi_4 ring. The lowest triplet excitations are at 1.49 and 1.52 eV.

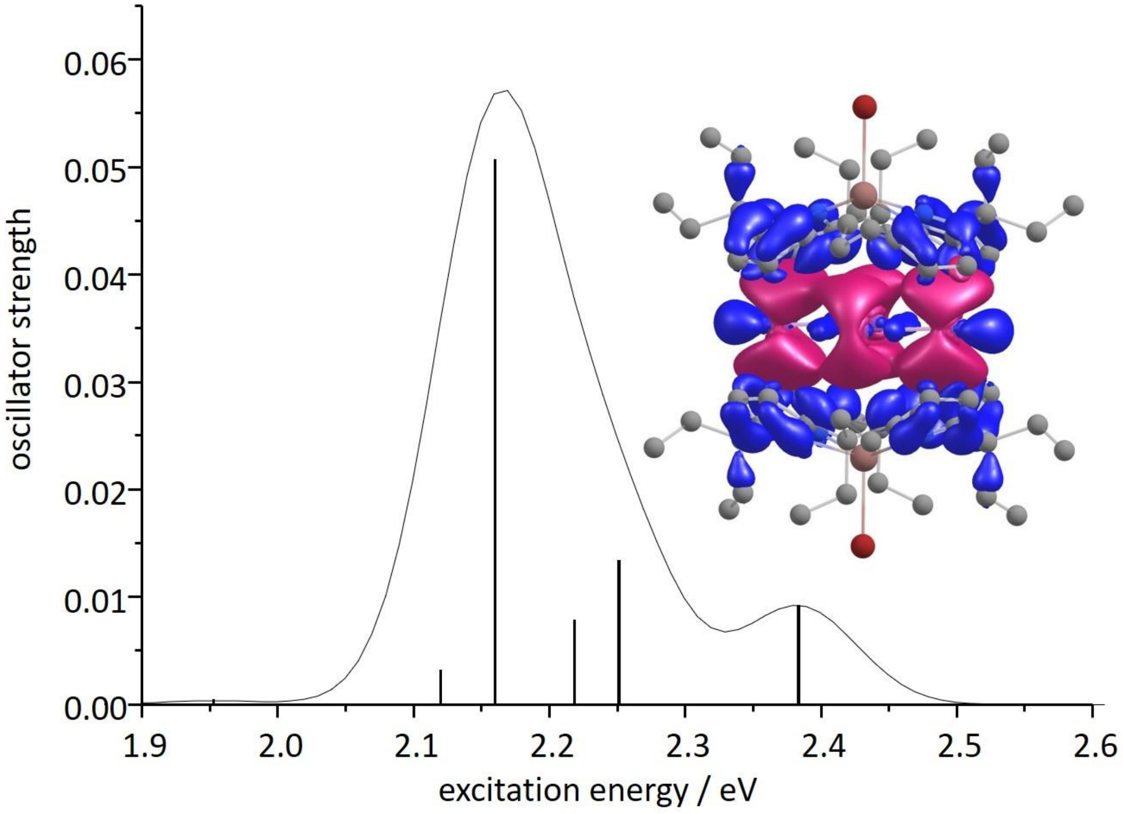

Supplementary Figure 51. Calculated ten lowest singlet excitations and oscillator-strength-weighted summed difference of densities of the corresponding excited states and the ground

state; blue colour indicates a surplus of electrons for the ground state, red for the excited state. Contours are drawn at 0.0005 a.u.

3.4. Vibrational Spectroscopy

Vibration spectra were calculated at level PBE/dhf-TZVP. The bare D_{2h} symmetric Bi_4^{4+} ring shows 6 vibrations that transform like Raman active A_g(2) and B_{1g} as well as IR active B_{1u}, B_{2u}, B_{3u} and B_{1g}. They show frequencies between 41 and 133 1/cm that also can be identified as dominant parts of vibrations of the entire system **3**. Sketches of the vibrations and their frequencies in the isolated Bi_4^{4+} ring as well as in **3** are listed in Supplementary Table 12.

Supplementary Table 12. Vibrations in bare Bi_4^{4+} and in **3**. The classification refers to D_{2h} symmetry, frequencies are given in 1/cm. For 3, those vibrations are listed for which the corresponding vibration of the Bi_4 unit dominates.

	A_g	A_g	B_{1g}	B_{1u}	B_{2u}	B_{3u}
	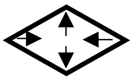	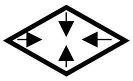	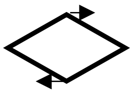	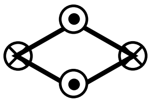	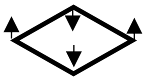	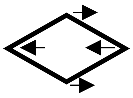
Bi_4^{4+}	89	132	108	41	52	131
**3**	69	125,133	94,99	45	90	101,107

The Raman active bands Ag (125,133) and B1g (94,99) match well with the experimentally observed bands in a sample of **3** (135, 93), see also Supplementary figure 14.

3.5. Bond analysis: Localized MOs, Bond-critical points, energy decomposition analysis and a simple electrostatic model

In the main document it was demonstrated that the canonical MOs of the Bi_4^{4+} ring are found – with slight modifications – also in the entire compound **3**. This is also evident from the comparison of localized MOs, see Supplementary Figure 52.

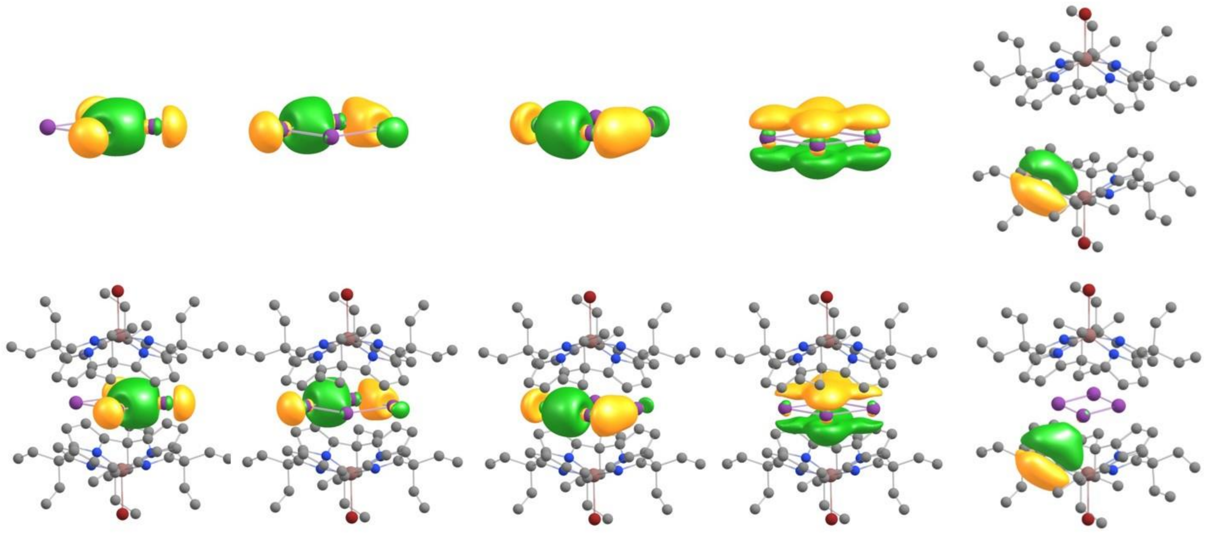

Supplementary Figure 52. Pipek-Mezey[27] localized MOs of the separate parts of 3 (upper row) and of the entire system. Contours are drawn at 0.04 a.u. The four MOs at the left hand side are the only ones without contribution from the ligands, the rightmost one is one of eight LMOs representing a pi-type bond at the ligand that is slightly delocalized towards the Bi_4^{4+} ring.

By the localization procedure, the 6p orbitals of the bare Bi_4^{4+} ring are transformed to three sigma-type orbitals and one π-type orbital (the four orbitals at the left in the upper row). These orbitals are found essentially unchanged among the LMOs of the entire compound (the four orbitals at the left in the lower row). Slight differences between the separate systems and the entire system are found for eight LMOs representing π-type bonds in the ligands (rightmost orbital in the upper and the lower row shown as representative). Here one observes a slight delocalization towards the Bi_4^{4+} ring if present, with Mulliken contributions of Bi amounting to ~0.20 electrons. These overall 1.6 electrons are the main reason for the difference between the charge of +4 for the idealized description and the calculated charge of +2.3.

Further, we carried out a topological analysis of the electron density (with the proper module of TURBOMOLE). It yields 16 bond-critical points between the Bi_4 unit and the calixpyrroles (PBE/dhf-TZVP, without COSMO). Their positions are shown in Fig. S51, the densities and the eigenvalues of its second derivative are sampled in Supplementary Table 13, together with that for Bi_4Me_4 and BiCp* (C_s-symmetric, with Bi bond to one of the C atoms) for comparison.

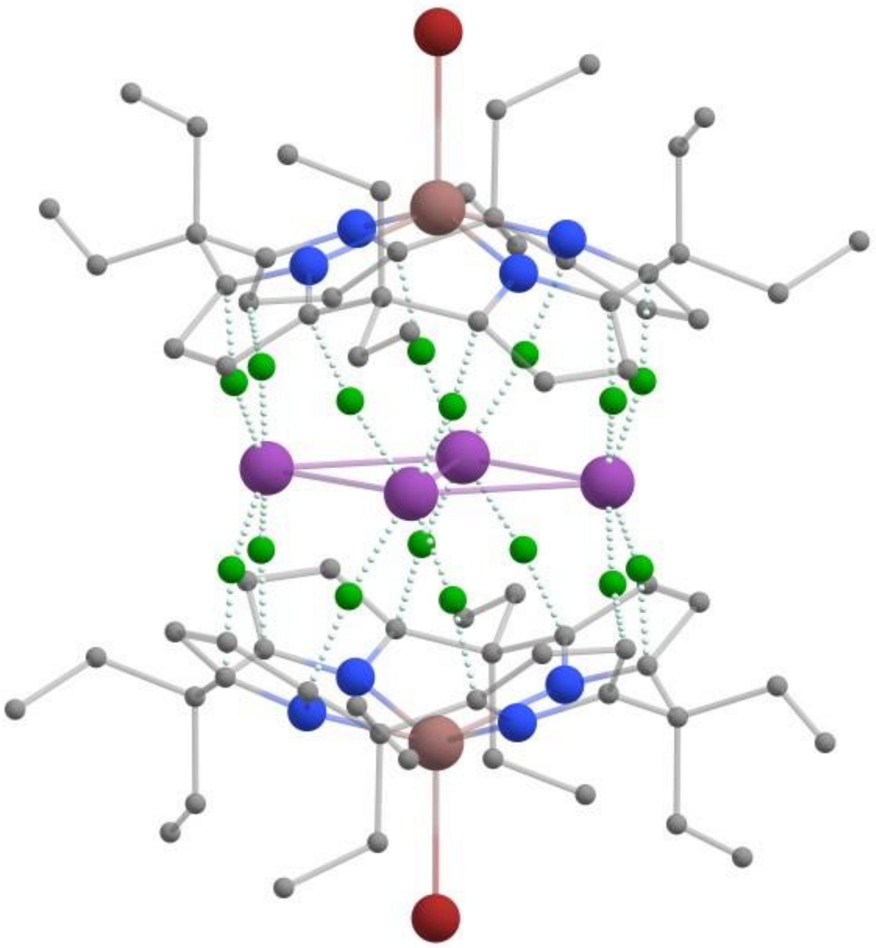

Supplementary Figure 53. Positions of bond-critical points (green) in compound 3. The dotted lines are drawn to help the eye.

For **3**, with shortest Bi-C distances of ~300 pm, the density amounts to ca. 0.02 a.u. at the BCPs, which is four times smaller than for the Bi-C bond in Bi_4Me_4 ($d_{Bi\text{-}C}$=231 pm) or in BiCp* ($d_{Bi\text{-}C}$=239 pm), which indicates a more ionic character for the title compound than for the others. Moreover, the ratio of the positive eigenvalue (the one along the connecting axis, last column) and the two others is significantly higher for the title compound than for Bi_4Me_4 or BiCp*, which also indicates a higher ionic character for the title compound.

Supplementary Table 13. Densities and eigenvalues of the 2nd derivative of the density at the 16 BCPs found between the Bi4 unit and the calixpyrroles. In the last line the corresponding numbers for the Bi-C bonds in Bi_4Me_4 are given.

	ρ	EV1	EV2	EV3
1	1.630E-02	-9.947E-03	-6.581E-03	4.414E-02
2	1.669E-02	-1.050E-02	-6.310E-03	4.484E-02
3	1.629E-02	-1.016E-02	-6.188E-03	4.389E-02
4	1.629E-02	-1.033E-02	-5.671E-03	4.357E-02

5	2.079E-02	-1.452E-02	-5.941E-03	5.332E-02
6	2.093E-02	-1.463E-02	-5.622E-03	5.317E-02
7	2.104E-02	-1.473E-02	-5.568E-03	5.335E-02
8	2.132E-02	-1.491E-02	-5.626E-03	5.395E-02
9	1.629E-02	-1.016E-02	-6.188E-03	4.389E-02
10	1.629E-02	-1.033E-02	-5.671E-03	4.357E-02
11	1.669E-02	-1.050E-02	-6.311E-03	4.484E-02
12	1.630E-02	-9.947E-03	-6.581E-03	4.414E-02
13	2.104E-02	-1.473E-02	-5.567E-03	5.335E-02
14	2.093E-02	-1.463E-02	-5.622E-03	5.317E-02
15	2.133E-02	-1.492E-02	-5.626E-03	5.395E-02
16	2.079E-02	-1.452E-02	-5.941E-03	5.332E-02
Bi_4Me_4	8.761E-02	-8.820E-02	-8.784E-02	2.470E-01
BiCp*	7.308E-02	-6.914E-02	-6.110E-02	2.049E-01

The ionic character is further supported by an energy decomposition analysis[28] $Bi_4L_2 \rightarrow Bi_4^{4+} + 2L^{2-}$, yielding a total interaction energy 4998 kJ/mol with 82% ionic contribution. Of course, when compensating the charge with COSMO, the interaction energy is much smaller, 926 kJ/mol for infinite dielectric constant, or any value in-between, depending on the choice of the dielectric constant.

Finally, a simple purely electrostatic consideration yields a very similar result: If one reduces the Bi_4 unit to a point charge of –4 a.u. and the two calixpyrroles to point charges of –2 a.u. and takes the distance between them roughly as found in **3**, 7 a.u., the electrostatic interaction between these charges amounts to $2\cdot\frac{(-2)\cdot 4}{7}+\frac{(-2)\cdot(-2)}{14}=-2$ a.u.= –5251 kJ/mol.

3.6. Deformation of "ruffled" to "domed" calix[4]pyrrolates

A hint for the specific role of In-Br – as compared to lighter homologues – in the formation of **3** may be seen in the thermodynamic preference of the "domed" arrangement (left hand side of Supplementary Figure 54) of the dianionic calix[4]pyrrolate over the "ruffled" one (right hand side of Supplementary Figure 54. While the "domed" isomer, which shows essentially the same form as the calix[4]pyrrolate in **3**, is energetically preferred for In-Br by 21 kJ/mol, it is clearly de-preferred for Al-Cl, Ga-F or Ga-Cl by 69/64/43 kJ/mol (calculated at level PBE/dhf-TZVP with the conductor-like screening model with infinite dielectric constant).

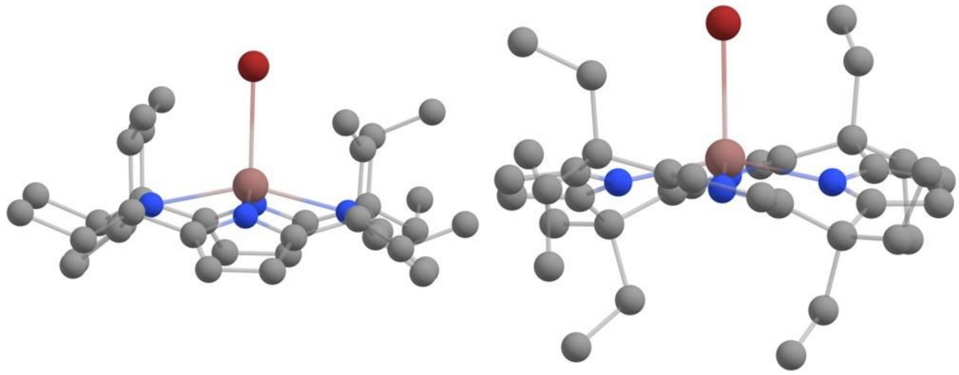

Supplementary Figure 54. Domed (right) and ruffled configuration of dianionic calix[4]pyrrolate, exemplarily shown for In (dark beige) and Br (brown). Hydrogen atoms are omitted for clarity.

3.7. Influence of the Bi_4 unit on the ^{13}C shifts

For probing the influence of the Bi_4 unit on the ^{13}C shifts of the calixpyrrolate units we calculated them for **3** as well as for **3** with Bi_4^{4+} being replaced with four Xe atoms. This was done within an all-electron relativistic treatment (exact two-component decoupling in the diagonal local approximation for thee unitary transformation matrix, DLU-X2C[29]), employing x2c-TZVPall bases[30] and the PBE0 functional. The result is shown in Fig. S53. The computed values of **3** correspond well with the experimental values (cf. Supplementary Figure 15).

The comparison with four Xe-atoms vs. the Bi_4^{4+} allowed screening the influence of Bi on the unusual down-field shift of the carbon signals to >180 ppm. While for C atoms at positions A-E the shifts do not significantly depend on the type of the four-atomic unit in the center, those at positions F and G (the positions closest to the four Bi/Xe atoms) are significantly influenced. With Xe4 in the centre, they are almost degenerate at 155 ppm, while for Bi_4^{4+} the signals are significantly split to 189 ppm for F and 140 ppm for G.

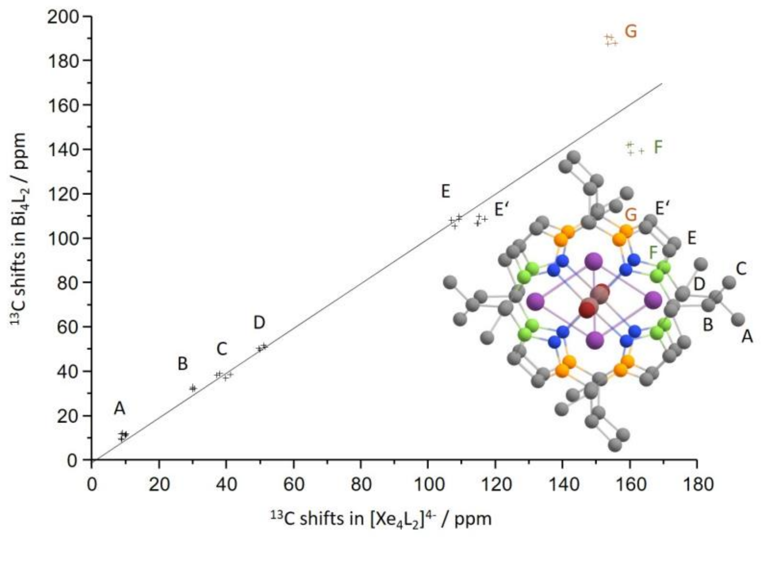

Supplementary Figure 55. ^{13}C shifts for **3** as well as for **3** with Bi_4^{4+} being replaced with Xe_4.

4. References

1. Sigmund L. M., Ehlert C., Enders M., Graf J., Gryn'ova G., Greb L. Dioxygen Activation and Pyrrole α-Cleavage with Calix[4]pyrrolato Aluminates: Enzyme Model by Structural Constraint. *Angew. Chem. Int. Ed.* **60**, 15632-15640 (2021).

2. Jacoby D., Floriani C., Chiesi-Villa A., Rizzoli C. Zirconium meso-octaethylporphyrinogen as a carrier for sodium hydride in toluene: zirconium-sodium bimetallic hydride and alkyls. *J. Am. Chem. Soc.* **115**, 3595-3602 (1993).

3. Doebelin N., Kleeberg R. Profex: a graphical user interface for the Rietveld refinement program BGMN. *J. Appl. Cryst.* **48**, 1573-1580 (2015).

4. Altomare A., Cuocci C., Giacovazzo C., Moliterni A., Rizzi R. QUALX: a computer program for qualitative analysis using powder diffraction data. *J. Appl. Cryst.* **41**, 815-817 (2008).

5. http://www.crystallography.net/cod/9008576.html.

6. Altomare A., Corriero N., Cuocci C., Falcicchio A., Moliterni A., Rizzi R. QUALX2.0: a qualitative phase analysis software using the freely available database POW_COD. *J. Appl. Cryst.* **48**, 598-603 (2015).

7. Transue W. J., Dai Y., Riu M.-L. Y., Wu G., Cummins C. C. 31P NMR Chemical Shift Tensors: Windows into Ruthenium Phosphinidene Complex Electronic Structures. *Inorg. Chem.* **60**, 9254-9258 (2021).

8. Cosier J., Glazer A. M. A nitrogen-gas-stream cryostat for general X-ray diffraction studies. *J. Appl. Cryst.* **19**, 105-107 (1986).

9. Sheldrick G. A short history of SHELX. *Acta Crystallogr., Sect. A* **64**, 112-122 (2008).

10. Sheldrick G. Crystal structure refinement with SHELXL. *Acta Crystallogr., Sect. C* **71**, 3-8 (2015).

11. Kratzert D. *https://dkratzert.de/finalcif.html*

12. Dolomanov O. V., Bourhis L. J., Gildea R. J., Howard J. A. K., Puschmann H. OLEX2: a complete structure solution, refinement and analysis program. *J. Appl. Cryst.* **42**, 339-341 (2009).

13. TURBOMOLE v. 7.7 (University of Karlsruhe and Forschungszentrum Karlsruhe GmbH, 1989-2007), TURBOMOLE GmbH since 2007. Available from https://turbomole.org, 2022).

14. Perdew J. P., Burke K., Ernzerhof M. Generalized Gradient Approximation Made Simple. *Phys. Rev. Lett.* **77**, 3865-3868 (1996).

15. Weigend F., Ahlrichs, R. Balanced basis sets of split valence, triple zeta valence and quadruple zeta valence quality for H to Rn: Design and assesmant of accuracy. *Phy. Chem. Chem. Phys*. **7**, 3207-3305 (2005).

16. Andrae D., Häußermann U., Dolg M., Stoll H., Preuß H. Energy-adjustedab initio pseudopotentials for the second and third row transition elements. *Theoret. Chim. Acta* **77**, 123-141 (1990).

17. Eichkorn K., Weigend F., Treutler O., Ahlrichs R. Auxiliary basis sets for main row atoms and transition metals and their use to approximate Coulomb potentials. *Theor. Chem. Acc.* **97**, 119-124 (1997).

18. Jusélius J., Sundholm D., Gauss J. Calculation of current densities using gauge-including atomic orbitals. *J. Chem. Phys.* **121**, 3952-3963 (2004). Available from https://github.com/qmcurrents/gimic.

19. Becke A. D. Density-functional exchange-energy approximation with correct asymptotic behavior. *Phys. Rev. A* **38**, 3098-3100 (1988).

20. Perdew J. P. Density-functional approximation for the correlation energy of the inhomogeneous electron gas. *Phys. Rev. B* **33**, 8822-8824 (1986).

21. Sierka M., Döbler J., Sauer J., Santambrogio G., Brümmer M., Wöste L.*, et al.* Unexpected Structures of Aluminum Oxide Clusters in the Gas Phase. *Angew. Chem. Int. Ed.* **46**, 3372-3375 (2007).

22. Schäfer A., Klamt A., Sattel D., Lohrenz J. C. W., Eckert F. COSMO Implementation in TURBOMOLE: Extension of an efficient quantum chemical code towards liquid systems. *Phys. Chem. Chem. Phys.* **2**, 2187-2193 (2000).

23. Kresse G., Furthmüller J. Efficiency of ab-initio total energy calculations for metals and semiconductors using a plane-wave basis set. *Comput. Mater. Sci.* **6**, 15-50 (1996).

24. Perdew J. P., Ernzerhof, M., Burke K. Rationale for mixing exact exchange with density functional approximations. *J. Chem. Phys.* **105**, 9982-9985 (1996).

25. Baldes A., Weigend F. Efficient two-component self-consistent field procedures and gradients: implementation in TURBOMOLE and application to. *Mol. Phys.* **111**, 2617-2624 (2013).

26. Weigend F., Baldes A. Segmented contracted basis sets for one- and two-component Dirac–Fock effective core potentials. *J. Chem. Phys.* **133**, 174102 (2010).

27. Pipek, J., Mezey, P. G. A fast intrinsic localization procedure applicable for ab initio and semiempirical linear combination of atomic orbital wave functions. J. Chem. Phys. **90**, 4916-4926 (1989).

28. Kitaura, K., Morokuma, K. New energy decomposition scheme for molecular interactions within Hartree-Fock approximation. *Int. J. Quantum Chem*. **10**,325-340 (1976).

29. Franzke, Y. J., Weigend, F. NMR Shielding Tensors and Chemical Shifts in Scalar-Relativistic Local Exact Two-Component Theory. *J. Chem. Theory Comput*. **15**, 1028–1043 (2019).

30. Pollak, P., Weigend, F. Segmented Contracted Error-Consistent Basis Sets of Double- and Triple-ζ Valence Quality for One- and Two-Component Relativistic All-Electron Calculations. *J. Chem. Theory Comput*. **13**, 3696-3705 (2017).
